# Natural History of Progression of HPV Infection to Cervical Lesion or Clearance: Analysis of the Control Arm of the Large, Randomised PATRICIA Study

**DOI:** 10.1371/journal.pone.0079260

**Published:** 2013-11-19

**Authors:** Unnop Jaisamrarn, Xavier Castellsagué, Suzanne M. Garland, Paulo Naud, Johanna Palmroth, Maria Rowena Del Rosario-Raymundo, Cosette M. Wheeler, Jorge Salmerón, Song-Nan Chow, Dan Apter, Julio C. Teixeira, S. Rachel Skinner, James Hedrick, Anne Szarewski, Barbara Romanowski, Fred Y. Aoki, Tino F. Schwarz, Willy A. J. Poppe, F. Xavier Bosch, Newton S. de Carvalho, Maria Julieta Germar, Klaus Peters, Jorma Paavonen, Marie-Cecile Bozonnat, Dominique Descamps, Frank Struyf, Gary O. Dubin, Dominique Rosillon, Laurence Baril, HPV PATRICIA Study Group

**Affiliations:** 1 Department of Obstetrics and Gynaecology, Faculty of Medicine, Chulalongkorn University, Bangkok, Thailand; 2 Unit of Infections and Cancer, Cancer Epidemiology Research Program, Institut Català d’Oncologia (ICO), IDIBELL, CIBER-ESP, L’Hospitalet de Llobregat, Catalonia, Spain; 3 Department of Microbiology and Infectious Diseases, The Royal Women's Hospital, Parkville/Department of Microbiology, The Royal Children's Hospital, Parkville/Murdoch Childrens Research Institute, Parkville/Department of Obstetrics and Gynaecology, University of Melbourne, Parkville, Victoria, Australia; 4 Department of Gynecology & Obstetrics, Federal University of Rio Grande do Sul, UFRGS/HCPA - Hospital de Clínicas de Porto Alegre, Porto Alegre, Brazil; 5 Central Hospital of North Carelian, Department of Obstetrics and Gynecology, Joensuu, Finland; 6 San Pablo Colleges Medical Center, San Pablo City, Laguna, Philippines; 7 Departments of Pathology and Obstetrics and Gynecology, University of New Mexico Health Sciences Center, Albuquerque, New Mexico, United States of America; 8 Unidad de Investigación Epidemiológica y en Servicios de Salud, Instituto Mexicano del Seguro Social, Morelos, Mexico; 9 Department of Obstetrics and Gynecology, College of Medicine and the Hospital, National Taiwan University, Taipei, Taiwan; 10 Family Federation of Finland, Sexual Health Clinic, Helsinki, Finland; 11 Departamento de Tocoginecologia da Unicamp, University of Campinas, Campinas, Sao Paulo, Brazil; 12 Vaccines Trials Group, Telethon Institute for Child Health Research, Perth, Western Australia; 13 Sydney University Discipline of Paediatrics and Child Health, Children’s Hospital, Westmead, Sydney, New South Wales, Australia; 14 Kentucky Pediatric and Adult Research, Bardstown, Kentucky, United States of America; 15 Centre for Cancer Prevention, Wolfson Institute of Preventive Medicine, Queen Mary University of London, London, United Kingdom; 16 Division of Infectious Diseases, Department of Medicine, Faculty of Medicine and Dentistry, University of Alberta, Edmonton, Alberta, Canada; 17 Department of Medical Microbiology, University of Manitoba, Winnipeg, Manitoba, Canada; 18 Central Laboratory and Vaccination Centre, Stiftung Juliusspital, Academic Teaching Hospital of the University of Wuerzburg, Wuerzburg, Germany; 19 Department of Gynaecology, University Hospital KU Leuven Gasthuisberg, Leuven, Belgium; 20 Network on Cooperative Cancer Research, RTICC, Catalonia, Spain; 21 Department of Gynecology and Obstetrics, Federal University of Paraná, Infectious Diseases in Gynecology and Obstetrics Sector, Curitiba, Parana, Brazil; 22 Department of Obstetrics and Gynaecology, University of the Philippines College of Medicine, Philippine General Hospital, Manila, Philippines; 23 Facharzt für Frauenheilkunde und Geburtshilfe, Hamburg, Germany; 24 Department of Obstetrics and Gynaecology, University of Helsinki, Helsinki, Finland; 25 Clinics, Paris, France; 26 GlaxoSmithKline Vaccines, Wavre, Belgium; 27 GlaxoSmithKline Vaccines, King of Prussia, Pennsylvania, United States of America; University of Ottawa, Canada

## Abstract

**Background:**

The control arm of PATRICIA (PApillomaTRIal against Cancer In young Adults, NCT00122681) was used to investigate the risk of progression from cervical HPV infection to cervical intraepithelial neoplasia (CIN) or clearance of infection, and associated determinants.

**Methods and Findings:**

Women aged 15-25 years were enrolled. A 6-month persistent HPV infection (6MPI) was defined as detection of the same HPV type at two consecutive evaluations over 6 months and clearance as ≥2 type-specific HPV negative samples taken at two consecutive intervals of approximately 6 months following a positive sample. The primary endpoint was CIN grade 2 or greater (CIN2+) associated with the same HPV type as a 6MPI. Secondary endpoints were CIN1+/CIN3+ associated with the same HPV type as a 6MPI; CIN1+/CIN2+/CIN3+ associated with an infection of any duration; and clearance of infection. The analyses included 4825 women with 16,785 infections (3363 womenwith 6902 6MPIs). Risk of developing a CIN1+/CIN2+/CIN3+ associated with same HPV type as a 6MPI varied with HPV type and was significantly higher for oncogenic versus non-oncogenic types. Hazard ratios for development of CIN2+ were 10.44 (95% CI: 6.96-15.65), 9.65 (5.97-15.60), 5.68 (3.50-9.21), 5.38 (2.87-10.06) and 3.87 (2.38-6.30) for HPV-16, HPV-33, HPV-31, HPV-45 and HPV-18, respectively. HPV-16 or HPV-33 6MPIs had ~25-fold higher risk for progression to CIN3+. Previous or concomitant HPV infection or CIN1+ associated with a different HPV type increased risk. Of the different oncogenic HPV types, HPV-16 and HPV-31 infections were least likely to clear.

**Conclusions:**

Cervical infections with oncogenic HPV types increased the risk of CIN2+ and CIN3+. Previous or concomitant infection or CIN1+ also increased the risk. HPV-16 and HPV-33 have by far the highest risk of progression to CIN3+, and HPV-16 and HPV-31 have the lowest chance of clearance.

## Introduction

Persistent infection with oncogenic human papillomavirus (HPV) is necessary for the development of cervical cancer [1]. Approximately 15 oncogenic HPV types have been identified to date, of which HPV-16 and HPV-18 are the most prevalent in cervical cancer, accounting for approximately 70% of cases worldwide [2].

Many sexually active individuals become infected with HPV after beginning their first sexual relationship [3]. HPV infections are usually transient, and even those that persist for a few months are usually cleared naturally. Development of cervical intraepithelial neoplasia (CIN) and cervical cancer is almost always preceded by a persistent oncogenic HPV infection [4,5]. Numerous determinants are thought to affect progression of HPV infection to a CIN, including behavioural determinants such as tobacco exposure, sexual intercourse with increased numbers of partners, contraceptive use and previous pregnancy [6–8], as well as immunosuppression and presence of infections with other sexually-transmitted pathogens such as *Chlamydia trachomatis* and herpes simplex virus [9–11]. CIN is diagnosed from histological analysis of a cervical lesion. It is categorised into three grades: CIN grade 1 (CIN1), grade 2 (CIN2) and grade 3 (CIN3) with CIN3 considered as the immediate precursor to invasive cervical cancer (ICC) [12]. 

A better understanding is needed of the natural history of progression of a cervical HPV infection to a CIN or to clearance of the infection. Such analyses can be performed using the control arm of large trials of prophylactic HPV vaccines where data on HPV types, histological lesions and potential determinants of disease progression are extensively collected. The present paper reports the analysis of HPV infection and its clearance or progression to CIN in the PApilloma Trial against Cancer In young Adults (PATRICIA), a phase III trial of the HPV-16/18 AS04-adjuvanted vaccine (*Cervarix^®^*) in over 18,000 young women.

## Methods

This analysis was based on data obtained from the control arm of the double-blind, randomised, multinational (14 countries), controlled, 4-year PATRICIA trial (NCT00122681/580299/008). The objectives were to investigate the time between detection of an HPV infection (through HPV genotyping) and development of a CIN lesion associated with the same HPV type, and to evaluate determinants associated with disease progression or natural clearance of infection.

### Study population and procedures

The clinical trial methodology, including full inclusion and exclusion criteria, trial locations and dates, has been described previously [13,14]. Briefly, women aged 15–25 years with no more than 6 lifetime sexual partners were enrolled in the study and were randomised to HPV-16/18 AS04-adjuvanted vaccine or control hepatitis A vaccine. HPV genotyping of cervical liquid-based cytology samples was performed at baseline and every 6 months throughout the 4-year study by polymerase chain reaction (PCR). Cytological examination using the Bethesda system was performed every 12 months and, when a biopsy was performed by the physician, histological classification was done. Oncogenic HPV types tested for were HPV-16, 18, 31, 33, 35, 39, 45, 51, 52, 56, 58, 59, 66 and 68; non-oncogenic types tested for were HPV-6, 11, 34, 40, 42, 43, 44, 53, 54, 70 and 74 [15].

Subjects completed a behavioural questionnaire [16] including tobacco exposure, sexual intercourse and contraceptive use at the second study visit, 1 month after the first vaccination, and yearly during the follow-up period. For the purpose of this analysis, the term sexual intercourse included penetrative, non-penetrative genital-to-genital, or oral-genital sexual contact.

Written informed consent or assent was obtained from all participants or their parents, and the protocol and other materials were approved by independent ethics committees or institutional review boards (Table S1 in File S1).

### Endpoint definitions

HPV infections were classified according to their duration as a transient infection, a 6-month persistent infection (6MPI), a 12-month persistent infection (12MPI), a less than 6MPI, and an infection detected only at the last visit of the study. A transient infection was defined as detection of a specific HPV type in a cervical sample at any single point during the follow-up period, followed by a negative sample for the same HPV type at the next evaluation. A 6MPI was defined as detection of the same HPV type at two consecutive evaluations over a 6-month period or greater i.e. a sequence of positive samples with the same HPV type not interrupted by negative samples over a total range of >5 months (>150 days). The start of the 6MPI was defined as the date of the first positive sample in the sequence. A 12MPI was defined in the same way, over a 12-month period. A less than 6MPI was defined as an infection with duration ≤150 days, again with the same HPV type, not interrupted by negative samples (although visits were scheduled for every 6 months, in some cases there was less time between consecutive visits, allowing a category of persistent HPV infection of less than 6 months’ duration).

Clearance was defined as the occurrence of at least two consecutive type-specific negative HPV DNA samples taken at approximately a 6-month interval following a positive sample. Although we recognise that apparent clearance could in reality be an inability to detect the infection, we use the term clearance for convenience. 

Histologically confirmed lesions were detected in colposcopically-directed biopsy samples or post-excision samples following treatment and were categorised as CIN grade 1 or greater (CIN1+), CIN grade 2 or greater (CIN2+) and CIN grade 3 or greater (CIN3+). CIN1+ included CIN1, CIN2, CIN3 and adenocarcinoma in situ (AIS) identified by standard methods. 

### Statistical analysis

The primary endpoint was CIN2+ associated with the same HPV type as a 6MPI. Secondary endpoints were CIN1+ and CIN3+ associated with the same HPV type as a 6MPI; CIN1+, CIN2+ and CIN3+ associated with the same HPV type as an infection of any duration; and clearance of HPV infections.

#### Analysis cohort

**Figure 1:**
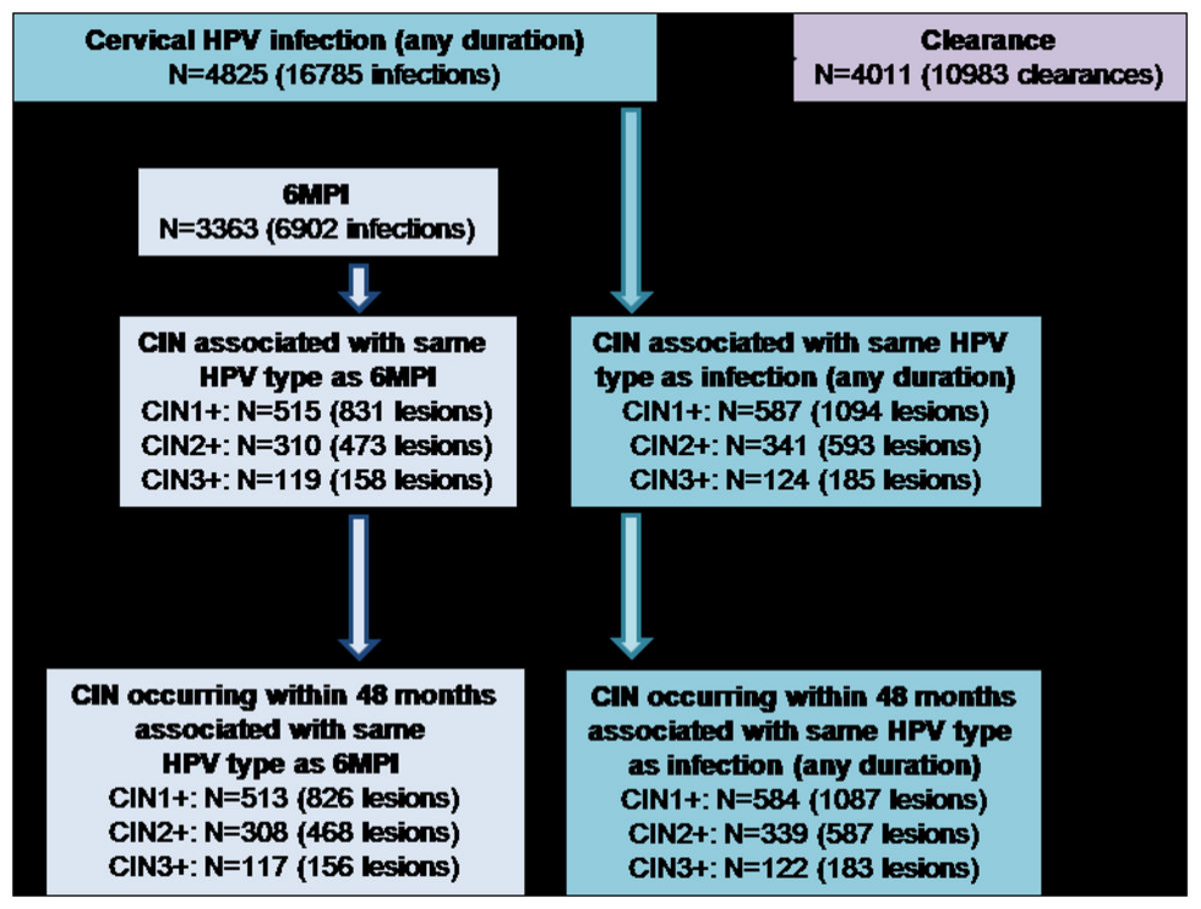
Subject disposition. From the 9337 women enrolled in the control arm of the PATRICIA trial, 4512 were excluded from the present analyses including 4431 subjects with no cervical HPV infection detected during the 48 months of follow-up. 4825 women representing 16785 cervical HPV infections (blue) were included and two sub-cohorts were constituted: women with a 6-month persistent HPV infection (6MPI) (n = 3363 representing 6902 infections) (light blue) and women with a natural clearance of the HPV infection (n = 4011 representing 10983 clearances) (light purple). The numbers of women and of CIN1+/CIN2+/CIN3+ lesions were calculated after exclusion of the lesions occurring after 48 months for any duration of HPV infection (blue) and for 6MPI (light blue). Among the 119 subjects with 6MPI and CIN3+, 9 had AIS (11 episodes). Among the 124 subjects with an infection of any duration and CIN3+, 10 had AIS (13 episodes). No invasive cervical cancer was found. 6MPI: 6-month persistent infection; CIN: cervical intraepithelial neoplasia; HPV: human papillomavirus; TVC-E: total vaccinated cohort for efficacy.

The analysis was performed in the control group of the total vaccinated cohort for efficacy (TVC-E) (Figure 1). The TVC-E included all women who received at least one dose of control vaccine and had normal or low-grade cytology at baseline (ie, negative, atypical squamous cells of undetermined significance or low-grade squamous intraepithelial lesion). The analysis population included only women aged 15–25 years and only those in whom at least one cervical HPV infection was detected during the study. Infections in participants in whom HPV DNA was detected at baseline but not at month 6 were classified as transient for the purpose of the analysis. However, it must be noted that these could have been persistent infections because the date of their origin was unknown. 

#### Determinants

The main determinants of interest were HPV type and duration of HPV infection. Covariates known to influence the risk of acquiring HPV infection were also accounted for in the analyses. Eight potential behavioural characteristics were included in the models: tobacco exposure measured as number of pack-years (one pack-year was equivalent to 365 packs of cigarettes), age at first sexual intercourse, number of sexual partners during the past 12 months, history of *Chlamydia trachomatis* during the past 12 months, marital/partner status, previous pregnancy, use of hormones for contraception or other indication, and use of an intrauterine device. 

In addition, the models accounted for previous cervical HPV infection with an oncogenic HPV type or with only non-oncogenic HPV types (i.e. preceding the onset of the reference infection), cervical HPV co-infection with an oncogenic HPV type or with only non-oncogenic HPV types (i.e. following the onset of the reference infection and preceding detection of the lesion), previous CIN1+ lesion associated with any oncogenic or non-oncogenic HPV type different to the reference infection (i.e. CIN1+ preceding the onset of the reference infection), and concomitant CIN1+ lesion associated with any oncogenic or non-oncogenic HPV type different to the reference infection (i.e. CIN1+ following the onset of the reference infection and preceding its end). Cervical HPV co-infections and CIN1+ lesions associated with an HPV type different to the reference HPV infection were included in the models as time-varying covariates.

#### General statistical considerations

The time between a cervical HPV infection and development of a CIN lesion was analysed using the Kaplan-Meier method as well as univariate and multivariable Cox regression models. The statistical unit was the infection, and variance estimates adjusted for the correlation within subjects were obtained using the robust estimation method [17,18]. The same methods were used to analyse natural clearance of HPV infection. All data were censored at the last recorded visit or at 48 months, whichever occurred first. Covariates with a p-value <0.2 in the univariate model were included in the multivariable model, with the exception of region which was always included regardless of the p-value obtained. Infections or lesions with a missing value for a covariate included in the analysis were excluded from the multivariable analysis. All analyses were performed using SAS version 9.2. 

## Results

### Subject disposition and characteristics

A total of 9256 women in the TVC-E were aged 15–25 years and had negative or low-grade cytology (Figure 1). Of these, subjects with no cervical HPV infection during the study (n=3794, 41.0%) or in whom an infection was detected only at the last visit of the study (n=637, 6.9%) were excluded from the analysis (Figure 1). A total of 4825 women with at least one cervical HPV infection of any duration (16,785 infections) during the 48 month follow-up were included. Of these, 3363 women had at least one 6MPI (6902 infections), 2283 had a 12MPI (3487 infections), 355 had persistent HPV infection(s) of less than 6 months (449 infections), and 3945 had transient HPV infection(s) (9434 infections). A total of 1285 women had an infection detected at baseline but not at month 6 (classed as a transient infection for the purpose of the analysis, although its duration prior to study entry was unknown). The five most prevalent HPV types were HPV-16 (9.3% of any infections and 13.9% of 6MPIs), HPV-18 (5.6% and 6.0%), HPV-31 (5.4% and 6.5%), HPV-33 (2.9% and 3.0%) and HPV-45 (2.3% and 2.2%). The prevalence of other oncogenic HPV types was 45.7% (any infection) and 47.4% (6MPI), and the prevalence of non-oncogenic types was 28.7% (any infection) and 20.9% (6MPI).

Among the subjects with at least one cervical HPV infection of any duration, mean (SD) age was 19.7 (3.1) years, most were single (74.4%), with no tobacco exposure (63.4%), had started sexual intercourse between the ages of 15 and 17 years (51.8%), had one or no sexual partner during the past 12 months (71.3%), had not been pregnant (73.1%), had no history of *Chlamydia trachomatis* (90.3%) and had used hormonal contraception (65.4%) (Table 1). Characteristics of subjects in whom at least one 6MPI was detected were similar.

**Table 1:** Frequency distribution of age, region and other determinants at baseline according to duration of cervical HPV infection.

**Determinant**	**Category**	**6MPI**	**HPV infection (any duration)**
		**N=3363**	**N=4825**
Age (years)	Mean (SD)	19.5 (3.0)	19.7 (3.1)
	Median (range)	19 (15–25)	19 (15–25)
Age group, n (%)	15-17 years	1322 (39.3)	1753 (36.3)
	18-25 years	2041 (60.7)	3072 (63.7)
Region, n (%)	Europe	1422 (42.3)	1874 (38.8)
	Asia Pacific	767 (22.8)	1229 (25.5)
	North America	568 (16.9)	857 (17.8)
	Latin America	606 (18.0)	865 (17.9)
Marital/partner status, n (%)	Single	2595 (77.2)	3591 (74.4)
	Living or lived with partner**^*2*^**	733 (21.8)	1178 (24.4)
	Missing	35 (1.0)	56 (1.2)
Tobacco exposure (number of pack-years), n (%)	None or less than 6 months (<0.5)	2031 (60.4)	3058 (63.4)
	At least 6 months (≥0.5)	1296 (38.5)	1713 (35.5)
	Missing	36 (1.1)	54 (1.1)
Age at first sexual intercourse, n (%)	Never had sexual intercourse	3 (0.1)	26 (0.5)
	≥26	3 (0.1)	6 (0.1)
	18-25	996 (29.6)	1525 (31.6)
	15-17 years	1790 (53.2)	2497 (51.8)
	<15 years	569 (16.9)	763 (15.8)
	Missing	2 (0.1)	8 (0.2)
Number of sexual partners during the past 12 months, n (%)	0–1	2294 (68.2)	3441 (71.3)
	2-3	869 (25.8)	1132 (23.5)
	≥4	193 (5.7)	238 (4.9)
	Missing	7 (0.2)	14 (0.3)
Previous pregnancy, n (%)	Yes	817 (24.3)	1281 (26.6)
	No	2539 (75.5)	3527 (73.1)
	Missing	7 (0.2)	17 (0.4)
At least one delivery, n (%)	Yes	516 (15.3)	814 (16.9)
	No	2837 (84.4)	3991 (82.7)
	Missing	10 (0.3)	20 (0.4)
History of *Chlamydia trachomatis* during the past 12 months, n (%)	Yes	344 (10.2)	468 (9.7)
	No	3018 (89.7)	4355 (90.3)
	Missing	1 (0.03)	2 (0.04)
Contraceptive use [1], n (%)	Abstinent or no contraception	1032 (30.7)	1535 (31.8)
	Hormonal	2253 (67.0)	3155 (65.4)
	Intra-uterine device	146 (4.3)	221 (4.6)
	Sterilised	25 (0.7)	38 (0.8)
History of sexual intercourse at study entry	Yes	3114 (92.6)	4426 (91.7)
	No	242 (7.2)	385 (8.0)
	Missing	7 (0.2)	14 (0.3)
History of sexual intercourse at the end of the study	Yes	3359 (99.9)	4798 (99.4)
	No	3 (0.1)	26 (0.5)
	Missing	1 (0.03)	1 (0.02)

^1^ Women could be using more than one method of contraception.

^2^ Living with or had lived with partner included married, living with partner, widowed, divorced, separated.

6MPI: 6-month persistent infection; HPV: human papillomavirus

### Risk of Developing a CIN Lesion Associated with a 6-Month Persistent Infection

**Figure 2:**
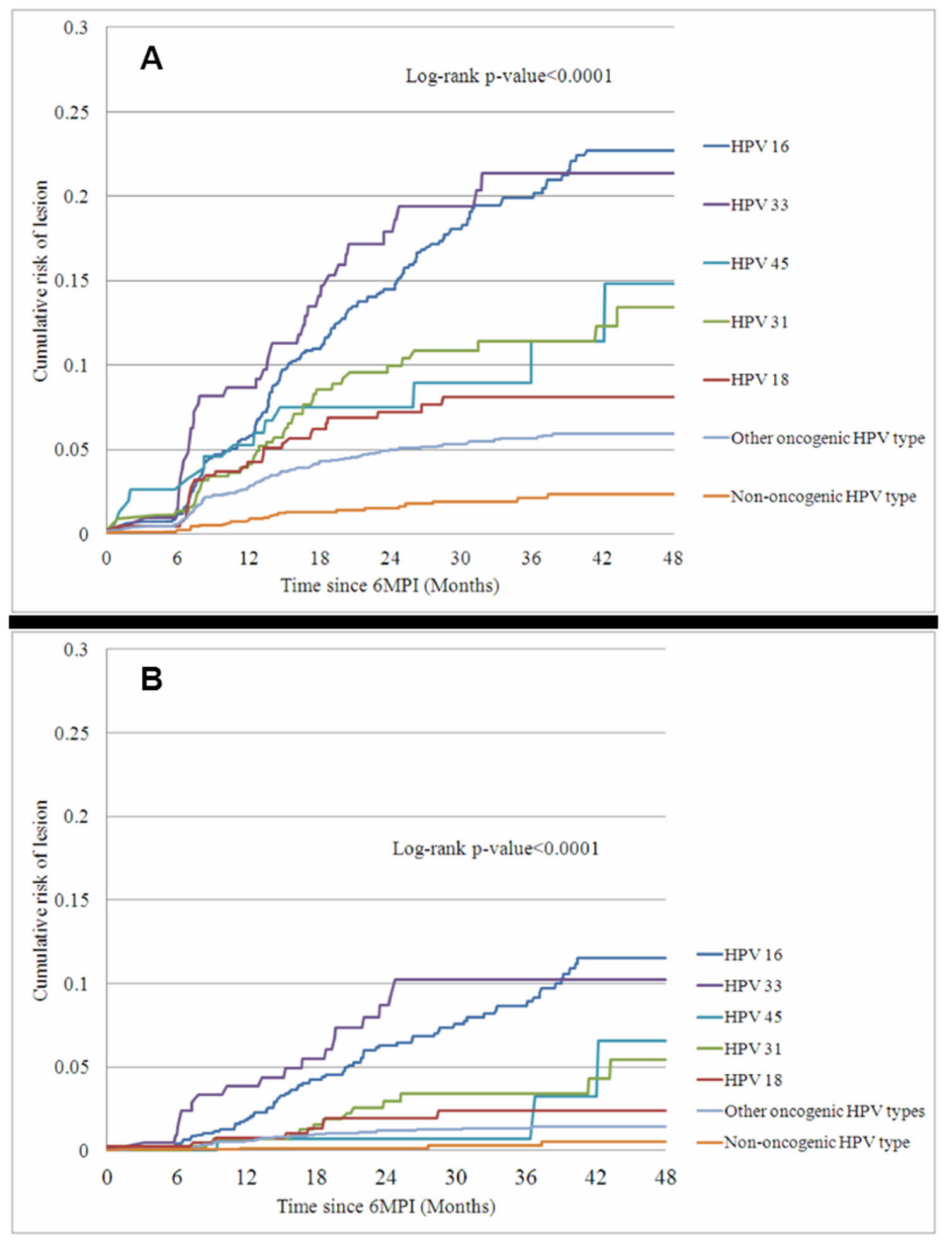
Risk of progression of a 6MPI to CIN2+ or CIN3+ associated with the same HPV type. **2a. CIN2+** **2b. CIN3+** Kaplan–Meier Estimates of Cumulative Risk (%) of Developing CIN2+ Lesions (Figure 2a) or CIN3+ Lesions (Figure 2b) Associated with the Same HPV Type Were Calculated for HPV-16, HPV-18, HPV-31, HPV-33, HPV-45, Other Oncogenic HPV Types and Non-Oncogenic HPV Types

The number of women with a 6MPI who developed a CIN1+, CIN2+ or CIN3+ lesion during the study is shown in Figure 1. Lesions that developed after Month 48 were not included in the analysis. The multivariable analysis showed that, for women with a 6MPI, the cumulative risk of developing a CIN1+, CIN2+ or CIN3+ lesion associated with the same HPV type significantly increased for an infection with the five most prevalent HPV types (HPV-16/18/31/33/45) or with any other oncogenic type compared with a non-oncogenic HPV type (Table 2; Figures 2a and 2b). Compared with women with a non-oncogenic HPV 6MPI, women with HPV-16 or HPV-33 6MPIs had approximately a 25-fold higher risk for progression to a CIN3+ lesion, those with a HPV-31 6MPI had a 10-fold higher risk, those with HPV-18 and HPV-45 6MPIs had a 6-fold higher risk, and those with 6MPIs with any other oncogenic type had a 4-fold higher risk. The association between a 6MPI with an oncogenic HPV type and development of a lesion became stronger as lesion severity increased; for example, the Hazard Ratio (HR) from the multivariable analysis for HPV-16 increased from 4.60 for CIN1+ to 10.44 for CIN2+ and to 26.82 for CIN3+. 

**Table 2:** Multivariable analysis of the risk of progression of a 6MPI infection to a CIN lesion associated with the same HPV type.

	**CIN1+**			**CIN2+**			**CIN3+**		
	**Hazard ratio^*1*^ (95% CI)**	**p-value**		**Hazard ratio^*1*^ (95% CI)**	**p-value**		**Hazard ratio^*1*^ (95% CI)**	**p-value**	
	**6835 infections in 3337 women**			**6835 infections in 3337 women**			**6785 infections in 3325 women**		
	**818 lesions^*2*^**			**464 lesions^*2*^**			**152 lesions^*2*^**		
**HPV type**									
Non-oncogenic type	1		-	1		-	1		-
HPV-16	4.60 (3.47–6.09)		<0.0001	10.44 (6.96–15.65)		<0.0001	26.82 (10.00–71.94)		<0.0001
HPV-18	2.43 (1.70–3.48)		<0.0001	3.87 (2.38–6.30)		<0.0001	6.04 (1.82–20.04)		0.0033
HPV-31	3.33 (2.39–4.64)		<0.0001	5.68 (3.50–9.21)		<0.0001	9.80 (3.16–30.37)		<0.0001
HPV-33	4.44 (3.12–6.32)		<0.0001	9.65 (5.97–15.60)		<0.0001	25.04 (9.00–69.69)		<0.0001
HPV-45	2.53 (1.53–4.17)		0.0003	5.38 (2.87–10.06)		<0.0001	6.88 (1.54–30.74)		0.0116
Other oncogenic type	2.16 (1.66–2.81)		<0.0001	2.80 (1.87–4.19)		<0.0001	3.73 (1.34–10.37)		0.0117
	*<0.0001*			*<0.0001*			*<0.0001*		
**Previous cervical HPV infection^*3*^**									
No	1		-	Not included			Not included		
Yes (at least 1 oncogenic HPV type)	1.32 (1.11–1.56)		0.0016						
Yes (only non-oncogenic HPV types)	1.15 (0.78–1.70)		0.4846						
	*0.0071*								
**Cervical HPV co-infection*^4^*,*^5^***									
No	1		-	1		-	1		-
Yes (at least 1 oncogenic HPV type)	1.75 (1.44–2.12)		<0.0001	1.88 (1.46–2.41)		<0.0001	1.36 (0.94–1.98)		0.1018
Yes (only non-oncogenic HPV types)	1.05 (0.76–1.46)		0.7689	0.80 (0.49–1.31)		0.3731	0.74 (0.33–1.66)		0.4657
	*<0.0001*			*<0.0001*			*0.0955*		
**Previous CIN1+^*6*^**									
No	1		-	1		-	1		-
Yes (at least 1 oncogenic HPV type)	1.94 (1.21–3.12)		0.0059	2.75 (1.58–4.80)		0.0004	3.78 (1.69–8.41)		0.0012
Yes (only non-oncogenic HPV types)	-			-		-	-		-
	*0.0059*			*0.0004*			*0.0012*		
**Concomitant CIN1**+***^5^***,***^7^***									
No	1		-	1		-	1		-
Yes (any oncogenic or non-oncogenic HPV type)	2.27 (1.67–3.08)		<0.0001	2.03 (1.40–2.93)		0.0002	3.86 (2.31–6.47)		<0.0001
	*<0.0001*			*0.0002*			*<0.0001*		

^1^ Covariates were included in the multivariable analysis if they had a global p-value of <0.2 in the univariate analysis (except region which was always included); covariates were: region, smoking, age at first sexual intercourse, number of sexual partners during the past 12 months, history of Chlamydia trachomatis during the past 12 months, marital/partner status, previous pregnancy, use of hormones for contraception or other indication, use of intrauterine device, previous or concomitant HPV co-infections, previous or concomitant CIN1+. Refer to Tables S2–S4 for more detail.

^2^ Infections or lesions with a missing value for a covariate included in the analysis were excluded from the multivariable analysis.

^3^ Preceding the onset of the reference infection.

^4^ Following the onset of the reference infection and preceding detection of the lesion.

^5^ Time-varying covariate.

^6^ CIN1+ associated with an HPV type different to the reference infection, preceding the onset of the 6MPI.

^7^ CIN1+ associated with an HPV type different to the reference infection, concomitant to the 6MPI (following its onset and preceding its end).

Values in italics show the global p-value.

6MPI: 6-month persistent infection; CIN: cervical intraepithelial neoplasia; HPV: human papillomavirus

A previous infection with an oncogenic HPV type different from the 6MPI significantly increased the risk of CIN1+ (previous infection was not included in the multivariable analysis for CIN2+ and CIN3+ because a p-value of >0.2 was obtained in the univariate analysis) (Table 2). Presence of a co-infection with a different oncogenic HPV type also significantly increased the risk of developing CIN1+ or CIN2+, but not CIN3+ (Table 2). A CIN1+ lesion associated with a different oncogenic HPV type also significantly increased the risk of CIN1+, CIN2+ or CIN3+, regardless of whether it occurred prior to or concomitant with the 6MPI. 

Results of the univariate and multivariable analyses of the risk of developing a CIN lesion that account for behavioural determinants are shown in Tables S2–S4 in File S1. Behavioural determinants that were significant (p<0.05) in the multivariable analysis were tobacco exposure and previous pregnancy for CIN1+, previous pregnancy and use of hormones for contraception or other indication for CIN2+, and previous pregnancy and age at first sexual intercourse for CIN3+. 

### Risk of developing a CIN lesion associated with an HPV infection of any duration

HPV-16, HPV-51 and HPV-52 were the most frequent HPV types observed (1557, 1613 and 1374 infections, respectively), with HPV-16 being the most frequently detected in CIN1+ and CIN2+. As observed for 6MPIs, in the multivariable analysis, the association between an infection of any duration with an oncogenic HPV type and development of a lesion became stronger as lesion severity increased (Table 3). Again, the risk of developing a CIN lesion associated with the same HPV type was higher for HPV-16, HPV-33, HPV-31, HPV-18 and HPV-45, any other oncogenic HPV type compared with non-oncogenic HPV types; the highest risk was for HPV-16 and HPV-33 (approximately 20-fold), followed by HPV-31 (8-fold), and then by HPV-18 or HPV-45 (4.5-fold) (Table 3).

**Table 3:** Multivariable analysis of the risk of progression of an HPV infection of any duration to a CIN lesion associated with the same HPV type.

	**CIN1+**		**CIN2+**		**CIN3+**	
	**Hazard ratio^*1*^ (95% CI)**	**p-value**	**Hazard ratio^*1*^ (95% CI)**	**p-value**	**Hazard ratio^*1*^ (95% CI)**	**p-value**
	**16580 infections in 4789 Women**		**16580 infections in 4789 women**		**16596 infections in 4791 women**	
	**1069 lesions^*2*^**		**577 lesions^*2*^**		**180 lesions^*2*^**	
**HPV type**						
Non-oncogenic type	1	-	1	-	1	-
HPV-16	4.39 (3.52–5.47)	<0.0001	9.25 (6.84–12.51)	<0.0001	20.93 (9.97–43.95)	<0.0001
HPV-18	2.47 (1.85–3.30)	<0.0001	3.56 (2.40–5.27)	<0.0001	4.74 (1.78–12.58)	0.0018
HPV-31	3.34 (2.57–4.34)	<0.0001	5.09 (3.56–7.29)	<0.0001	7.82 (3.46–17.63)	<0.0001
HPV-33	4.36 (3.29–5.79)	<0.0001	9.14 (6.34–13.18)	<0.0001	20.47 (9.45–44.35)	<0.0001
HPV-45	2.28 (1.49–3.49)	0.0002	3.64 (2.08–6.40)	<0.0001	4.45 (1.17–16.97)	0.0286
Other oncogenic type	2.19 (1.79–2.67)	<0.0001	2.63 (1.94–3.57)	<0.0001	3.51 (1.62–7.59)	0.0014
	*<0.0001*		*<0.0001*		*<0.0001*	
**Duration of infection**						
Transient	1	-	1	-	1	-
Less than 6MPI	2.48 (1.54–3.98)	0.0002	2.32 (1.22–4.40)	0.0102	1.45 (0.32–6.48)	0.6275
6MPI	4.25 (3.63–4.97)	<0.0001	4.61 (3.66–5.81)	<0.0001	5.29 (3.34–8.38)	<0.0001
	*<0.0001*		*<0.0001*		*<0.0001*	
**Previous cervical HPV infection^*3*^**						
No	1	-	1	-	Not included	
Yes (at least 1 oncogenic HPV type)	1.52 (1.31–1.77)	<0.0001	1.27 (1.03–1.57)	0.0254		
Yes (only non-oncogenic HPV types)	1.22 (0.85–1.73)	0.2791	1.21 (0.75–1.96)	0.4324		
	*<0.0001*		*0.0803*			
**Cervical HPV co-infections*^4^*,*^5^***						
No	1	-	1	-	1	-
Yes (at least 1 oncogenic HPV type)	1.85 (1.54–2.21)	<0.0001	1.93 (1.52–2.45)	<0.0001	1.40 (0.99–1.99)	0.0595
Yes (only non-oncogenic HPV types)	0.99 (0.73–1.34)	0.9366	0.76 (0.48–1.20)	0.2375	0.60 (0.27–1.33)	0.2110
	*<0.0001*		*<0.0001*		*0.0243*	
**Previous CIN1+ ^*6*^**						
No	1	-	1	-	1	-
Yes (any oncogenic or non-oncogenic HPV type)	2.32 (1.59–3.40)	<0.0001	2.74 (1.73–4.32)	<0.0001	3.65 (1.76–7.54)	0.0005
	*<0.0001*		*<0.0001*		*0.0005*	
**Concomitant CIN1**+***^5^***,***^7^***						
No	1	-	1	-	1	-
Yes (any oncogenic or non-oncogenic HPV type)	2.76 (2.10–3.63)	<0.0001	2.65 (1.89–3.71)	<0.0001	4.70 (2.77–7.99)	<0.0001
	*<0.0001*		*<0.0001*		*<0.0001*	

^1^ Covariates were included in the multivariable analysis if they had a global p-value of <0.2 in the univariate analysis (except region which was always included); covariates were: region, smoking, age at first sexual intercourse, number of sexual partners during the past 12 months, history of Chlamydia trachomatis during the past 12 months, marital/partner status, previous pregnancy, use of hormones for contraception or other indication, use of intrauterine device, previous or concomitant HPV co-infections, previous or concomitant CIN1+. Refer to Tables S5–S7 for more detail.

^2^ Infections or lesions with a missing value for a covariate included in the analysis were excluded from the multivariable analysis.

^3^ Preceding the onset of the reference infection.

^4^ Following the onset of the reference infection and preceding detection of the lesion.

^5^ Time-varying covariate.

^6^ CIN1+ associated with an HPV type different to the reference infection, preceding the onset of the 6MPI.

^7^ CIN1+ associated with an HPV type different to the reference, concomitant to the 6MPI (following its onset and preceding its end).

Values in italics show the global p-value.

6MPI: 6-month persistent infection; CIN: cervical intraepithelial neoplasia; HPV: human papillomavirus

The duration of the HPV infection had a marked impact on the risk of developing a lesion, with a 6MPI showing the highest risk for CIN lesion development, followed by a persistent infection of less than 6 months duration, while a transient HPV infection showed the lowest risk (Table 3). Compared with a transient infection, both persistent infections of less than 6 months duration and 6MPIs were significantly associated with higher risk of CIN1+ (HR: 2.48 and 4.25, respectively) and CIN2+ (HR: 2.32 and 4.61, respectively); only 6MPI was significantly associated with increased risk of CIN3+ (HR: 5.29). 

Co-infection with an oncogenic HPV type different from the reference infection significantly increased the risk of developing CIN1+, CIN2+ or CIN3+ (Table 3). A previous infection with a different oncogenic HPV type also increased the risk of CIN1+ and CIN2+ (the analysis was not performed for CIN3+ because this covariate was not statistically significant (p>0.2) in the univariate model). A previous or concomitant CIN1+ lesion associated with a different oncogenic HPV type also significantly increased the risk of lesion development.

Results of the univariate and multivariable analyses of the risk of developing a CIN lesion that account for behavioural determinants are shown in Tables S5–S7 in File S1. Behavioural determinants that were significant (p<0.05) in the multivariable analysis were tobacco exposure and previous pregnancy for CIN1+, previous pregnancy and use of hormones for contraception or other indication for CIN2+, and previous pregnancy for CIN3+.

### Clearance of HPV infection

**Figure 3:**
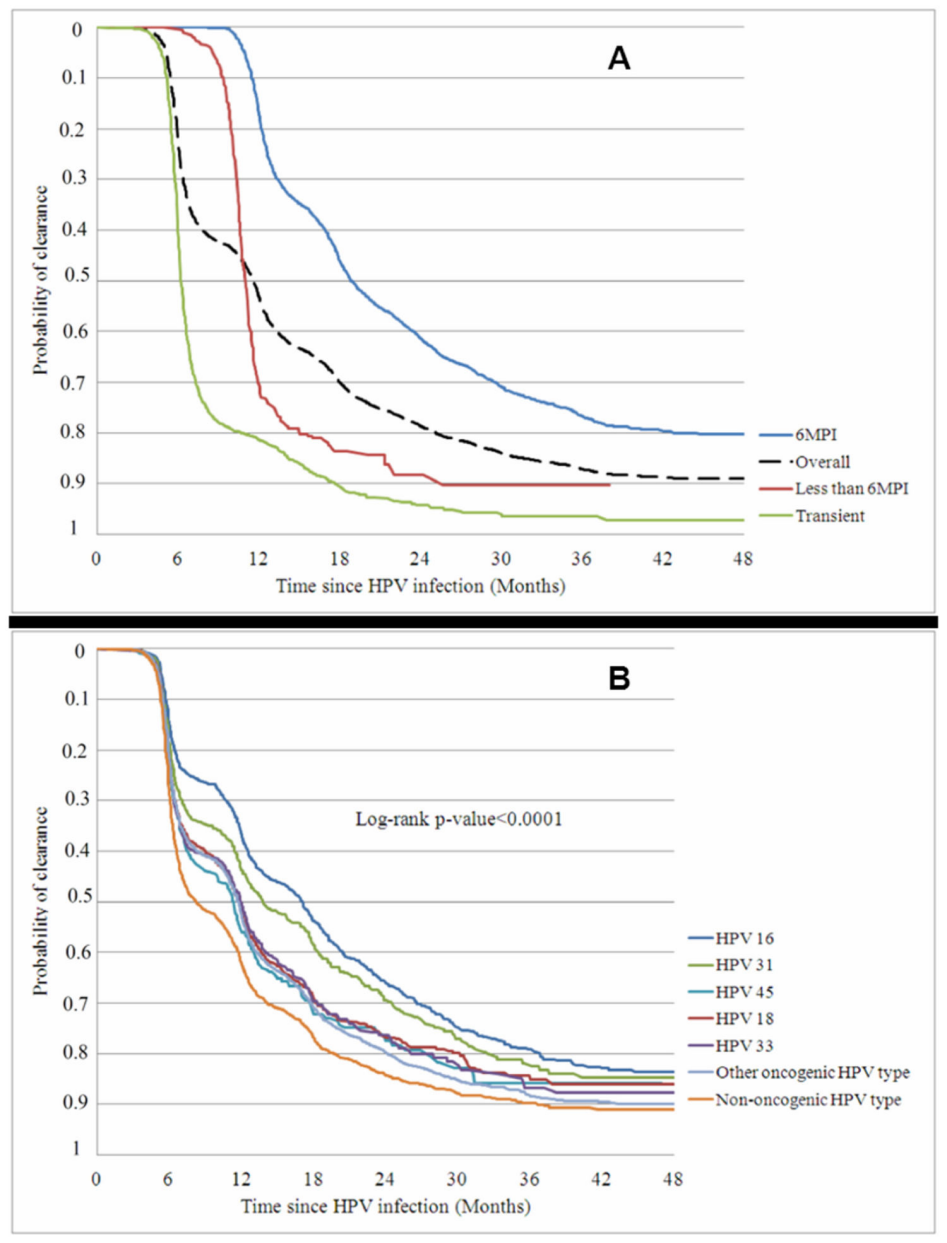
Cumulative chance of clearing a cervical HPV infection (prevalent and incident infections). **3a. Duration of infection** **3b. HPV type (infection of any duration)** Kaplan–Meier estimates of probability of clearance (%) were calculated for transient, less than 6-month persistent HPV infection (6MPI), 6MPI and overall (Figure 3a) or according the HPV types (Figure 3b): HPV-16, HPV-18, HPV-31, HPV-33, HPV-45, other oncogenic HPV types and non-oncogenic HPV types.

Overall, 53%, 79%, 87% and 89% of all HPV infections were cleared at 12, 24, 36 and 48 months, respectively (Figure 3a). The median time to clearance for transient HPV infections and 6MPIs was 6.26 months and 18.85 months, respectively (Table 4). HPV-16 and HPV-31 were significantly less likely to clear than a non-oncogenic HPV type, and other oncogenic HPV types had an intermediate chance of clearance (Figure 3b; Table 4). Previous infection with the same or with other HPV types reduced the chance of clearance; in contrast, the chance of clearance was increased by co-infection with another oncogenic HPV type in this analysis.

**Table 4:** Multivariable analysis of the median time to clearance of cervical HPV infections: influence of HPV type, duration of infection and previous and concomitant infection.

	**Median time to clearance [1], months (inter-quartiles)**	**Multivariable analysis of the chance of clearance^*2*^**	
		**Hazard ratio**	**p-value**
		**(95% CI)**	
	**16505 infections in 4793 women^*3*^**		
	**10810 clearances**		
**HPV type**			
Non-oncogenic type	8.26 (5.97–17.57)	1	-
HPV-16	17.11 (7.80–30.26)	0.81 (0.75–0.88)	<0.0001
HPV-18	11.84 (6.20–23.11)	0.93 (0.85–1.03)	0.1748
HPV-31	13.80 (6.43–28.89)	0.82 (0.74–0.90)	<0.0001
HPV-33	12.00 (6.20–21.90)	0.95 (0.84–1.08)	0.4557
HPV-45	11.48 (6.20–23.31)	0.93 (0.80–1.08)	0.3465
Other oncogenic type	11.77 (6.20–20.03)	0.96 (0.91–1.02)	0.1739
	*<0.0001*	*<0.0001*	
**Duration of infection**			
Transient	6.26 (5.70–8.16)	1	-
Less than 6MPI	11.02 (10.16–13.18)	0.40 (0.34–0.45)	<0.0001
6MPI	18.85 (12.66–34.52)	0.14 (0.13–0.14)	<0.0001
	*<0.0001*	*<0.0001*	
**Previous cervical HPV infection^*4*^**			
No	11.25 (6.03–18.79)	1	-
Yes (same HPV type)	13.64 (6.43-NE)	0.58 (0.52–0.64)	<0.0001
Yes (other HPV type[s])	11.84 (6.23–22.43)	0.72 (0.68–0.77)	<0.0001
	*<0.0001*	*<0.0001*	
**Cervical HPV co-infection*^5^*,*^6^***			
No	-	1	-
Yes (at least 1 oncogenic HPV type)	-	1.08 (1.01–1.14)	0.0146
Yes (only non-oncogenic HPV types)	-	1.03 (0.93–1.15)	0.5501
	*Not done**^4^***	*0.0473*	

^1^ Time from first detection to first negative results

^2^ Covariates were included in the multivariable analysis if they had a global p-value of <0.2 (except region which was always included); covariates were: region, smoking, age at first sexual intercourse, number of sexual partners during the past 12 months, history of Chlamydia trachomatis during the past 12 months, marital/partner status, previous pregnancy, use of hormones for contraception or other indication, use of intrauterine device, previous or concomitant HPV co-infections, previous or concomitant CIN1+. Refer to Table S8 for more detail.

^3^ Infections or clearances with a missing value for a covariate included in the analysis were excluded from the multivariable analysis.

^4^ Preceding the onset of the reference infection.

^5^ Following the onset of the reference infection and preceding its end.

^6^ Time-varying covariates: Kaplan-Meier analysis not done.

Values in italics show the log-rank p-value (for the median time to clearance) or the global p-value (for the multivariable analysis).

6MPI: 6-month persistent infection; HPV: human papillomavirus; NE: not estimated

Results of the univariate and multivariable analyses of clearance of HPV infection that account for behavioural determinants are shown in Table S8 in File S1. Determinants that were significant (p<0.05) in the multivariable analysis were region and marital status (the chance of clearance was lower in women who were living or had lived with a partner than in single women).

**Figure 4:**
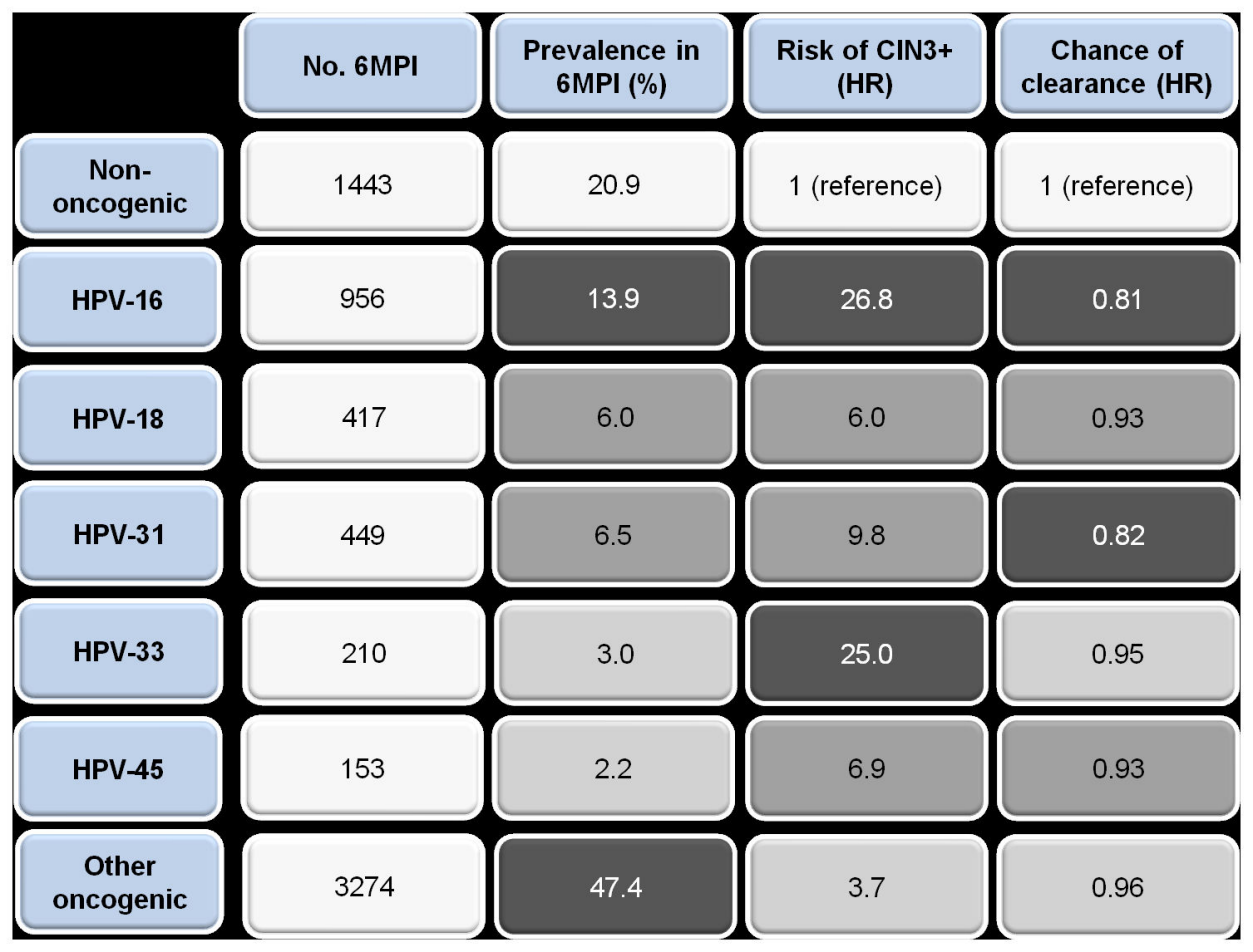
Summary according to HPV type of prevalence in 6MPI, risk of progression to CIN3+ lesion and chance of clearance. Dark-shaded boxes: high prevalence (>10%), high risk of progression (HR >20) or low chance of clearance (HR <0.85) Medium-shaded boxes: medium prevalence (5–10%), medium risk of progression (HR 5–20) or medium chance of clearance (0.85-<0.95) Light-shaded boxes: low prevalence (<5%), low risk of progression (HR <5) or high chance of clearance (HR ≥0.95) Definitions of high, medium and low prevalence, risk of progression and chance of clearance are arbitrary and are based on the results of the present study. 6MPI: 6-month persistent infection; CIN: cervical intraepithelial neoplasia; HPV: human papillomavirus; HR: hazard ratio.

The results for prevalence, risk of progression to CIN and chance of clearance are summarised according to HPV type in Figure 4. HPV-16 has a high prevalence, high risk of progression and low chance of clearance, and is thus overall the most high-risk HPV type. HPV-33 shows a lower prevalence, a high risk of progression and a medium chance of clearance. HPV-31 has a medium prevalence, a medium risk of progression and a low chance of clearance. HPV-18 has a consistently medium prevalence, progression and clearance. Findings for the other HPV types are less consistent.

## Discussion

The analysis evaluated the risk of progression to CIN lesion and the chance of clearance according to HPV type. The role of HPV types and persistent infection as the main risk factors for CIN lesion were confirmed and the results also showed the roles of previous infection and co-infection with an oncogenic HPV type, as well as previous or concomitant CIN1+, as risk factors for progression to CIN in young women. Our large sample size allowed for the first time calculation of the risk of progression of HPV infection to CIN3+. Previous studies have been limited in their sample size and number of infections evaluated, and largely focused on development of invasive cervical cancer in adult women.

The five most prevalent HPV types were HPV-16, HPV-31, HPV-18, HPV-33 and HPV-45. The risk of developing a CIN lesion was higher following infection with oncogenic HPV types than with non-oncogenic types, the highest risk being with HPV-16 and HPV-33 (up to 10-fold higher for CIN2+ and up to 25-fold higher for CIN3+ compared with non-oncogenic types), followed by HPV-31, HPV-45 and HPV-18. The association between an infection with an oncogenic HPV type and the risk of CIN became stronger as lesion severity increased, reflecting the increasing prevalence of HPV types belonging to the A7 and A9 HPV species as lesion severity increases (the A7 species includes HPV-18 and HPV-45, while the A9 species includes HPV-16, HPV-31 and HPV-33).

Our findings reflect those of other studies evaluating prevalence of different HPV types in cervical cancer and the risk of progression from infection to pre-cancerous lesions or cancer. A US-based case-control study showed that the most prevalent HPV types in ICC and carcinoma in situ are HPV-16/18/45 and HPV-16/31/33, respectively [19]. An international cross-sectional study of HPV genotype attribution confirmed that the most prevalent oncogenic HPV types in ICC are HPV-16/18/31/33/35/45/52/58 [2]. Schiffman and colleagues have hypothesised that infections with HPV-16 are most likely to persist and progress to pre-cancerous and cancerous lesions [20]. A population-based screening programme has shown that the risk of progression to CIN2+ is similar for women infected by HPV-16 and HPV-33 [21]. Similarly, more recent data from the control arm of the FUTURE I study reported persistence of 12 months or more and the highest risk of progression to CIN2+ for HPV-16 and HPV-33 [22]. 

HPV-18 is the second most prevalent HPV type in ICC after HPV-16 [2,19]. The recent HERACLES/SCALE studies have suggested that high grade pre-cancerous lesions associated with HPV-18 are more likely to progress to cancer than lesions associated with any other HPV type [23], and a Markov model has also shown a higher risk of progression to later stages for HPV-18 [24]. Our results also suggest that the carcinogenicity of HPV-18 results from high rates of progression of HPV-18-associated CIN3 to ICC, rather than from progression of HPV-18 infection to pre-cancerous lesions.

The risk of developing a CIN was increased by co-infection. Similarly, risk was increased by the presence of a separate CIN1+; this was true regardless of whether the CIN1+ lesion preceded the onset of the reference infection or whether it was concomitant with the reference infection. Some studies have shown that there is a higher chance of acquiring a new HPV type if already infected [25–27], and one study has shown that HPV-16 viral loads in lesions co-infected with other HPV types were higher than in lesions infected with HPV-16 alone [28]. However, other studies have shown that infections with different oncogenic HPV types occur independently of one another [29–31].

Consistent with the risk of progression to CIN, HPV-16 and HPV-31 had the least chance of being cleared, followed by HPV-33, HPV-18, HPV-45 and any other oncogenic type. HPV-16 and HPV-31 have been previously reported to have the lowest rates of clearance [32]. Previous infection diminished the chance of clearance but, unexpectedly, co-infection seemed to increase the chance. However, the observed beneficial effect of co-infection was relatively weak compared with the deleterious effect of previous infection. It is paradoxical that co-infection increased the risk of progression to a CIN but also increased the chance of clearance. There is no clear explanation for these findings. 

Several behavioural determinants were associated with higher risk of progression from infection to lesion (tobacco exposure, previous pregnancy, use of hormones and age at first sexual intercourse) and lower chance of clearance of infection (marital/partner status). An increased risk of cervical abnormalities and/or cancer associated with these determinants has been shown in other studies [6–8].

The analyses had some strengths and limitations. We included only women with a confirmed HPV infection including 6MPI. The analysis therefore focused on factors that may influence the risk of progression from infection to lesion, and was not confounded by the risk of HPV acquisition. Another major strength was that the PATRICIA study included a large population with well-characterised virological and histological samples and high follow-up rates over 48 months. Although the study was multinational, involving women from many ethnic groups, all participants followed the same protocol. The high number of participants and length of follow-up allowed analysis of CIN3+ lesions, which are a better predictor of ICC than CIN2+ lesions [33]. Previous studies evaluating risk of progression have had a smaller sample size and studied a lower number of infections.

A limitation of the analysis is that misclassification of HPV infection below the threshold for detection by PCR (a false-negative result) might have overestimated rates of transient HPV infection and underestimated 6MPI. Therefore, analyses of clearance were done on subjects with two consecutive negative PCR results (“confirmed clearance”). However, a sensitivity analysis using a single negative PCR result gave very similar results (data not shown), demonstrating that the risk of misclassification is low. As mentioned earlier, apparent clearance could actually represent inability to detect the infection. In addition, because pre-cancerous lesions may develop over an extended period, the duration of which is influenced by the HPV type the lesion is associated with, a follow-up of 48 months will not allow detection of all lesions. This may have led to underestimation of the progression rate of some HPV types. A further limitation is that many women started sexual activity before study enrolment and thus some previous HPV infections would not have been recorded. Finally, the study included only women aged 15–25 years, with six or fewer lifetime sexual partners and no history of immunosuppressive disease, limiting its generalizability; in particular, age may have an important influence on the rate of progression or clearance of HPV infection.

In conclusion, we have used data from the control arm of the large, well-controlled PATRICIA study to evaluate the natural history of progression of HPV infection to CIN lesion or to clearance according to individual HPV type. Importantly, we included CIN3, the immediate precursor lesion of cervical cancer. A better understanding of the natural mechanisms of progression to CIN lesions could contribute towards effective implementation of HPV testing as part of screening for early detection and prevention of cervical cancer, as recommended by recent US guidelines [34]. Infection with oncogenic HPV types increased the risk of development of CIN1+, CIN2+ or CIN3+, the highest risk being associated with HPV-16 and HPV-33, whilst infections with HPV types 16 and 31 had the lowest likelihood of being cleared. The risk of progression was increased as lesion severity increased, almost doubling from CIN1+ to CIN2+ and from CIN2+ to CIN3+ for HPV types 16, 31, 33 and 45. We found that previous or concomitant HPV infection or CIN1+ also increased the risk of progression to a lesion, suggesting that multiple HPV infections could influence progression. This finding is relevant to prevention of cervical cancer, and deserves further investigation.

## Supporting Information

File S1 **Supporting Files** Table S1. List of Independent Ethics Committees/ Institutional Review Boards.
Table S2. Risk of progression of a 6-month persistent cervical HPV infection to a CIN1+ lesion associated with the same HPV type.
Table S3. Risk of progression of a 6-month persistent cervical HPV infection to a CIN2+ lesion associated with the same HPV type.
Table S4. Risk of progression of a 6-month persistent cervical HPV infection to a CIN3+ lesion associated with the same HPV type.
Table S5. Risk of progression of an HPV infection of any duration to a CIN1+ lesion associated with the same HPV type.
Table S6. Risk of progression of an HPV infection of any duration to a CIN2+ lesion associated with the same HPV type.
Table S7. Risk of progression of an HPV infection of any duration to a CIN3+ lesion associated with the same HPV type.
Table S8. Clearance and median duration of a cervical HPV infection.
(DOCX)

File S2 **Protocol and STROBE documents.**
(ZIP)
