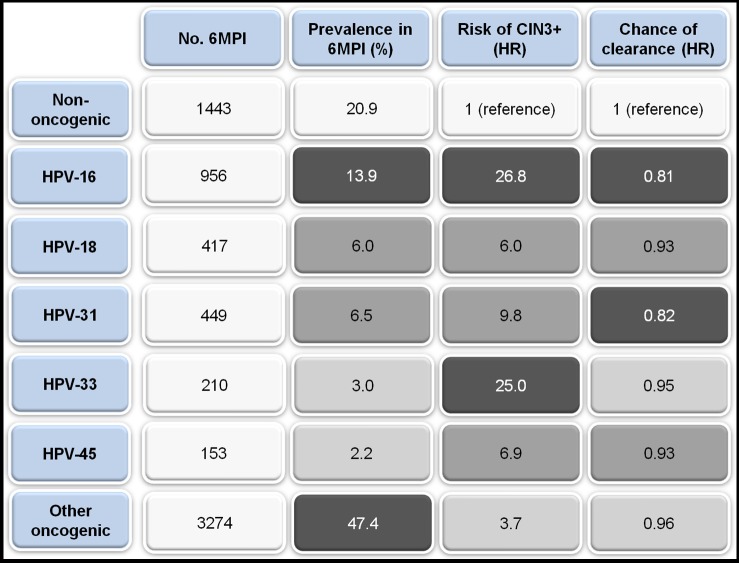# Correction: Natural History of Progression of HPV Infection to Cervical Lesion or Clearance: Analysis of the Control Arm of the Large, Randomised PATRICIA Study

**DOI:** 10.1371/annotation/cea59317-929c-464a-b3f7-e095248f229a

**Published:** 2013-12-31

**Authors:** Unnop Jaisamrarn, Xavier Castellsagué, Suzanne M. Garland, Paulo Naud, Johanna Palmroth, Maria Rowena Del Rosario-Raymundo, Cosette M. Wheeler, Jorge Salmerón, Song-Nan Chow, Dan Apter, Julio C. Teixeira, S. Rachel Skinner, James Hedrick, Anne Szarewski, Barbara Romanowski, Fred Y. Aoki, Tino F. Schwarz, Willy A. J. Poppe, F. Xavier Bosch, Newton S. de Carvalho, Maria Julieta Germar, Klaus Peters, Jorma Paavonen, Marie-Cecile Bozonnat, Dominique Descamps, Frank Struyf, Gary O. Dubin, Dominique Rosillon, Laurence Baril, HPV PATRICIA Study Group

Much of Figure 1 was removed due to a cropping error. Additionally, the color of each figure was incorrectly modified which may affect the readability. Please see the corrected figures here:

Figure 1: 

**Figure:**
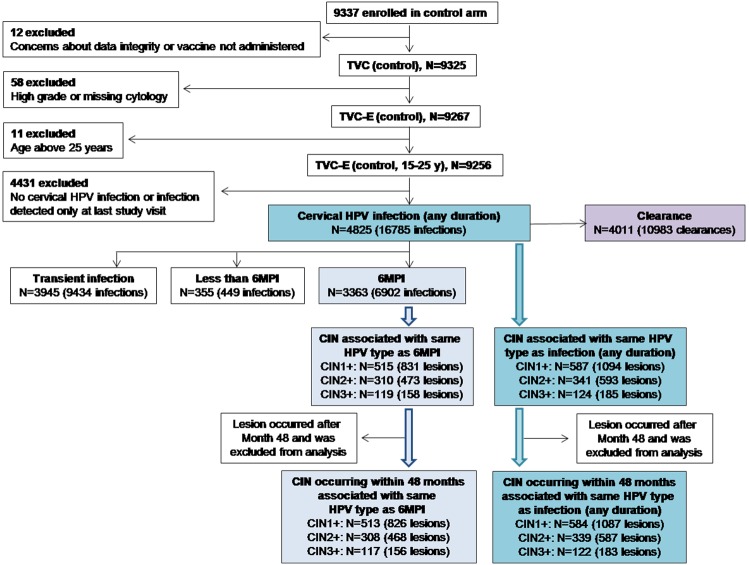


Figure 2: 

**Figure:**
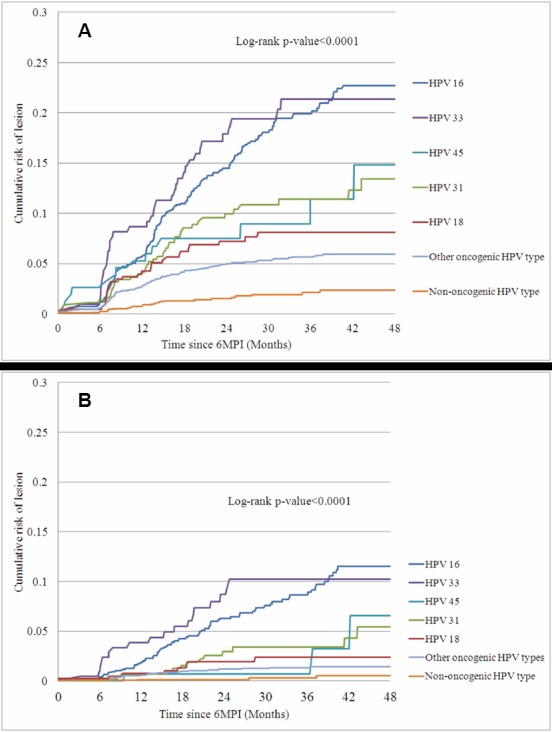


Figure 3: 

**Figure:**
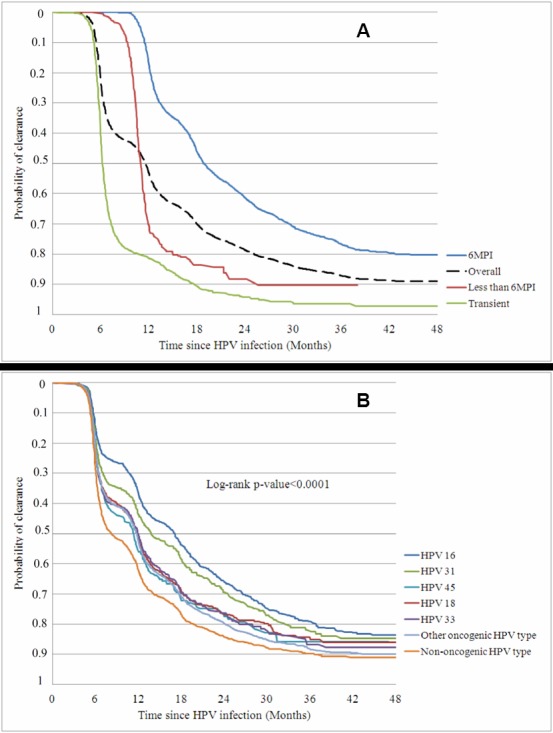


Figure 4: 

**Figure:**