# Supplementary material for: Natural History of Progression of HPV Infection to Cervical Lesion or Clearance: Analysis of the Control Arm of the Large, Randomised PATRICIA Study
Source: PLoS One. 2013 Nov 19;8(11):e79260. doi: 10.1371/journal.pone.0079260 (PMC3834039; doi:10.1371/journal.pone.0079260)
Supplement: File S1 — Supporting Files Table S1. List of Independent Ethics Committees/ Institutional Review Boards. Table S2. Risk of progression of a 6-month persistent cervical HPV infection to a CIN1+ lesion associated with the same HPV type. Table S3. Risk of progression of a 6-month persistent cervical HPV infection to a CIN2+ lesion associated with the same HPV type. Table S4. Risk of progression of a 6-month persistent cervical HPV infection to a CIN3+ lesion associated with the same HPV type. Table S5. Risk of progression of an HPV infection of any duration to a CIN1+ lesion associated with the same HPV type. Table S6. Risk of progression of an HPV infection of any duration to a CIN2+ lesion associated with the same HPV type. Table S7. Risk of progression of an HPV infection of any duration to a CIN3+ lesion associated with the same HPV type. Table S8. Clearance and median duration of a cervical HPV infection. (DOCX) [file pone.0079260.s001.docx]

# Supporting information: U Jaisamrarn et al. Natural history of progression of HPV infection to cervical lesion or clearance: analysis of the control arm of the large, randomised PATRICIA study

**Table S1. List of Independent Ethics Committees/ Institutional Review Boards**

| **Centre Number(s) *** | **Ethics Review Body** | **Location** |
| --- | --- | --- |
| 004538 | Royal Women’s Hospital  132 Grattan Street-Carlton 3053 | AUSTRALIA |
| 004539 | Alfred Human Research Ethics Committee  Alfred Hospital-Commercial Road  Melbourne 3004 |  |
| 004980 | Southern Tasmania Health and Medical Research Ethics Committee  University of Tasmania  Research and Development Office  Private Bag 01-Hobart Tasmania 7001 |  |
| 004981 | King Edward Memorial Hospital for Women  374 Bagot Rd Subiaco WA 6008  St John of God Healthcare  175 Cambridge St Subiaco WA 6008 |  |
| 004993 | Royal Adelaide Hospital Research Ethics Committee  Level 3 Hansen Centre  Rpoyal Adelaide Hospital North terrace  Adelaide SA 5000 |  |
| 005152 | Sydney West Area Health Service Human Research Ethics Committee  Westmead Hospital  Westmead NSW2145 |  |
| 004552 | Commissie voor Medische Ethiek  Universitair Ziekenhuis Antwerpen (UZA)  Wilrijkstraat 10-2650 Edegem | BELGIUM |
| 004553 | Commissie Medische Ethiek  Akademisch Ziekenhuis-VUB  Laarbeeklaan 101-1090 Brussels |  |
| 004556 | Commissie Medische Ethiek  Klinisch Onderzoek-Faculteit Geneeskunde  Herestraat 49-30Leuven |  |
| 005013 | Comitê de Ética em Pesquisa  Faculdade de Ciências Médicas - UNICAMP  Cidade Universitária Zeferino vaz  Rua Tessalia Vieira de Camargo, 126 - caixa postal: 6111  13083-970 - Campinas / SP - Brasil | BRAZIL |
| 005014 | Comitê de Ética em Pesquisa em Seres Humanos do Hospital de  Clínicas da Universidade Federal do Paraná  Rua General Carneiro, 181  80060-160 - Curitiba / PR |  |
| 005017 | Comitê de Ética do Hospital de Clínicas de Porto Alegre  Rua Ramiro Barcelos, 2350 - 2o andar - Bonfim  90035-003 - Porto Alegre / RS |  |
| 005548-005553-  005554-005555-  005556-14594 | Research Review Board Inc.  119 University Avenue East  Waterloo ON, N2J 2W1 | CANADA |
| 005549 | Biomedical Ethics research Board  University of Manitoba  P126-770 Bannatyne Avenue  Winnipeg MB, R3E0W3 |  |
| 005550 | Research Ethics Committee  Sir Mortimer B.Davis Jewish General Hospital  3755 Cote Ste Catherine Road a-925  MONTREAL QC, H3T 1E2 |  |
| 005551 | Comité d.Ethique de la Recherche Clinique CHUL  Centre Hospitalier Universitaire de Quebec . CHUL  Laboratoire et Service d.Infectiologie  2705 Boul Laurier local S-745E  Sainte Foy QC, G1V 4G2 |  |
| 005552 | Health research Ethics Board  University of Alberta  213 Heritage Medical Research Building  Edmonton AB T6G 2S2 |  |
| 004880-04881-  04882-04885-  04886-04887-  04888-04889-  04890-04891-  04892-04893-  09461-12292-  12293-12294-  12296-12297-15982 | ETENE-Lääketieteellinen tutkimuseettinen jaosto  Sosiaali- ja terveysministeriö  Kirkkokatu 4  00170 Helsinki | FINLAND |
| 004322-04551-  10833-11270-  11271-11272-  11273-11274-  11276-11277-  11278-11279-  11280-11281-  11282-11284-  12115 | Ethikkommission der Bayerischen Landesärztekammer  Mühlbaurstr 16-81677 Munchen | GERMANY |
| 004515 | Comitato Etico Provinciale di Modena  Policlinico di Modena Azienda Ospedaliera  Via del Pozzo 71-41100 Modena | ITALY |
| 004516 | Comitato etico della Fondazione IRCCS  Ospedale Maggiore Policlinico Mangiagalli e Regina Elena  Via F Sforza 35 -20112, Milan |  |
| 5033 | Comisión Nacional de Investigación Cientifica y Subcomision de Etica  del Instituto Mexicano del Seguro Social  4°piso Edificio B Unidad de Congresos  Avenida Cuahtemoc 330 Colonia Doctores Mexico DF 06725 | MEXICO |
| 004425 | The Ethics Committee  San Pablo Colleges Medical centre  Barangay San Rafael Maharlika Highway  4000 San Pablo City Laguna | PHILIPPINES |
| 005114 | Institutional Review Board  Makati Medical Center  2 Amorsolo street Makati City |  |
| 005116 | De La Salle health Sciences Campus Ethics Rreview Board  (DLS-HSC-ERB)  De La Salle Angelo King Medical Research Center  Congressional Avenue, Dasmarinas Cavite 4114 |  |
| 005692 | Institutional Review Board  Calamba Medical Center . Crossing Calamba Laguna |  |
| 009976 | Ethics Review Board-Medical Arts Building  University of Perpetual Help Medical Center  Alabang-Zapote Road- Las Pinas City |  |
| 009977 | Committee on research Implementation and development and review  Board  Healthserv-Los Banos Inc,8817 National Highway  Los Banos Laguna |  |
| 009978 | Research Implementation and Development Office  College of Medicine  University of the Philippines taft Avenue Manila 1000 |  |
| 004569 | Comite Etico de Investigacion Clinica Del Hopsital Clinic I Provincial de  Barcelona  C/Villarroel 170 . 08036 Barcelona | SPAIN |
| 004570 | Comité Etico de Investigacion Clinica Hospital San Carlos  Martin Lagos s/n-28040 Madrid |  |
| 004572 | Comité Etico de Investigacion Clinica Hospital Vall D.Hebron  Passeig de la Vall d.Hebron 119-129 08035 Barcelona |  |
| 005279 | Comite Etico De Investigacon Clinica Del Hospital De Mostoles  C/Rio Jucar s/n 28935 Mostoles madrid |  |
| 005280 | Comite Etico De Investigacion Clinica Ciudad Sanitaria de Bellvitge  Feixa Llarga s/n  08907 L.Hospitalet de Llobregat |  |
| 005281 | Comite Etico de Investigacion Clinica  Hospital Universitario de La Princesa  C/Diego d Leon 62 Planta 9a Sector 2-28006 Madrid |  |
| 4927, 014244, 4955,  4951, 4952, 013831,  4923, 14689, 14690,  4925, 014245, 4928,  8233, 4931, 9039,  5475, 4934, 013833,  4940, 4945, 8231,  13835, 4947,  014243, 013834,  4944, 4943, 4937,  9066, 9068, 4939 | Quorum Review IRB 1601 Fifth Ave., Suite 1000  Seattle, WA 98101 | US |
| 4930 | Local/University of Minnestota  IRB  Mayo Mauil Code 820  D-528 Mayo Memorial Bldg  420 Delaware Stret S. E.  Minneapolis, MN 55455 |  |
| 4932 | Local/Medical College of Georgia 1120 15th Street CJ-2103  Augusta, GA 30912 |  |
| 4933 | Local/Univ. of Louisville  MedCenter One, Suite 200  501 E. Broadway  Louisville, KY 40202-1798 |  |
| 4920 | Western Institutional Review Board  3535 Seventh Ave, SW  Olympia, WA 98502 |  |
| 4946 | Hiawaii Pacific Health Res. Institute  1100 Ward Ave.  Suite 1045  Honolulu, Hawaii 96814 |  |
| 14460 | University of Oklahoma  Office of Research Administration Stanton Young  Blvd., LIB 121  Oklahoma City, OK 73117 |  |
| 4938 | Local/IRB for Health Scoences Research  PO Box 800483  University of Virginia  Charlottesville, VA 22908 |  |
| 4971 | Local/Morristown Atlantic Health Sys.  IRB  95 Madison Ave.  Morristown, NJ 07960 |  |
| 4958 | Biomedical Research Alliance of New York, LLC  Institituional Review Board  225 Community Drive, Suite 100 Great Neck, NY 11021 |  |
| 4949 | Local/Human Subjects Office  340 Medicine Administration Bldg  Iowa City, Iowa 52242-1101 |  |
| 4918 | Local/The MetroHealth System IRB  MetroHealth Medical Center  2500 MetroHealth Dr.  Cleveland, OH 44109-1998 |  |
| 4941 | Local/University of Miami  1500 NW 12 Ave Suite 1000  Miami, FL 33136 |  |
| 4929 | Local/  Human Research Review Committee  MSC 08 4560 BMSB Room B71  1 University Of New Mexico  Albuquerque, NM 87131-0001 |  |
| 004543 | Ethics Committee  National Taiwan University Hospital  7 Chung Shan South Road Taipei 100 | TAIWAN |
| 004544 | Tri-Service General Hospital Institutional Review Board  N°325,Sec 2, Cheng-gong Road, Neihu District 114 Taipei |  |
| 011406 | Taipei Veterans General Hospital Institutional review Board  N° 201, Sec 2, Shih-Pai Road Taipei 112 |  |
| 003891 | Ethical Review Committee  Royal Thai Army Medical Department  315 Rajavithi Road, Rajathevee Bangkok 10400 | THAILAND |
| 003892 | Research Affairs  Faculty of Medicine Chulalongkorn University  1873 Rama Itumwan Bangkok 10330 |  |
| 003893 | Ethics Coimmittee Faculty of Medicine Siriraj Hospital Mahidol  University  2 Pranok Road Bangkoknoi Bangkok 10700 |  |
| 04859-04861-  04894-14372-14373 | NorthWest Multi Centre Research Ethics Committee  Gateway House  Picadilly South, Manchester M60 7LP | UK |
| 5051 | Grampian LREC  Grampian NHS Board  Summerfield House, 2 Eday Road, Aberdeen, AB15 6RE |  |
| 4859 & 14373 | Central Manchester LREC  Room 181, 1st Floor, Gateway House  Piccadilly South, Manchester, M60 7LP |  |
| 4894 | Camden & Islington Community LREC  Room 3/14, 3rd Floor, West Wing, St Pancras Hospital  4 St Pancras Way, London, NW1 0PE |  |

* GSK Biologicals assigned centre number

**Table S2. Risk of progression of a 6-month persistent cervical HPV infection to a CIN1+ lesion associated with the same HPV type**

| **Determinant** | **Category** | **No. 6MPI N=6902** | **No. 6MPI with CIN1+**  **N=826** | **Probability of developing CIN1+ at month 24 (95% CI)** | **Univariate analysis** | | **Multivariable analysis^1,2^** | |
| --- | --- | --- | --- | --- | --- | --- | --- | --- |
|  |  |  |  |  | **Hazard ratio  (95% CI)** | **p-value** | **Hazard ratio  (95% CI)** | **p-value** |
| HPV type | Non-oncogenic type | 1443 | 71 | 0.05 (0.04–0.06) | 1 | - | 1 | - |
|  | HPV-16 | 956 | 208 | 0.20 (0.17–0.22) | 4.47 (3.39–5.88) | <0.0001 | 4.60 (3.47–6.09) | <0.0001 |
|  | HPV-18 | 417 | 52 | 0.12 (0.10–0.16) | 2.58 (1.81–3.67) | <0.0001 | 2.43 (1.70–3.48) | <0.0001 |
|  | HPV-31 | 449 | 75 | 0.17 (0.14–0.21) | 3.57 (2.58–4.94) | <0.0001 | 3.33 (2.39–4.64) | <0.0001 |
|  | HPV-33 | 210 | 50 | 0.24 (0.18–0.31) | 5.02 (3.53–7.13) | <0.0001 | 4.44 (3.12–6.32) | <0.0001 |
|  | HPV-45 | 153 | 20 | 0.11 (0.07–0.17) | 2.75 (1.67–4.52) | <0.0001 | 2.53 (1.53–4.17) | 0.0003 |
|  | Other oncogenic type | 3274 | 350 | 0.11 (0.10–0.12) | 2.19 (1.69–2.84) | <0.0001 | 2.16 (1.66–2.81) | <0.0001 |
|  |  |  |  | *<0.0001* | *<0.0001* | | *<0.0001* | |
| Region | Europe | 3384 | 443 | 0.13 (0.12–0.14) | 1 |  | 1 | - |
|  | Asia Pacific | 1281 | 131 | 0.10 (0.09–0.12) | 0.75 (0.57–0.97) | 0.0280 | 1.16 (0.87–1.54) | 0.3202 |
|  | Latin America | 1184 | 117 | 0.09 (0.07–0.10) | 0.70 (0.53–0.91) | 0.0091 | 0.90 (0.68–1.19) | 0.4592 |
|  | North America | 1053 | 135 | 0.13 (0.11–0.16) | 0.99 (0.77–1.28) | 0.9467 | 1.21 (0.93–1.56) | 0.1478 |
|  |  |  |  | *0.0004* | *0.0157* | | *0.1970* | |
| Tobacco exposure (number of pack- years) | <0.5 | 3945 | 402 | 0.10 (0.09–0.11) | 1 | - | 1 | - |
|  | ≥0.5 | 2925 | 422 | 0.14 (0.13–0.16) | 1.47 (1.22–1.76) | 0.0001 | 1.25 (1.02–1.53) | 0.0335 |
|  |  |  |  | *<0.0001* | *<0.0001* | | *0.0335* | |
| Age at first sexual intercourse | ≥18 years or never had sexual intercourse | 1814 | 162 | 0.09 (0.08–0.11) | 1 | - | 1 | - |
|  | 15-17 years | 3731 | 452 | 0.12 (0.11–0.13) | 1.37 (1.08–1.73) | 0.0088 | 1.13 (0.90–1.43) | 0.2881 |
|  | <15 years | 1355 | 212 | 0.15 (0.13–0.17) | 1.78 (1.36–2.34) | <0.0001 | 1.21 (0.91–1.62) | 0.1846 |
|  |  |  |  | *<0.0001* | *0.0002* | | *0.3987* | |
| Number of sexual partners during the past 12 months | 0–1 | 3706 | 393 | 0.10 (0.09–0.11) | 1 | - | 1 | - |
|  | 2-3 | 2394 | 297 | 0.12 (0.11–0.14) | 1.20 (0.99–1.44) | 0.0602 | 1.00 (0.83–1.20) | 0.9707 |
|  | ≥4 | 781 | 133 | 0.18 (0.15–0.21) | 1.77 (1.34–2.34) | <0.0001 | 1.33 (1.01–1.77) | 0.0447 |
|  |  |  |  | *<0.0001* | *0.0002* | | *0.0999* | |
| History of *Chlamydia trachomatis* during the past 12 months | No | 6092 | 738 | 0.12 (0.11–0.13) | 1 | - | Not included | |
|  | Yes | 782 | 82 | 0.10 (0.08–0.12) | 0.84 (0.63–1.11) | 0.2106 |  |  |
|  |  |  |  | *0.1247* | *0.2106* | |  | |
| Marital/partner status | Single | 5340 | 648 | 0.12 (0.10–0.14) | 1 | - | Not included | |
|  | Living or lived with partner | 1487 | 169 | 0.12 (0.11–0.13) | 0.96 (0.77–1.19) | 0.7061 |  | |
|  |  |  |  | *0.6269* | *0.7061* | |  | |
| Previous pregancy | No | 6154 | 698 | 0.11 (0.11–0.12) | 1 | - | 1 | - |
|  | Yes | 744 | 128 | 0.15 (0.12–0.18) | 1.36 (1.08–1.70) | 0.0080 | 1.63 (1.29–2.06) | <0.0001 |
|  |  |  |  | *0.0015* | *0.0080* | | *<0.0001* | |
| Use of hormones for contraception or other indication | No | 1589 | 152 | 0.10 (0.08–0.12) | 1 | - | 1 | - |
|  | Yes | 5285 | 668 | 0.12 (0.11–0.13) | 1.37 (1.11–1.70) | 0.0039 | 1.18 (0.95–1.47) | 0.1353 |
|  |  |  |  | *0.0004* | *0.00391* | | *0.1353* | |
| Use of intrauterine device | No | 6658 | 795 | 0.12 (0.11–0.13) | 1 | - | Not included | |
|  | Yes | 216 | 25 | 0.13 (0.09–0.19) | 0.93 (0.61–1.41) | 0.7350 |  | |
|  |  |  |  | *0.7237* | *0.7350* | |  | |
|  | No | 3348 | 382 | 0.10 (0.09–0.11) | 1 | - | 1 | - |
|  | Yes (at least 1 oncogenic HPV type) | 3203 | 409 | 0.14 (0.13–0.15) | 1.42 (1.21–1.67) | <0.0001 | 1.32 (1.11–1.56) | 0.0016 |
|  | Yes (only non-oncogenic HPV types) | 351 | 35 | 0.11 (0.08–0.15) | 1.01 (0.68–1.52) | 0.9426 | 1.15 (0.78–1.70) | 0.4846 |
|  |  |  |  | *<0.0001* | *<0.0001* | | *0.0071* | |
| Previous cervical HPV infection | No | 2686 | 209 |  | 1 |  | 1 | - |
|  | Yes (at least 1 oncogenic HPV type) | 3641 | 571 |  | 2.00 (1.65–2.42) | <0.0001 | 1.75 (1.44–2.12) | <0.0001 |
|  | Yes (only non-oncogenic HPV types) | 575 | 46 |  | 1.11 (0.80–1.54) | 0.5289 | 1.05 (0.76–1.46) | 0.7689 |
|  |  |  |  | *Not done^3^* | *<0.0001* | | *<0.0001* | |
| Previous CIN1+^4^ | No | 6672 | 784 | 0.12 (0.11–0.12) | 1 | - | 1 | - |
|  | Yes (at least 1 oncogenic HPV type) | 230 | 42 | 0.20 (0.15–0.26) | 2.34 (1.53–3.56) | <0.0001 | 1.94 (1.21–3.12) | 0.0059 |
|  | Yes (only non-oncogenic HPV types) | 0 | - |  | - |  | - |  |
|  |  |  |  | *<0.0001* | *<0.0001* | | *0.0059* | |
| Concomitant CIN1+ ^5,3^ | No | 6258 | 747 |  | 1 |  | 1 | - |
|  | Yes (with either oncogenic or non-oncogenic HPV type) | 644 | 79 |  | 2.84 (2.15–3.75) | <0.0001 | 2.27 (1.67–3.08) | <0.0001 |
|  |  |  |  | *Not done^3^* | *<0.0001* | | *<0.0001* | |

^1^Covariates were included in the multivariable analysis if they had a global p-value of <0.2 in the univariate analysis (except region which was always included).

^2^Infections or lesions with a missing value for a covariate included in the analysis were excluded from the multivariable analysis. The analysis included 3337 women with 6835 6MPI and 818 lesions.

^3^Time-varying covariates: Kaplan-Meier analysis not done.

^4^CIN1+ associated with an HPV type different to the reference infection, preceding the onset of the 6MPI

^5^CIN1+ associated with an HPV type different to the reference infection, concomitant to the 6MPI (following its onset and preceding its end)

Values in italics show the log-rank p-value (for the probability of developing a lesion) or the global p-value (for the univariate and multivariable analyses).

6MPI: 6-month persistent infection; CIN: cervical intraepithelial neoplasia; HPV: human papillomavirus

**Table S3. Risk of progression of a 6-month persistent cervical HPV infection to a CIN2+ lesion associated with the same HPV type**

| **Determinant** | **Category** | **No. 6MPI N=6902** | | **No. 6MPI with CIN2+**  **N=468** | **Probability of developing CIN2+ at month 24 (95% CI)** | **Univariate analysis** | | | | | **Multivariable analysis^1,2^** | | | | |
| --- | --- | --- | --- | --- | --- | --- | --- | --- | --- | --- | --- | --- | --- | --- | --- |
|  |  |  | |  |  | **Hazard ratio  (95% CI)** | | **p-value** | | | **Hazard ratio  (95% CI)** | | | **p-value** | |
| HPV type | Non-oncogenic type | 1443 | | 24 | 0.02 (0.01–0.02) | 1 | | - | | | 1 | | | - | |
|  | HPV-16 | 956 | | 162 | 0.14 (0.12–0.17) | 10.16 (6.76–15.27) | | <0.0001 | | | 10.44 (6.96–15.65) | | | <0.0001 | |
|  | HPV-18 | 417 | | 29 | 0.07 (0.05–0.10) | 4.16 (2.57–6.73) | | <0.0001 | | | 3.87 (2.38–6.30) | | | <0.0001 | |
|  | HPV-31 | 449 | | 43 | 0.10 (0.07–0.14) | 5.98 (3.71–9.65) | | <0.0001 | | | 5.68 (3.50–9.21) | | | <0.0001 | |
|  | HPV-33 | 210 | | 38 | 0.18 (0.13–0.24) | 11.08 (6.85–17.93) | | <0.0001 | | | 9.65 (5.97–15.60) | | | <0.0001 | |
|  | HPV-45 | 153 | | 14 | 0.07 (0.04–0.13) | 5.71 (3.05–10.70) | | <0.0001 | | | 5.38 (2.87–10.06) | | | <0.0001 | |
|  | Other oncogenic type | 3274 | | 158 | 0.05 (0.04–0.06) | 2.86 (1.92–4.28) | | <0.0001 | | | 2.80 (1.87–4.19) | | | <0.0001 | |
|  |  |  | |  | *<0.0001* | *<0.0001* | | | | | *<0.0001* | | | | |
| Region | Europe | 3384 | | 235 | 0.07 (0.06–0.08) | 1 | | | - | | 1 | | | - | |
|  | Asia Pacific | 1281 | | 78 | 0.06 (0.05–0.08) | 0.84 (0.60–1.18) | | | 0.3086 | | 1.48 (0.99–2.21) | | | 0.0531 | |
|  | Latin America | 1184 | | 74 | 0.05 (0.04–0.07) | 0.83 (0.59–1.18) | | | 0.3018 | | 1.17 (0.79–1.72) | | | 0.4305 | |
|  | North America | 1053 | | 81 | 0.08 (0.06–0.10) | 1.12 (0.80–1.56) | | | 0.5204 | | 1.39 (0.97–1.98) | | | 0.0744 | |
|  |  |  | |  | *0.1594* | *0.3836* | | | | | *0.1628* | | | | |
| Tobacco exposure (number of pack-years) | None or less than 6 months (<0.5) | 3945 | | 230 | 0.06 (0.05–0.06) | 1 | | | - | | 1 | | | - | |
|  | At least 6 months (≥0.5) | 2925 | | 236 | 0.08 (0.07–0.09) | 1.42 (1.11–1.81) | | | 0.0049 | | 1.18 (0.89–1.57) | | | 0.2505 | |
|  |  |  | |  | *0.0001* | *0.0049* | | | | | *0.2505* | | | | |
| Age at first sexual intercourse | ≥18 years or never had sexual intercourse | | 1814 | 85 | 0.05 (0.04–0.06) | 1 | | - | | | 1 | | | | - |
|  | 15-17 years | | 3731 | 257 | 0.07 (0.06–0.08) | 1.48 (1.08–2.03) | | 0.0161 | | | 1.25 (0.90–1.73) | | | | 0.1775 |
|  | <15 years | | 1355 | 126 | 0.08 (0.07–0.10) | 1.99 (1.38–2.86) | | 0.0002 | | | 1.40 (0.93–2.10) | | | | 0.1032 |
|  |  | |  |  | *<0.0001* | *0.0010* | | *0.2489* | | | | | | | |
| Number of sexual partners during the past 12 months | 0–1 | | 3706 | 230 | 0.06 (0.05–0.07) | 1 | | - | | | 1 | | | |  |
|  | 2-3 | | 2394 | 157 | 0.06 (0.05–0.07) | 1.08 (0.84–1.37) | | 0.5551 | | | 0.92 (0.72–1.19) | | | | 0.5422 |
|  | ≥4 | | 781 | 80 | 0.11 (0.09–0.14) | 1.83 (1.28–2.60) | | 0.0009 | | | 1.45 (0.99–2.12) | | | | 0.0581 |
|  |  | |  |  | *<0.0001* | *0.0035* | | *0.0679* | | | | | | | |
| History of *Chlamydia trachomatis* during the past 12 months | No | | 6092 | 413 | 0.07 (0.06–0.07) | 1 | | - | | | Not included | | | | |
|  | Yes | | 782 | 53 | 0.06 (0.05–0.09) | 0.96 (0.67–1.38) | | 0.8438 | | |  | | | | |
|  |  | |  |  | *0.8017* | *0.8438* | |  | | | | | | | |
| Marital/partner status | Single | | 5340 | 369 | 0.07 (0.06–0.07) | 1 | | - | | | Not included | | | | |
|  | Living or lived with partner | | 1487 | 95 | 0.07 (0.05–0.08) | 0.95 (0.72–1.27) | | 0.7478 | | |  | | | | |
|  |  | |  |  | *0.6791* | *0.7478* | |  | | | | | | | |
| Previous pregnancy | No | | 6154 | 381 | 0.06 (0.06–0.07) | 1 | | - | | | 1 | | - | | |
|  | Yes | | 744 | 87 | 0.10 (0.08–0.12) | 1.64 (1.24–2.16) | | 0.0005 | | | 1.75 (1.30–2.35) | | 0.0002 | | |
|  |  | |  |  | *<0.0001* | *0.0005* | | *0.0002* | | | | | | | |
| Use of hormones for contraception or other indication | No | | 1589 | 73 | 0.05 (0.04–0.06) | 1 | | - | | | 1 | | - | | |
|  | Yes | | 5285 | 393 | 0.07 (0.06–0.08) | 1.70 (1.26–2.28) | | 0.0004 | | | 1.49 (1.09–2.03) | | 0.0117 | | |
|  |  | |  |  | *<0.0001* | *0.0004* | | *0.0117* | | | | | | | |
| Use of intrauterine device | No | | 6658 | 453 | 0.07 (0.06–0.07) | 1 | | - | | | | Not included | | | |
|  | Yes | | 216 | 13 | 0.07 (0.04–0.12) | 0.83 (0.46–1.50) | | 0.5422 | | | |  | | | |
|  |  | |  |  | *0.5121* | *0.5422* | |  | | | | | | | |
| Cervical HPV co-infections detected up to occurrence of lesion^3^ | No | | 2704 | 111 |  | | 1 | | | - | | 1 | - | | |
|  | Yes (at least 1 oncogenic HPV type) | | 3627 | 337 |  | | 2.10 (1.64–2.69) | | | <0.0001 | | 1.88 (1.46–2.41) | <0.0001 | | |
|  | Yes (only non-oncogenic HPV types) | | 571 | 20 |  | | 0.86 (0.53–1.39) | | | 0.5373 | | 0.80 (0.49–1.31) | 0.3731 | | |
|  |  | |  |  | *Not done^3^* | | *<0.0001* | | | | | *<0.0001* | | | |
| Previous cervical HPV infection | No | | 3348 | 239 | 0.06 (0.05–0.07) | | 1 | | | - | | Not included | | | |
|  | Yes (at least 1 oncogenic HPV type) | | 3203 | 209 | 0.07 (0.06–0.08) | | 1.19 (0.96–1.48) | | | 0.1069 | |  |  | | |
|  | Yes (only non-oncogenic HPV types) | | 351 | 20 | 0.06 (0.04–0.10) | | 0.96 (0.55–1.68) | | | 0.8782 | |  |  | | |
|  |  | |  |  | *0.1546* | | *0.2384* | | | | |  | | | |
| Previous CIN1+ ^4^ | No | | 6665 | 443 | 0.06 (0.06–0.07) | | 1 | | | - | | 1 | - | | |
|  | Yes (at least 1 oncogenic HPV type) | | 237 | 25 | 0.13 (0.08–0.20) | | 2.46 (1.45–4.16) | | | 0.0009 | | 2.75 (1.58–4.80) | 0.0004 | | |
|  | Yes (only non-oncogenic HPV types) | | 0 | - | - | | - | | | - | | - | - | | |
|  |  | |  |  | *<0.0001* | | *0.0009* | | | | | *0.0004* | | | |
| Concomitant CIN1+ ^5,3^ | No | | 6031 | 402 |  | | 1 | | | - | | 1 | - | | |
|  | Yes (with either oncogenic or non-oncogenic HPV type) | | 871 | 66 |  | | 2.48 (1.77–3.48) | | | <0.0001 | | 2.03 (1.40–2.93) | 0.0002 | | |
|  |  | |  |  | *Not done^3^* | | *<0.0001* | | | | | *0.0002* | | | |

^1^Covariates were included in the multivariable analysis if they had a global p-value of <0.2 in the univariate analysis (except region which was always included).

^2^Infections or lesions with a missing value for a covariate included in the analysis were excluded from the multivariable analysis. The analysis included 3337 women with 6835 6MPI and 818 lesions.

^3^Time-varying covariates: Kaplan-Meier analysis not done.

^4^CIN1+ associated with an HPV type different to the reference infection, preceding the onset of the 6MPI

^5^CIN1+ associated with an HPV type different to the reference infection, concomitant to the 6MPI (following its onset and preceding its end)

Values in italics show the log-rank p-value (for the probability of developing a lesion) or the global p-value (for the univariate and multivariable analyses).

6MPI: 6-month persistent infection; CIN: cervical intraepithelial neoplasia; HPV: human papillomavirus

**Table S4. Risk of progression of a 6-month persistent cervical HPV infection to a CIN3+ lesion associated with the same HPV type**

| **Determinant** | **Category** | **No. 6MPI N=6902** | **No. 6MPI with CIN3+**  **N=156** | **Probability of developing CIN3+ at month 24 (95% CI)** | **Univariate analysis** | | | **Multivariable analysis^1,2^** | |
| --- | --- | --- | --- | --- | --- | --- | --- | --- | --- |
|  |  |  |  |  | **Hazard ratio  (95% CI)** | | **p-value** | **Hazard ratio  (95% CI)** | **p-value** |
| HPV type | Non-oncogenic type | 1443 | 4 | 0.00 (0.00–0.01) | 1 | | - | 1 | - |
|  | HPV-16 | 956 | 73 | 0.06 (0.05–0.08) | 25.86 (9.69–68.96) | | <0.0001 | 26.82 (10.00–71.94) | <0.0001 |
|  | HPV-18 | 417 | 8 | 0.02 (0.01–0.04) | 6.63 (2.00–21.98) | | 0.0020 | 6.04 (1.82–20.04) | 0.0033 |
|  | HPV-31 | 449 | 13 | 0.03 (0.02–0.06) | 10.48 (3.42–32.13) | | <0.0001 | 9.80 (3.16–30.37) | <0.0001 |
|  | HPV-33 | 210 | 18 | 0.09 (0.05–0.14) | 29.61 (10.47–83.74) | | <0.0001 | 25.04 (9.00–69.69) | <0.0001 |
|  | HPV-45 | 153 | 3 | 0.01 (0.00–0.05) | 7.22 (1.62–32.16) | | 0.0095 | 6.88 (1.54–30.74) | 0.0116 |
|  | Other oncogenic type | 3274 | 37 | 0.01 (0.01–0.02) | 3.92 (1.42–10.85) | | 0.0084 | 3.73 (1.34–10.37) | 0.0117 |
|  |  |  |  | *<0.0001* | *<0.0001* | | | *<0.0001* | |
| Region | Europe | 3384 | 70 | 0.02 (0.01–0.03) | 1 | | - | 1 |  |
|  | Asia Pacific | 1281 | 26 | 0.02 (0.02–0.03) | 0.93 (0.54–1.59) | | 0.7800 | 1.40 (0.69–2.81) | 0.3488 |
|  | Latin America | 1184 | 27 | 0.02 (0.01–0.03) | 1.00 (0.58–1.72) | | 0.9932 | 1.39 (0.73–2.66) | 0.3128 |
|  | North America | 1053 | 33 | 0.03 (0.02–0.04) | 1.50 (0.90–2.51) | | 0.1209 | 1.74 (0.96–3.18) | 0.0697 |
|  |  |  |  | *0.1831* | *0.3372* | | | *0.3467* | |
| Tobacco exposure (number of pack- years) | None or less than 6 months (<0.5) | 3945 | 73 | 0.02 (0.01–0.02) | 1 | |  | 1 |  |
|  | At least 6 months (≥0.5) | 2925 | 81 | 0.03 (0.02–0.03) | 1.53 (1.04–2.27) | | 0.0310 | 1.20 (0.76–1.90) | 0.4294 |
|  |  |  |  | *0.0075* | *0.0310* | | | *0.4294* | |
| Age at first sexual intercourse | ≥18 years or never had sexual intercourse | 1814 | 30 | 0.02 (0.01–0.03) | 1 | |  | 1 |  |
|  | 15-17 years | 3731 | 67 | 0.02 (0.01–0.02) | 1.07 (0.64–1.81) | | 0.7905 | 0.89 (0.50–1.57) | 0.6761 |
|  | <15 years | 1355 | 59 | 0.04 (0.03–0.05) | 2.58 (1.48–4.51) | | 0.0008 | 1.69 (0.86–3.34) | 0.1296 |
|  |  |  |  | *<0.0001* | *0.0001* | | | *0.0351* | |
| Number of sexual partners during the past 12 months | 0–1 | 3706 | 82 | 0.02 (0.02–0.03) | 1 | |  | Not included | |
|  | 2-3 | 2394 | 50 | 0.02 (0.01–0.02) | 0.96 (0.63–1.45) | | 0.8376 |  |  |
|  | ≥4 | 781 | 24 | 0.04 (0.02–0.06) | 1.52 (0.87–2.66) | | 0.1400 |  |  |
|  |  |  |  | *0.1364* | *0.2624* | | |  | |
| History of *Chlamydia trachomatis* during the past 12 months | No | 6092 | 137 | 0.02 (0.02–0.03) | 1 | |  | Not included | |
|  | Yes | 782 | 19 | 0.02 (0.01–0.04) | 1.01 (0.58–1.78) | | 0.9640 |  | |
|  |  |  |  | *0.9581* | *0.9640* | | |  | |
| Marital/partner status | Single | 5340 | 113 | 0.03 (0.02–0.04) | 1 | |  | 1 |  |
|  | Living or lived with partner | 1487 | 41 | 0.03 (0.02–0.04) | 1.36 (0.90–2.06) | | 0.1446 | 1.31 (0.85–2.04) | 0.2255 |
|  |  |  |  | *0.0896* | *0.1446* | | | *0.2255* | |
| Previous pregnancy | No | 6154 | 110 | 0.02 (0.01–0.02) | 1 | |  | 1 |  |
|  | Yes | 744 | 46 | 0.05 (0.03–0.07) | 2.77 (1.88–4.07) | | <0.0001 | 2.63 (1.68–4.11) | <0.0001 |
|  |  |  |  | *<0.0001* | *<0.0001* | | | *<0.0001* | |
| Use of hormones for contraception or other indication | No | 1589 | 28 | 0.02 (0.01–0.03) | 1 | |  | 1 |  |
|  | Yes | 5285 | 128 | 0.02 (0.02–0.03) | 1.44 (0.87–2.37) | | 0.1518 | 1.19 (0.67–2.12) | 0.5473 |
|  |  |  |  | *0.0781* | *0.1518* | | | *0.5473* | |
| Use of intrauterine device | No | 6658 | 8 | 0.02 (0.02–0.02) | 1 | |  | Not included | |
|  | Yes | 216 | 8 | 0.04 (0.02–0.08) | 1.56 (0.70–3.47) | | 0.2767 |  | |
|  |  |  |  | *0.2172* | *0.2767* | | |  | |
| Cervical HPV co-infections detected up to occurrence of lesion^3^ | No | 2798 | 47 |  | 1 | |  | 1 |  |
|  | Yes (at least 1 oncogenic HPV type) | 3524 | 101 | *Not done^3^* | 1.53 (1.04–2.25) | | 0.0327 | 1.36 (0.94–1.98) | 0.1018 |
|  | Yes (only non-oncogenic HPV types) | 580 | 8 |  | 0.81 (0.38–1.72) | | 0.5771 | 0.74 (0.33–1.66) | 0.4657 |
|  |  |  |  |  | *0.0392* | | | *0.0955* | |
| Previous cervical HPV infection | No | 3348 | 90 | 0.02 (0.02–0.03) | 1 | |  | Not included | |
|  | Yes (at least 1 oncogenic HPV type) | 3203 | 55 | 0.02 (0.02–0.03) | 0.93 (0.65–1.35) | | 0.7052 |  |  |
|  | Yes (only non-oncogenic HPV types) | 351 | 11 | 0.03 (0.01–0.06) | 1.56 (0.67–3.62) | | 0.3044 |  |  |
|  |  |  |  | *0.2908* | *0.5052* | | |  | |
| Previous CIN1+^4^ | No | 6664 | 147 | 0.02 (0.02–0.02) | 1 |  | | 1 |  |
|  | Yes (at least 1 oncogenic HPV type) | 238 | 9 | 0.04 (0.02–0.09) | 2.98 (1.42–6.23) | 0.0037 | | 3.78 (1.69–8.41) | 0.0012 |
|  | Yes (only non-oncogenic HPV types) | - | - | - | - | - | | - | - |
|  |  |  |  | *0.0009* | *0.0037* | | | *0.0012* | |
| Concomitant CIN1+ ^5,3^ | No | 5834 | 108 |  | 1 |  | | 1 |  |
|  | Yes (with either oncogenic or non-oncogenic HPV type) | 1068 | 48 | *Not done^3^* | 4.42 (2.72–7.16) | <0.0001 | | 3.86 (2.31–6.47) | <0.0001 |
|  |  |  |  |  | *<0.0001* | | | *<0.0001* | |

^1^Covariates were included in the multivariable analysis if they had a global p-value of <0.2 in the univariate analysis (except region which was always included).

^2^Infections or lesions with a missing value for a covariate included in the analysis were excluded from the multivariable analysis. The analysis included 3337 women with 6835 6MPI and 818 lesions.

^3^Time-varying covariates: Kaplan-Meier analysis not done.

^4^CIN1+ associated with an HPV type different to the reference infection, preceding the onset of the 6MPI

^5^CIN1+ associated with an HPV type different to the reference infection, concomitant to the 6MPI (following its onset and preceding its end)

Values in italics show the log-rank p-value (for the probability of developing a lesion) or the global p-value (for the univariate and multivariable analyses).

6MPI: 6-month persistent infection; CIN: cervical intraepithelial neoplasia; HPV: human papillomavirus

**Table S5. Risk of progression of an HPV infection of any duration to a CIN1+ lesion associated with the same HPV type**

| **Determinant** | **Category** | **No. infections N=16785** | **No. infections with CIN1+**  **N=1087** | **Probability of developing CIN1+ at month 24 (95% CI)** | **Univariate analysis** | | **Multivariable analysis^1,2^** | |
| --- | --- | --- | --- | --- | --- | --- | --- | --- |
|  |  |  |  |  | **Hazard ratio  (95% CI)** | **p-value** | **Hazard ratio  (95% CI)** | **p-value** |
| Duration of infection | Transient | 9434 | 231 | 0.03 (0.02–0.03) | 1 | - | 1 | - |
|  | Less than 6MPI | 449 | 30 | 0.08 (0.05–0.11) | 2.90 (1.83–4.59) | <0.0001 | 2.48 (1.54–3.98) | 0.0002 |
|  | 6MPI | 6902 | 826 | 0.12 (0.11–0.13) | 4.76 (4.09–5.53) | <0.0001 | 4.25 (3.63–4.97) | <0.0001 |
|  |  |  |  | *<0.0001* | *<0.0001* | | *<0.0001* | |
| HPV type | Non-oncogenic type | 4824 | 117 | 0.02 (0.02–0.03) | 1 | - | 1 | - |
|  | HPV-16 | 1557 | 227 | 0.14 (0.12–0.16) | 6.01 (4.86–7.43) | <0.0001 | 4.39 (3.52–5.47) | <0.0001 |
|  | HPV-18 | 945 | 70 | 0.08 (0.06–0.10) | 3.07 (2.32–4.07) | <0.0001 | 2.47 (1.85–3.30) | <0.0001 |
|  | HPV-31 | 914 | 97 | 0.11 (0.09–0.14) | 4.55 (3.53–5.86) | <0.0001 | 3.34 (2.57–4.34) | <0.0001 |
|  | HPV-33 | 488 | 65 | 0.14 (0.11–0.18) | 5.68 (4.28–7.53) | <0.0001 | 4.36 (3.29–5.79) | <0.0001 |
|  | HPV-45 | 391 | 25 | 0.06 (0.04–0.09) | 2.72 (1.78–4.15) | <0.0001 | 2.28 (1.49–3.49) | 0.0002 |
|  | Other oncogenic type | 7666 | 486 | 0.07 (0.06–0.07) | 2.63 (2.16–3.20) | <0.0001 | 2.19 (1.79–2.67) | <0.0001 |
|  |  |  |  | *<0.0001* | *<0.0001* | | *<0.0001* | |
| Region | Europe | 7799 | 567 | 0.08 (0.07–0.08) | 1 | - | 1 | - |
|  | Asia Pacific | 3257 | 175 | 0.06 (0.05–0.07) | 0.71 (0.56–0.92) | 0.0090 | 1.19 (0.91–1.55) | 0.1965 |
|  | Latin America | 2991 | 163 | 0.05 (0.04–0.06) | 0.71 (0.54–0.93) | 0.0115 | 0.95 (0.72–1.24) | 0.6974 |
|  | North America | 2738 | 182 | 0.07 (0.06–0.08) | 0.91 (0.72–1.15) | 0.4208 | 1.22 (0.96–1.53) | 0.0990 |
|  |  |  |  | *<0.0001* | *0.0139* | | *0.1804* | |
| Tobacco exposure (number of pack- years) | None or less than 6 months (<0.5) | 9755 | 532 | 0.06 (0.05–0.06) | 1 | - | 1 | - |
|  | At least 6 months (≥0.5) | 6958 | 552 | 0.08 (0.08–0.09) | 1.49 (1.25–1.79) | <0.0001 | 1.26 (1.04–1.53) | 0.0183 |
|  |  |  |  | *<0.0001* | *<0.0001* | | *0.0183* | |
| Age at first sexual intercourse | ≥18 years or never had sexual intercourse | 4541 | 215 | 0.05 (0.04–0.06) | 1 | - | 1 | - |
|  | 15-17 years | 8982 | 604 | 0.07 (0.06–0.07) | 1.43 (1.14–1.79) | 0.0022 | 1.15 (0.93–1.44) | 0.2025 |
|  | <15 years | 3245 | 268 | 0.08 (0.07–0.09) | 1.76 (1.36–2.29) | <0.0001 | 1.19 (0.91–1.56) | 0.2132 |
|  |  |  |  | *<0.0001* | *<0.0001* | | *0.3791* | |
| Number of sexual partners during the past 12 months | 0–1 | 9116 | 513 | 0.06 (0.05–0.06) | 1 | - | 1 | - |
|  | 2-3 | 5624 | 393 | 0.07 (0.06–0.08) | 1.25 (1.05–1.49) | 0.0115 | 1.00 (0.85–1.19) | 0.9727 |
|  | ≥4 | 1970 | 171 | 0.09 (0.08–0.11) | 1.61 (1.24–2.10) | 0.0004 | 1.22 (0.94–1.57) | 0.1383 |
|  |  |  |  | *<0.0001* | *0.0006* | | *0.2994* | |
| History of *Chlamydia trachomatis* during the past 12 months | No | 14765 | 963 | 0.07 (0.06–0.07) | 1 | - | Not included | |
|  | Yes | 1919 | 112 | 0.06 (0.05–0.07) | 0.87 (0.68–1.13) | 0.3041 |  | |
|  |  |  |  | *0.1771* | *0.3041* | |  | |
| Marital/partner status | Single | 12747 | 840 | 0.07 (0.06–0.07) | 1 | - | Not included | |
|  | Living or lived with partner | 3819 | 231 | 0.07 (0.06–0.07) | 0.96 (0.78–1.17) | 0.6664 |  | |
|  |  |  |  | *0.5520* | *0.6664* | |  | |
| Previous pregnancy | No | 14961 | 936 | 0.07 (0.06–0.07) | 1 | - | 1 | - |
|  | Yes | 1804 | 151 | 0.08 (0.06–0.09) | 1.21 (0.97–1.50) | 0.0937 | 1.47 (1.18–1.84) | 0.0006 |
|  |  |  |  | *0.0325* | *0.0937* | | *0.0006* | |
| Use of hormones for contraception or other indication | No | 3918 | 197 | 0.05 (0.05–0.06) | 1 | - | 1 | - |
|  | Yes | 12767 | 878 | 0.07 (0.07–0.08) | 1.41 (1.16–1.72) | 0.0007 | 1.21 (1.00–1.48) | 0.0554 |
|  |  |  |  | *<0.0001* | *0.0007* | | *0.0554* | |
| Use of intrauterine device | No | 16152 | 1041 | 0.07 (0.06–0.07) | 1 | - | Not included | |
|  | Yes | 533 | 34 | 0.07 (0.05–0.10) | 0.96 (0.63–1.46) | 0.8475 |  | |
|  |  |  |  | *0.8088* | *0.8475* | |  | |
| Cervical HPV co-infections detected up to occurrence of lesion^3^ | No | 6899 | 278 |  | 1 | - | 1 | - |
|  | Yes (at least 1 oncogenic HPV type) | 8463 | 755 |  | 2.30 (1.92–2.75) | <0.0001 | 1.85 (1.54–2.21) | <0.0001 |
|  | Yes (only non-oncogenic HPV types) | 1423 | 54 |  | 1.10 (0.81–1.49) | 0.5378 | 0.99 (0.73–1.34) | 0.9366 |
|  |  |  |  | *Not done^3^* | *<0.0001* | | *<0.0001* | |
| Previous cervical HPV infection | No | 7663 | 427 | 0.05 (0.05–0.06) | 1 | - | 1 | - |
|  | Yes (at least 1 oncogenic HPV type) | 8306 | 617 | 0.08 (0.08–0.09) | 1.64 (1.42–1.89) | <0.0001 | 1.52 (1.31–1.77) | <0.0001 |
|  | Yes (only non-oncogenic HPV types) | 816 | 43 | 0.06 (0.04–0.08) | 1.05 (0.73–1.52) | 0.7912 | 1.22 (0.85–1.73) | 0.2791 |
|  |  |  |  | *<0.0001* | *<0.0001* | | *<0.0001* | |
| Previous CIN1+^4^ | No | 16195 | 1012 | 0.06 (0.06–0.07) | 1 | - | 1 | - |
|  | Yes (any oncogenic or non-oncogenic HPV type) | 590 | 75 | 0.13 (0.11–0.17) | 2.84 (2.01–4.03) | <0.0001 | 2.32 (1.59–3.40) | <0.0001 |
|  |  |  |  | *<0.0001* | *<0.0001* | | *<0.0001* | |
| Concomitant CIN1+^3,5^ | No | 15169 | 969 |  | 1 | - | 1 | - |
|  | Yes (with any oncogenic or non-oncogenic HPV type) | 1616 | 118 |  | 3.26 (2.56–4.16) | <0.0001 | 2.76 (2.10–3.63) | <0.0001 |
|  |  |  |  | *Not done^3^* | *<0.0001* | | *<0.0001* | |
|  |  |  |  |  |  | |  | |

^1^Covariates were included in the multivariable analysis if they had a global p-value of <0.2 in the univariate analysis (except region which was always included).

^2^Infections or lesions with a missing value for a covariate included in the analysis were excluded from the multivariable analysis. The analysis included 4789 women with 16580 infections and 1069 lesions.

^3^Time-varying covariates: Kaplan-Meier analysis not done.

Values in italics show the log-rank p-value (for the probability of developing a lesion) or the global p-value (for the univariate and multivariable analyses).

Analysis was performed in the control group of the TVC-E cohort, in women aged 15–25 years, in whom a cervical HPV infection was detected prior to the last study visit.

CIN: cervical intraepithelial neoplasia; HPV: human papillomavirus

**Table S6. Risk of progression of an HPV infection of any duration to a CIN2+ lesion associated with the same HPV type**

| **Determinant** | **Category** | **No. infections N=16785** | **No. infections with CIN2+**  **N=587** | **Probability of developing CIN2+ at month 24 (95% CI)** | **Univariate analysis** | | **Multivariable analysis^1,2^** | |
| --- | --- | --- | --- | --- | --- | --- | --- | --- |
|  |  |  |  |  | **Hazard ratio  (95% CI)** | **p-value** | **Hazard ratio  (95% CI)** | **p-value** |
| Duration of infection | Transient | 9434 | 106 | 0.01 (0.01–0.01) | 1 | - | 1 | - |
|  | Less than 6MPI | 449 | 13 | 0.03 (0.02–0.06) | 2.70 (1.44–5.07) | 0.0020 | 2.32 (1.22–4.40) | 0.0102 |
|  | 6MPI | 6902 | 468 | 0.07 (0.06–0.07) | 5.72 (4.56–7.18) | <0.0001 | 4.61 (3.66–5.81) | <0.0001 |
|  |  |  |  | *<0.0001* | *<0.0001* | | *<0.0001* | |
| HPV type | Non-oncogenic type | 4824 | 42 | 0.01 (0.01–0.01) | 1 | - | 1 | - |
|  | HPV-16 | 1557 | 175 | 0.10 (0.09–0.12) | 12.75 (9.39–17.31) | <0.0001 | 9.25 (6.84–12.51) | <0.0001 |
|  | HPV-18 | 945 | 37 | 0.04 (0.03–0.06) | 4.45 (3.01–6.58) | <0.0001 | 3.56 (2.40–5.27) | <0.0001 |
|  | HPV-31 | 914 | 54 | 0.06 (0.05–0.08) | 6.97 (4.90–9.92) | <0.0001 | 5.09 (3.56–7.29) | <0.0001 |
|  | HPV-33 | 488 | 50 | 0.11 (0.08–0.14) | 12.04 (8.35–17.38) | <0.0001 | 9.14 (6.34–13.18) | <0.0001 |
|  | HPV-45 | 391 | 14 | 0.03 (0.02–0.06) | 4.22 (2.40–7.43) | <0.0001 | 3.64 (2.08–6.40) | <0.0001 |
|  | Other oncogenic type | 7666 | 215 | 0.03 (0.03–0.03) | 3.20 (2.37–4.32) | <0.0001 | 2.63 (1.94–3.57) | <0.0001 |
|  |  |  |  | *<0.0001* | *<0.0001* | | *<0.0001* | |
| Region | Europe | 7799 | 285 | 0.04 (0.03–0.04) | 1 | - | 1 | - |
|  | Asia Pacific | 3257 | 99 | 0.03 (0.03–0.04) | 0.80 (0.57–1.12) | 0.1978 | 1.50 (1.01–2.21) | 0.0434 |
|  | Latin America | 2991 | 99 | 0.03 (0.03–0.04) | 0.85 (0.59–1.21) | 0.3590 | 1.26 (0.87–1.85) | 0.2257 |
|  | North America | 2738 | 104 | 0.04 (0.03–0.05) | 1.03 (0.75–1.41) | 0.8745 | 1.44 (1.03–2.02) | 0.0349 |
|  |  |  |  | *0.1395* | *0.4693* | | *0.1061* | |
| Tobacco exposure (number of pack- years) | None or less than 6 months (<0.5) | 9755 | 283 | 0.03 (0.03–0.03) | 1 | - | 1 | - |
|  | At least 6 months (≥0.5) | 6958 | 301 | 0.04 (0.04–0.05) | 1.53 (1.20–1.95) | 0.0005 | 1.28 (0.97–1.69) | 0.0813 |
|  |  |  |  | *<0.0001* | *0.0005* | | *0.0813* | |
| Age at first sexual intercourse | ≥18 years or never had sexual intercourse | 4541 | 111 | 0.03 (0.02–0.03) | 1 | - | 1 | - |
|  | 15-17 years | 8982 | 323 | 0.05 (0.04–0.06) | 1.47 (1.08–2.01) | 0.0143 | 1.22 (0.89–1.67) | 0.2189 |
|  | <15 years | 3245 | 153 | 0.04 (0.03–0.04) | 1.94 (1.36–2.75) | 0.0002 | 1.32 (0.89–1.95) | 0.1701 |
|  |  |  |  | *<0.0001* | *0.0011* | | *0.3527* | |
| Number of sexual partners during the past 12 months | 0–1 | 9116 | 293 | 0.03 (0.03–0.04) | 1 | - | 1 | - |
|  | 2-3 | 5624 | 192 | 0.03 (0.03–0.04) | 1.07 (0.84–1.35) | 0.5937 | 0.88 (0.69–1.11) | 0.2777 |
|  | ≥4 | 1970 | 97 | 0.05 (0.04–0.07) | 1.61 (1.15–2.25) | 0.0055 | 1.26 (0.89–1.79) | 0.1883 |
|  |  |  |  | *0.0002* | *0.0202* | | *0.1229* | |
| History of *Chlamydia trachomatis* during the past 12 months | No | 14765 | 513 | 0.04 (0.03–0.04) | 1 | - | Not included | |
|  | Yes | 1919 | 67 | 0.03 (0.03–0.04) | 0.98 (0.69–1.39) | 0.8950 |  | |
|  |  |  |  | *0.8549* | *0.8950* | |  | |
| Marital/partner status | Single | 12747 | 456 | 0.04 (0.03–0.04) | 1 | - | Not included | |
|  | Living or lived with partner | 3819 | 123 | 0.04 (0.03–0.04) | 0.95 (0.73–1.24) | 0.7109 |  | |
|  |  |  |  | *0.6173* | *0.7109* | |  | |
| Previous pregnancy | No | 14961 | 490 | 0.03 (0.03–0.04) | 1 | - | 1 | - |
|  | Yes | 1804 | 97 | 0.05 (0.04–0.06) | 1.44 (1.10–1.89) | 0.0085 | 1.60 (1.19–2.14) | 0.0018 |
|  |  |  |  | *0.0010* | *0.0085* | | *0.0018* | |
| Use of hormones for contraception or other indication | No | 3918 | 93 | 0.03 (0.02–0.03) | 1 | - | 1 | - |
|  | Yes | 12767 | 487 | 0.04 (0.04–0.04) | 1.67 (1.25–2.22) | 0.0004 | 1.45 (1.08–1.95) | 0.0143 |
|  |  |  |  | *<0.0001* | *0.0004* | | *0.0143* | |
| Use of intrauterine device | No | 16152 | 562 | 0.04 (0.03–0.04) | 1 | - | Not included | |
|  | Yes | 533 | 18 | 0.04 (0.02–0.06) | 0.93 (0.51–1.70) | 0.8111 |  | |
|  |  |  |  | *0.7593* | *0.8111* | |  | |
| Cervical HPV co-infections detected up to occurrence of lesion^3^ | No | 6932 | 143 |  | 1 | - | 1 | - |
|  | Yes (at least 1 oncogenic HPV type) | 8429 | 421 |  | 2.39 (1.89–3.03) | <0.0001 | 1.93 (1.52–2.45) | <0.0001 |
|  | Yes (only non-oncogenic HPV types) | 1424 | 23 |  | 0.87 (0.55–1.36) | 0.5315 | 0.76 (0.48–1.20) | 0.2375 |
|  |  |  |  | *Not done^3^* | *<0.0001* | | *<0.0001* | |
| Previous cervical HPV infection | No | 7662 | 259 | 0.03 (0.03–0.03) | 1 | - | 1 | - |
|  | Yes (at least 1 oncogenic HPV type) | 8307 | 304 | 0.04 (0.04–0.05) | 1.37 (1.13–1.68) | 0.0016 | 1.27 (1.03–1.57) | 0.0254 |
|  | Yes (only non-oncogenic HPV types) | 816 | 24 | 0.03 (0.02–0.05) | 0.99 (0.60–1.64) | 0.9755 | 1.21 (0.75–1.96) | 0.4324 |
|  |  |  |  | *0.0006* | *0.0049* | | *0.0803* | |
| Previous CIN1+^4^ | No | 16184 | 548 | 0.03 (0.03–0.04) | 1 | - | 1 | - |
|  | Yes (any oncogenic or non-oncogenic HPV type) | 601 | 39 | 0.08 (0.05–0.11) | 2.78 (1.78–4.33) | <0.0001 | 2.74 (1.73–4.32) | <0.0001 |
|  |  |  |  | *<0.0001* | *<0.0001* | | *<0.0001* | |
| Concomitant CIN1+^3,5^ | No | 14858 | 493 |  | 1 | - | 1 | - |
|  | Yes (with any oncogenic or non-oncogenic HPV type) | 1927 | 94 |  | 3.42 (2.53–4.63) | <0.0001 | 2.65 (1.89–3.71) | <0.0001 |
|  |  |  |  | *Not done^3^* | *<0.0001* | | *<0.0001* | |

^1^Covariates were included in the multivariable analysis if they had a global p-value of <0.2 in the univariate analysis (except region which was always included).

^2^Infections or lesions with a missing value for a covariate included in the analysis were excluded from the multivariable analysis. The analysis included 3337 women with 6835 6MPI and 818 lesions.

^3^Time-varying covariates: Kaplan-Meier analysis not done.

^4^CIN1+ associated with an HPV type different to the reference infection, preceding the onset of the 6MPI

^5^CIN1+ associated with an HPV type different to the reference infection, concomitant to the 6MPI (following its onset and preceding its end)

Values in italics show the log-rank p-value (for the probability of developing a lesion) or the global p-value (for the univariate and multivariable analyses).

6MPI: 6-month persistent infection; CIN: cervical intraepithelial neoplasia; HPV: human papillomavirus

**Table S7. Risk of progression of an HPV infection of any duration to a CIN3+ lesion associated with the same HPV type**

| **Determinant** | **Category** | **No. infections N=16785** | **No. infections with CIN3+**  **N=183** | **Probability of developing CIN3+ at month 24 (95% CI)** | **Univariate analysis** | | **Multivariable analysis^1,2^** | |
| --- | --- | --- | --- | --- | --- | --- | --- | --- |
|  |  |  |  |  | **Hazard ratio  (95% CI)** | **p-value** | **Hazard ratio  (95% CI)** | **p-value** |
| Duration of infection | Transient | 9434 | 25 | 0.00 (0.00–0.00) | 1 | - | 1 | - |
|  | Less than 6MPI | 449 | 2 | 0.00 (0.00–0.02) | 1.74 (0.40–7.59) | 0.4634 | 1.45 (0.32–6.48) | 0.6275 |
|  | 6MPI | 6902 | 156 | 0.02 (0.02–0.03) | 7.80 (4.90–12.42) | <0.0001 | 5.29 (3.34–8.38) | <0.0001 |
|  |  |  |  | *<0.0001* | *<0.0001* | | *<0.0001* | |
| HPV type | Non-oncogenic type | 4824 | 7 | 0.00 (0.00–0.00) | 1 | - | 1 | - |
|  | HPV-16 | 1557 | 75 | 0.04 (0.03–0.06) | 31.35 (14.89–66.02) | <0.0001 | 20.93 (9.97–43.95) | <0.0001 |
|  | HPV-18 | 945 | 9 | 0.01 (0.01–0.02) | 6.37 (2.37–17.10) | 0.0002 | 4.74 (1.78–12.58) | 0.0018 |
|  | HPV-31 | 914 | 15 | 0.02 (0.01–0.03) | 11.45 (5.09–25.78) | <0.0001 | 7.82 (3.46–17.63) | <0.0001 |
|  | HPV-33 | 488 | 21 | 0.05 (0.03–0.07) | 29.17 (13.62–62.50) | <0.0001 | 20.47 (9.45–44.35) | <0.0001 |
|  | HPV-45 | 391 | 3 | 0.00 (0.00–0.02) | 5.38 (1.40–20.75) | 0.0145 | 4.45 (1.17–16.97) | 0.0286 |
|  | Other oncogenic type | 7666 | 53 | 0.01 (0.01–0.01) | 4.66 (2.19–9.91) | <0.0001 | 3.51 (1.62–7.59) | 0.0014 |
|  |  |  |  | *<0.0001* | *<0.0001* | | *<0.0001* | |
| Region | Europe | 7799 | 79 | 0.01 (0.01–0.01) | 1 |  | 1 |  |
|  | Asia Pacific | 3257 | 34 | 0.01 (0.01–0.01) | 0.98 (0.55–1.73) | 0.9436 | 1.88 (0.89–3.94) | 0.0957 |
|  | Latin America | 2991 | 32 | 0.01 (0.01–0.01) | 0.96 (0.54–1.70) | 0.8934 | 1.60 (0.84–3.06) | 0.1545 |
|  | North America | 2738 | 38 | 0.01 (0.01–0.02) | 0.32 (0.79–2.21) | 0.2837 | 1.89 (1.07–3.34) | 0.0273 |
|  |  |  |  | *0.4467* | *0.6754* | | *0.1457* | |
| Tobacco exposure (number of pack- years) | None or less than 6 months (<0.5) | 9755 | 83 | 0.01 (0.01–0.01) | 1 |  | 1 |  |
|  | At least 6 months (≥0.5) | 6958 | 98 | 0.01 (0.01–0.02) | 1.70 (1.15–2.52) | 0.0080 | 1.40 (0.86–2.30) | 0.1777 |
|  |  |  |  | *0.0003* | *0.0080* | | *0.1777* | |
| Age at first sexual intercourse | ≥18 years or never had sexual intercourse | 4541 | 35 | 0.01 (0.01–0.01) | 1 |  | 1 |  |
|  | 15-17 years | 8982 | 82 | 0.01 (0.01–0.01) | 1.17 (0.69–1.99) | 0.5604 | 0.96 (0.54–1.70) | 0.8943 |
|  | <15 years | 3245 | 66 | 0.02 (0.02–0.03) | 2.62 (1.49–4.60) | 0.0008 | 1.70 (0.87–3.33) | 0.1192 |
|  |  |  |  | *<0.0001* |  | | *0.0637* | |
| Number of sexual partners during the past 12 months | 0–1 | 9116 | 99 | 0.01 (0.01–0.01) | 1 |  | Not included | |
|  | 2-3 | 5624 | 56 | 0.01 (0.01–0.01) | 0.92 (0.61–1.38) | 0.6785 |  |  |
|  | ≥4 | 1970 | 28 | 0.02 (0.01–0.03) | 1.38 (0.82–2.32) | 0.2245 |  |  |
|  |  |  |  | *0.1973* | *0.3216* | |  | |
| History of *Chlamydia trachomatis* during the past 12 months | No | 14765 | 161 | 0.01 (0.01–0.01) | 1 |  | Not included | |
|  | Yes | 1919 | 21 | 0.01 (0.01–0.02) | 0.95 (0.54–1.69) | 0.8697 |  | |
|  |  |  |  | *0.8329* | *0.8697* | |  | |
| Marital/partner status | Single | 12747 | 134 | 0.01 (0.01–0.01) | 1 |  | Not included | |
|  | Living or lived with partner | 3819 | 47 | 0.01 (0.01–0.02) | 1.26 (0.84–1.89) | 0.2671 |  | |
|  |  |  |  | *0.1731* | *0.2671* | |  | |
| Previous pregnancy | No | 14961 | 135 | 0.01 (0.01–0.01) | 1 |  | 1 |  |
|  | Yes | 1804 | 48 | 0.02 (0.01–0.03) | 2.40 (1.65–3.48) | <0.0001 | 2.35 (1.53–3.61) | <0.0001 |
|  |  |  |  | *<0.0001* | *<0.0001* | | *<0.0001* | |
| Use of hormones for contraception or other indication | No | 3918 | 33 | 0.01 (0.01–0.01) | 1 |  | 1 |  |
|  | Yes | 12767 | 149 | 0.01 (0.01–0.01) | 1.45 (0.89-2.36) | 0.1355 | 1.21 (0.70–2.09) | 0.4957 |
|  |  |  |  | *0.0524* | *0.1355* | | *0.4957* | |
| Use of intrauterine device | No | 16152 | 173 | 0.01 (0.01–0.01) | 1 |  | Not included | |
|  | Yes | 533 | 9 | 0.02 (0.01–0.04) | 1.48 (0.62–3.53) | 0.3720 |  | |
|  |  |  |  | *0.2450* | *0.3720* | |  | |
| Cervical HPV co-infections detected up to occurrence of lesion^3^ | No | 7035 | 53 |  | 1 |  | 1 |  |
|  | Yes (at least 1 oncogenic HPV type) | 8310 | 122 | *Not done^3^* | 1.83 (1.26–2.65) | 0.0016 | 1.40 (0.99–1.99) | 0.0595 |
|  | Yes (only non-oncogenic HPV types) | 1440 | 8 |  | 0.76 (0.36–1.62) | 0.4795 | 0.60 (0.27–1.33) | 0.2110 |
|  |  |  |  |  | *0.0011* | | *0.0243* | |
| Previous cervical HPV infection | No | 7662 | 93 | 0.01 (0.01–0.01) | 1 |  | Not included | |
|  | Yes (at least 1 oncogenic HPV type) | 8307 | 78 | 0.01 (0.01–0.01) | 1.09 (0.77–1.55) | 0.6151 |  |  |
|  | Yes (only non-oncogenic HPV types) | 816 | 12 | 0.01 (0.01–0.03) | 1.51 (0.68–3.35) | 0.3148 |  |  |
|  |  |  |  | *0.3952* | *0.5712* | |  | |
| Previous CIN1+^4^ | No | 16183 | 171 | 0.01 (0.01–0.01) | 1 |  | 1 |  |
|  | Yes (any oncogenic or non-oncogenic HPV type) | 602 | 12 | 0.02 (0.01–0.04) | 3.07 (1.56–6.04) | 0.0012 | 3.65 (1.76–7.54) | 0.0005 |
|  |  |  |  | *<0.0001* | *0.0012* | | *0.0005* | |
| Concomitant CIN1+^3,5^ | No | 14609 | 124 | *Not done^3^* | 1 |  | 1 |  |
|  | Yes (with any oncogenic or non-oncogenic HPV type) | 2176 | 59 |  | 6.12 (3.78–9.91) | <0.0001 | 4.70 (2.77–7.99) | <0.0001 |
|  |  |  |  |  | *<0.0001* | | *<0.0001* | |

^1^Covariates were included in the multivariable analysis if they had a global p-value of <0.2 in the univariate analysis (except region which was always included).

^2^Infections or lesions with a missing value for a covariate included in the analysis were excluded from the multivariable analysis. The analysis included 3337 women with 6835 6MPI and 818 lesions.

^3^Time-varying covariates: Kaplan-Meier analysis not done.

^4^CIN1+ associated with an HPV type different to the reference infection, preceding the onset of the 6MPI

^5^CIN1+ associated with an HPV type different to the reference infection, concomitant to the 6MPI (following its onset and preceding its end)

Values in italics show the log-rank p-value (for the probability of developing a lesion) or the global p-value (for the univariate and multivariable analyses).

6MPI: 6-month persistent infection; CIN: cervical intraepithelial neoplasia; HPV: human papillomavirus

**Table S8. Clearance and median duration of a cervical HPV infection**

| **Determinant** | **Category** | **No. infections N=16785** | **No. cleared infections**  **N=10983** | | **Median duration, months (interquartiles)** | **Univariate analysis** | | **Multivariable analysis^1,2^** | |
| --- | --- | --- | --- | --- | --- | --- | --- | --- | --- |
|  |  |  |  | |  | **Hazard ratio  (95% CI)** | **p-value** | **Hazard ratio  (95% CI)** | **p-value** |
| Duration of infection | Transient | 9434 | 7102 | 6.26 (5.70–8.16) | | 1 | - | 1 | - |
|  | Less than 6MPI | 449 | 287 | 11.02 (10.16–13.18) | | 0.41 (0.35–0.46) | <0.0001 | 0.40 (0.34–0.45) | <0.0001 |
|  | 6MPI | 6902 | 3594 | 18.85 (12.66–34.52) | | 0.14 (0.14–0.15) | <0.0001 | 0.14 (0.13–0.14) | <0.0001 |
|  |  |  |  | *<0.0001* | | *<0.0001* | | *<0.0001* | |
| HPV type | Non-oncogenic type | 4824 | 3291 | 8.26 (5.97–17.57) | | 1 | - | 1 | - |
|  | HPV-16 | 1557 | 920 | 17.11 (7.80–30.26) | | 0.53 (0.50–0.57) | <0.0001 | 0.81 (0.75–0.88) | <0.0001 |
|  | HPV-18 | 945 | 615 | 11.84 (6.20–23.11) | | 0.76 (0.69–0.83) | <0.0001 | 0.93 (0.85–1.03) | 0.1748 |
|  | HPV-31 | 914 | 519 | 13.80 (6.43–28.89) | | 0.61 (0.56–0.67) | <0.0001 | 0.82 (0.74–0.90) | <0.0001 |
|  | HPV-33 | 488 | 320 | 12.00 (6.20–21.90) | | 0.76 (0.67–0.85) | <0.0001 | 0.95 (0.84–1.08) | 0.4557 |
|  | HPV-45 | 391 | 246 | 11.48 (6.20–23.31) | | 0.80 (0.70–0.92) | 0.0013 | 0.93 (0.80–1.08) | 0.3465 |
|  | Other oncogenic type | 7666 | 5072 | 11.77 (6.20–20.03) | | 0.80 (0.76–0.84) | <0.0001 | 0.96 (0.91–1.02) | 0.1739 |
|  |  |  |  | *<0.0001* | | *<0.0001* | | *<0.0001* | |
| Region | Europe | 7799 | 5012 | 11.84 (6.20–20.03) | | 1 | - | 1 | - |
|  | Asia Pacific | 3257 | 2154 | 11.44 (6.13–21.70) | | 1.02 (0.96–1.08) | 0.5428 | 0.98 (0.90–1.07) | 0.6727 |
|  | Latin America | 2991 | 2040 | 11.18 (6.07–22.03) | | 1.03 (0.97–1.10) | 0.3307 | 0.95 (0.87–1.04) | 0.2748 |
|  | North America | 2738 | 1777 | 11.70 (6.13–20.59) | | 1.02 (0.96–1.09) | 0.5696 | 0.88 (0.81–0.96) | 0.0050 |
|  |  |  |  | *0.6488* | | *0.7709* | | *0.0401* | |
| Tobacco exposure(number of pack years) | None or less than 6 months (<0.5) | 9755 | 6438 | 11.48 (6.13–20.72) | | 1 | - | Not included | |
|  | At least 6 months (≥0.5) | 6958 | 4486 | 11.80 (6.20–20.92) | | 0.97 (0.93–1.02) | 0.2645 |  |  |
|  |  |  |  | *0.1858* | | *0.2645* | |  | |
| Age at first sexual intercourse | ≥18 years or never had sexual intercourse | 4541 | 2927 | 11.51 (6.10–22.39) | | 1 | - | Not included | |
|  | 15-17 years | 8982 | 5897 | 11.67 (6.16–20.62) | | 1.02 (0.97–1.08) | 0.4810 |  |  |
|  | <15 years | 3245 | 2148 | 11.77 (6.20–19.77) | | 1.01 (0.95–1.08) | 0.6850 |  |  |
|  |  |  |  | *0.6946* | | *0.7798* | |  | |
| Number of sexual partners during the past 12 months | 0–1 | 9116 | 5933 | 11.61 (6.10–22.26) | | 1 | - | 1 | - |
|  | 2-3 | 5624 | 3722 | 11.70 (6.20–20.00) | | 1.03 (0.98–1.08) | 0.2669 | 1.01 (0.95–1.09) | 0.6901 |
|  | ≥4 | 1970 | 1293 | 11.48 (6.20–18.36) | | 1.09 (1.02–1.17) | 0.0118 | 1.02 (0.91–1.13) | 0.7761 |
|  |  |  |  | *0.014* | | *0.0381* | | *0.9117* | |
| History of *Chlamydia trachomatis* during the past 12 months | No | 14765 | 9628 | 11.70 (6.16–20.89) | | 1 | - | 1 | - |
|  | Yes | 1919 | 1314 | 11.48 (6.16–20.23) | | 1.04 (0.98–1.12) | 0.1997 | 1.02 (0.93–1.12) | 0.6727 |
|  |  |  |  | *0.1440* | | *0.1997* | | *0.6727* | |
| Marital/partner status | Single | 12747 | 8526 | 11.48 (6.16–19.97) | | 1 | - | 1 | - |
|  | Living or lived with partner | 3819 | 2313 | 12.07 (6.20–23.93) | | 0.90 (0.85–0.95) | <0.0001 | 0.79 (0.73–0.86) | <0.0001 |
|  |  |  |  | *<0.0001* | | *<0.0001* | | *<0.0001* | |
| Previous pregnancy | No | 14961 | 9636 | 11.70 (6.20–20.59) | | 1 | - | Not included | |
|  | Yes | 1804 | 1331 | 11.25 (6.00–22.07) | | 1.03 (0.97–1.10) | 0.3279 |  |  |
|  |  |  |  | *0.2886* | | *0.3279* | |  | |
| Use of hormones for contraception or other indication | No | 3918 | 2608 | 11.70 (6.16–21.11) | | 1 | - | Not included | |
|  | Yes | 12767 | 8334 | 11.61 (6.16–20.59) | | 1.01 (0.96–1.06) | 0.8080 |  |  |
|  |  |  |  | *0.7733* | | *0.8080* | |  | |
| Use of intrauterine device | No | 16152 | 10588 | 11.64 (6.16–20.59) | | 1 | - | Not included | |
|  | Yes | 533 | 354 | 12.00 (6.13–24.26) | | 0.93 (0.83–1.05) | 0.2282 |  | |
|  |  |  |  | *0.1773* | | *0.2282* | |  | |
| Cervical HPV co-infections detected up to occurrence of lesion^3^ | No | 5282 | 3433 |  | | 1 | - | 1 | - |
|  | Yes (at least 1 oncogenic HPV type) | 10099 | 6638 |  | | 1.06 (1.01–1.11) | 0.0169 | 1.08 (1.01–1.14) | 0.0146 |
|  | Yes (only non-oncogenic HPV types) | 1404 | 912 |  | | 1.05 (0.98–1.14) | 0.1741 | 1.03 (0.93–1.15) | 0.5501 |
|  |  |  |  | *Not done^3^* | | *0.0512* | | *0.0473* | |
| Previous cervical HPV infection | No | 7662 | 5694 | 11.25 (6.03–18.79) | | 1 | - | 1 | - |
|  | Yes (same HPV type) | 1134 | 521 | 13.64 (6.43–NE) | | 0.66 (0.60–0.73) | <0.0001 | 0.58 (0.52–0.64) | <0.0001 |
|  | Yes (other HPV type[s]) | 7989 | 4768 | 11.84 (6.23–22.43) | | 0.88 (0.84–0.92) | <0.0001 | 0.72 (0.68–0.77) | <0.0001 |
|  |  |  |  | *<0.0001* | | *<0.0001* | | *<0.0001* | |

^1^Covariates were included in the multivariable analysis if they had a global p-value of <0.2 in the univariate analysis (except region which was always included).

^2^Infections or clearances with a missing value for a covariate included in the analysis were excluded from the multivariable analysis. The analysis included 4793 women with 16505 infections and 10810 clearances.

^3^Time-varying covariate: Kaplan-Meier analysis not done.

Values in italics show the log-rank p-value (for the median duration of infection) or the global p-value (for the univariate and multivariable analyses).

Analysis was performed in the control group of the TVC-E cohort, in women aged 15–25 years, in whom a cervical HPV infection was detected prior to the last study visit.

HPV: human papillomavirus; NE: not estimated
