# Supplementary material for: Natural History of Progression of HPV Infection to Cervical Lesion or Clearance: Analysis of the Control Arm of the Large, Randomised PATRICIA Study
Source: PLoS One. 2013 Nov 19;8(11):e79260. doi: 10.1371/journal.pone.0079260 (PMC3834039; doi:10.1371/journal.pone.0079260)
Supplement: File S2 — Protocol and STROBE documents. (ZIP) [file pone.0079260.s002.zip › U_Jaisamrarn_et_al_SAP_EPI-HPV-032_BOD_(114480).pdf]

GlaxoSmithKline Biologicals

Avenue Fleming, 20

1300 Wavre, Belgium

Confidential & Proprietary Information

STATISTICAL ANALYSIS PLAN (SAP)

HPV NATURAL HISTORY: AN EPIDEMIOLOGICAL DATABASE ANALYSIS BASED ON DATA FROM THE CONTROL ARM SUBJECTS OF THE HPV-008 (580299/008) STUDY

PART 2: PROGRESSION FROM HPV INFECTION TO LESION

**Date of RAP approval**	Final - 11 October 2011
**Co-ordinating author**	Alice Raillard - Project Statistician

Details of study used as data source:

**Title**	Use of control arm data from the phase III, double-blind, randomized, controlled, multi-center study of GSK HPV vaccine to study the progression from HPV infection to lesion.
**eTrack study number**	114480
**eTrack abbreviated title**	EPI-HPV-032 BOD DB
**Study vaccine**	-

GlaxoSmithKline Biologicals

Clinical Research & Development

STATISTICAL ANALYSIS PLAN

HPV NATURAL HISTORY: AN EPIDEMIOLOGICAL DATABASE ANALYSIS BASED ON DATA FROM THE CONTROL ARM SUBJECTS OFTHE HPV-008 (580299/008) STUDY

PART 2: PROGRESSION FROM HPV INFECTION TO LESION

Approval

Date of RAP approval Final - 11 October 2011

Details of study used as data source:

**Title:**	Use of control arm data from the phase III, double-blind, randomized, controlled, multi-center study of GSK HPV vaccine to study the progression from HPV infection to lesion.
**E-Track number (alias):**	114480 (EPI-HPV-032 BOD DB)

Co-ordinating authors:

Sylvie di Nicola (Inferential), Statistician			
	Name	Signature	dd-mmm-yyyy
Marie-Cécile Bozonnat (4Clinics), Statistician			
	Name	Signature	dd-mmm-yyyy
Alice Raillard (4Clinics), Statistician			
	Name	Signature	dd-mmm-yyyy

Approved by:

Laurence Baril, Director, Central Epidemiology			
	Name	Signature	dd-mmm-yyyy
Dominique Rosillon, Project statistician, Central Epidemiology			
	Name	Signature	dd-mmm-yyyy
Frank Struyf, Senior Manager Lead CDM - Cervarix			
	Name	Signature	dd-mmm-yyyy

TABLE OF CONTENTS

PAGE

1 CONTEXT OF THE RAP DEVELOPMENT 6

2 DETAILED PLAN OF ANALYSES 7
- 2.1 Primary objective 7
- 2.2 Secondary objectives 7

3 HPV-008 DATA TO BE USED FOR ANALYSES 8
- 3.1 Summary of the HPV-008 study design 8
- 3.2 Study procedures resulting in data to be used for analyses 9

4 DATA EVALUATION 10
- 4.1 Endpoints 10
  - 4.1.1 Primary endpoint 10
  - 4.1.2 Secondary endpoints 10
- 4.2 Study cohorts 10
- 4.3 Case definitions associated with endpoints 11
  - 4.3.1 Cervical HPV infections 11
  - 4.3.2 Histopathological CIN associated with HPV type 12
- 4.4 Determinant definitions 13
  - 4.4.1 Reference cervical HPV infections used for definitions 13
  - 4.4.2 Clinical determinants 13
  - 4.4.3 Behavioral determinants 14
  - 4.4.4 Other determinants 15
- 4.5 Handling of missing data and inconsistencies 15
  - 4.5.1 Missing HPV type at visits 15
  - 4.5.2 Other missing data 16

5 CHANGES IN THE CONDUCT OF THE STUDY OR PLANNED ANALYSES 16

6 STATISTICAL ANALYSES 16
- 6.1 Statistical considerations 16
  - 6.1.1 General considerations 16
  - 6.1.2 Univariate and multivariable analyses 16
- 6.2 Statistical methods 17
  - 6.2.1 Description of study cohorts 17
  - 6.2.2 Subject characteristics 17
  - 6.2.3 Description of endpoints and determinants 18
    - 6.2.3.1 Endpoints and clinical determinants 18
    - 6.2.3.2 Behavioral determinants 18
  - 6.2.4 Analysis of time between persistent cervical HPV infections and lesions 18
    - 6.2.4.1 Population 18
    - 6.2.4.2 Analysis unit 18
    - 6.2.4.3 Event variable 19
    - 6.2.4.4 Time computation 20
    - 6.2.4.5 Statistical methods 21
  - 6.2.5 Analysis of time between any cervical HPV infections and lesions 22
    - 6.2.5.1 Population 22
    - 6.2.5.2 Analysis unit 23
    - 6.2.5.3 Event variable 23
    - 6.2.5.4 Time computation 24

6.2.5.5 Statistical methods 26
6.2.6 Analysis of clearance of cervical HPV infections..... 26
6.2.6.1 Population..... 26
6.2.6.2 Analysis unit 26
6.2.6.3 Dependent variable 26
6.2.6.4 Statistical methods 27
6.2.7 Analysis of time to first detected cervical HPV infections..... 27
6.2.7.1 Population..... 27
6.2.7.2 Analysis unit 27
6.2.7.3 Event variable..... 28
6.2.7.4 Time computation..... 28
6.2.7.5 Statistical methods 29
6.2.8 Complementary analyses..... 29

7 SEQUENCE OF ANALYSIS..... 30

8 REFERENCES..... 30

9 ANNEXES 32
9.1 Flowchart of study cohorts..... 32
9.2 Flowchart of cervical HPV infection endpoint determination 33
9.3 Flowcharts of data according to the objectives..... 35
9.3.1 Analysis of time between persistent cervical HPV infections and lesions 35
9.3.2 Analysis of time between any cervical HPV infections and lesions 36
9.3.3 Analysis of clearance of cervical HPV infections..... 37
9.3.4 Analysis of time to first detected cervical HPV infections..... 38
9.4 Determination of events and time to event..... 39
9.4.1 Analysis of time between persistent cervical HPV infections and lesions 39
9.4.2 Analysis of time between any cervical HPV infections and lesions 40
9.4.3 Analysis of time to first detected cervical HPV infections..... 41
9.5 Determinants taken into account in the analyses..... 42
9.5.1 Analysis of time between persistent (or any) cervical HPV infections and lesions..... 42
9.5.2 Analysis of clearance of cervical HPV infections..... 44
9.5.3 Analysis of time to first detected incident cervical HPV infections 46
9.5.4 Analysis of time to first confirmed 6-month persistent cervical HPV infections..... 47

LIST OF FIGURES

PAGE

Figure 1 Study Overview..... 8

LIST OF ABBREVIATIONS

AGC	Atypical glandular cells
$Al(OH)_3$	Aluminum hydroxide
AS04	GlaxoSmithKline's proprietary adjuvant system consisting of aluminium salt plus 3-*O*-desacyl-*4'*-monophosphoryl lipid A (MPL)
ASC-H	Atypical squamous cells, cannot exclude HSIL
ASC-US	Atypical squamous cells of undetermined significance
ATP	According-to-protocol (or protocol defined)
CI	Confidence Interval
CIN	Cervical intraepithelial neoplasia
CIN1	Cervical intraepithelial neoplasia as CIN1 adenocarcinoma in-situ and invasice cervical cancer
CIN2+	Cervical intraepithelial neoplasia as CIN2, CIN3 adenocarcinoma in-situ and invasice cervical cancer
CRF	Case Report Form
DNA	Deoxyribonucleic acid
ELISA	Enzyme-linked immunosorbent assay
GSK	GlaxoSmithKline
HAV	Hepatitis A virus
HPV	Human papillomavirus
HSIL	High grade squamous intraepithelial lesion
LBC	Liquid Based Cytology
LSIL	Low grade squamous intraepithelial lesion
MPL®	3-deacylated monophosphoryl lipid A
PCR	Polymerase chain reaction
SAP	Statistical analysis plan
SBIR	Simply the Best Internet Randomization
TAA	Type assignment algorithm
TVC	Total vaccinated cohort
TVC-Control arm	Total vaccinated cohort for control arm
TVC-1	Total vaccinated cohort for efficacy 1
TVC-1-Control arm	Total vaccinated cohort 1 for control arm
VLP	Virus-like particle

1 CONTEXT OF THE RAP DEVELOPMENT

This statistical analysis plan (SAP) describes the epidemiological approach used to investigate the progression from an initial cervical HPV infection to cervical pre-cancer and cancer. As most HPV infections are cleared by the immune system and do not result in clinical complications such as cervical pre-cancer and cancer, the clearance of HPV infections will be also investigated. In addition, the time to first HPV infection and to first persistent HPV infection will be explored.

Thus, the study objectives are to describe the following outcomes:

i) the time between confirmed persistent cervical HPV infections and the development of CIN associated with the same HPV type,

ii) the time between any cervical HPV infections and the development of CIN associated with the same HPV type,

iii) the clearance of cervical HPV infections,

iv) the time between the start of sexual activity and a first incident or a first persistent cervical HPV infection,

and to assess the effect on these outcomes of determinants such as sexual behavior, smoking history etc. known to predispose subjects to cervical HPV infections and/or clinical complications of HPV infections such as pre-cancer and cervical cancer.

The analyses will be performed using data obtained from the subjects included in the control arm of the HPV-008 study (End-of-Study (M48) database from the Phase III pivotal study to evaluate the efficacy of HPV-16/18 VLP AS04 vaccine compared with control HAV vaccine).

More specifically, analyses will include the use of the following data collected at enrolment and during all the visits over the 48-month follow-up of the control arm subjects included in the HPV-008 study:

i) virological (cervical HPV infection detected by PCR in cervical liquid-based cytology (LBC) samples),

ii) cyto-pathological examinations on LBC samples and histology samples (colposcopy performed according to the clinical management algorithms defined for the HPV-008 study),

iii) HPV-16/18 serostatus (by ELISA) at enrolment,

iv) socio-demographic and behavioral data.

Of note, the results of the analysis of the time between the start of sexual activity and a first incident or a first persistent cervical HPV infection will be presented in a stand-alone statistical report.

2 DETAILED PLAN OF ANALYSES

2.1 Primary objective

- To describe and evaluate the effect of clinical and behavioral determinants on the time between onset of **confirmed 6-month persistent cervical HPV infections** and detection of histopathologically-confirmed **CIN2+** associated with the same HPV type.

2.2 Secondary objectives

- To describe and evaluate the effect of clinical and behavioral determinants on the time between onset of **confirmed 6-month persistent cervical HPV infections** and detection of histopathologically-confirmed **CIN1+** associated with the same HPV type,

- To describe and evaluate the effect of clinical and behavioral determinants on the time between onset of **confirmed 6-month persistent cervical HPV infections** and detection of histopathologically-confirmed **CIN3+** associated with the same HPV type,

- To describe and evaluate the effect of clinical and behavioral determinants on the time between onset of **any cervical HPV infections** and detection of histopathologically-confirmed **CIN2+** associated with the same HPV type,

- To describe and evaluate the effect of clinical and behavioral determinants on the time between onset of **any cervical HPV infections** and detection of histopathologically-confirmed **CIN1+** associated with the same HPV type,

- To describe and evaluate the effect of clinical and behavioral determinants on the time between onset of **any cervical HPV infections** and detection of histopathologically-confirmed **CIN3+** associated with the same HPV type,

- To describe and evaluate the effect of clinical and behavioral determinants on the **clearance of cervical HPV infections**,

- To describe and evaluate the effect of clinical and behavioral determinants on the time between the **start of sexual activity** and **first detected incident cervical HPV infection**,

- To described and evaluate the effect of clinical and behavioral determinants on the time between the **start of sexual activity** and onset of **first confirmed 6-month persistent cervical HPV infection**.

The term "detected infection" is used instead of "new infection" as it is not possible to differentiate newly acquired cervical HPV infections from reactivation of previously acquired cervical HPV infections.

3 HPV-008 DATA TO BE USED FOR ANALYSES

3.1 Summary of the HPV-008 study design

This section describes the overall design of this HPV-008 efficacy trial. Please refer to the following documents for more details:

- Clinical study report (Development Phase III – BB-IND 7920) for the HPV-008 study dated February 2009,
- Amended final CSR dated May 2010,
- End-of-study report (Month 48).

Figure 1 Study Overview

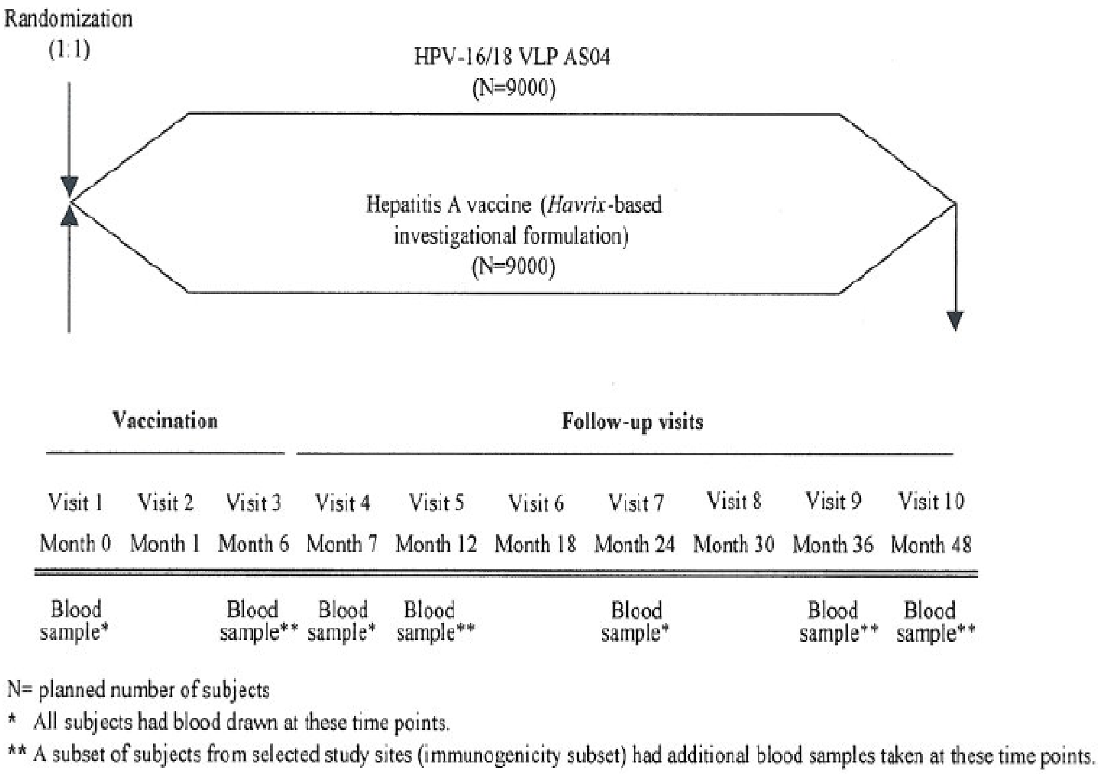

- **Experimental design:** A phase III, controlled, multi-center (n=135), and multi-country (n=14) study with two parallel groups.

- **Treatment Groups:** 2 groups: vaccine and control
  - Vaccine: 20µg HPV-16 L1 VLP/20µg and HPV-18 L1 VLP/50µg MPL®/ 500µg aluminum in the form of $Al(OH)_3$,
  - Control: Hepatitis A vaccine (Havrix™-based investigational formulation) (720 ELISA units of antigen; 500µg aluminum in the form of $Al(OH)_3$).

- **Treatment allocation:** Randomized (1:1).

- **Randomization method:** SBIR (Internet randomization).
- **Blinding**: Double-blind.
- **Enrolment period**: May 2004 to June 2005.
- **Study regions:** Asia Pacific, Europe, Latin America and North America.
- **Enrolled**: 18729 subjects aged 15-25 years. Total Vaccinated Cohort (TVC): 18644 subjects. Total Vaccinated Cohort for efficacy 1 (TVC-1): 18525 subjects.
- **Control arm** (HAV vaccine group)**:** TVC: 9325 subjects. TVC-1: 9267 subjects.
- **Vaccination schedule(s):** Three doses of HPV vaccine or HAV vaccine (control arm) administered on a Month 0, 1, 6 schedule.
- **Study visits:** 10 study visits per subject scheduled at Months 0, 1, 6, 7, 12, 18, 24, 30, 36 and 48.
- **Total duration of the study:** 48 months of follow-up from study entry planned for all subjects.
- **Database freeze for the final analysis:** October 2008; **Database freeze for the M48 analysis:** March 2010.

3.2 Study procedures resulting in data to be used for analyses

- Blood samples for all subjects were drawn at Months 0, 7 and 24 and in a subset of subjects from selected study sites.
- Gynecological examinations were performed at Months 0, 12, 24, 36 and 48.
- Cervical liquid-based cytology samples were collected at Months 0, 6, 12, 18, 24, 30, 36 and 48:
  - HPV DNA typing by PCR was performed on cervical liquid-based cytology samples at Months 0, 6, 12, 18, 24, 30, 36 and 48,
  - Cytopathological examinations were performed on liquid-based cytology samples to detect abnormal cells at Months 0, 12, 24, 36 and 48 in all subjects,
  - Screening for *Chlamydia trachomatis* and *Neisseria gonorrhoeae* on liquid-based cytology samples at Months 0, 12, 24, 36 and 48.

    Note: As of Protocol Amendment 2, screening for *Neisseria gonorrhoeae* was done only if considered appropriate by the investigator.
- Colposcopic referral according to appropriate clinical management algorithms. A central laboratory processed and interpreted results from liquid-based cytology and histology samples. All CIN endpoints were confirmed by an expert histopathology review panel that was blinded to vaccine status, HPV DNA status before biopsy and cytology reports. An Endpoint Committee was responsible for reviewing all available clinical and laboratory data prior to study unblinding by the external statistician to make final case assignments for all subjects assumed to meet criteria for primary and secondary efficacy endpoints.

- Behavioral questionnaire completed by interview for all subjects at Months 1, 12, 24, 36 and 48. The behavioral questionnaire (self-administered) collected specific socio-demographic and behavioral data concerning determinants that are recognized to predispose subjects to cervical HPV infection or are recognized co-factors for cervical carcinogenesis. The baseline questionnaire assessed at Month 1 collected data which occurred during past history and during the previous year. Questionnaires assessed at Month 12, 24, 36 and 48 collected data assessed during the previous year.

4 DATA EVALUATION

4.1 Endpoints

4.1.1 Primary endpoint

- Histopathologically-confirmed **CIN2+** associated with the same HPV type as a previous confirmed **persistent cervical HPV infection (6-month definition)**.

4.1.2 Secondary endpoints

- Histopathologically-confirmed **CIN1+** associated with the same HPV type as a previous **confirmed persistent cervical HPV infection (6-month definition)**.

- Histopathologically-confirmed **CIN3+** associated with the same HPV type as a previous **confirmed persistent cervical HPV infection (6-month definition)**.

- Histopathologically-confirmed **CIN2+** associated with the same HPV type as a previous cervical HPV infection **(any cervical HPV infection)**.

- Histopathologically-confirmed **CIN1+** associated with the same HPV type as a previous cervical HPV infection **(any cervical HPV infection)**.

- Histopathologically-confirmed **CIN3+** associated with the same HPV type as a previous cervical HPV infection **(any cervical HPV infection)**.

- **Clearance** of cervical HPV infections,

- **First detected incident cervical HPV infection** following the start of sexual activity,

- **First confirmed persistent cervical HPV infection (6-month definition)** following the start of sexual activity.

The case definitions associated with the endpoints (i.e. for the determination of incident, transient, persistent and other cervical HPV infections and histopathologically-confirmed CIN associated with a given HPV type) can be found in Sections 4.3.1 and 4.3.2.

4.2 Study cohorts

The Total vaccinated cohort 1 for Control arm (TVC-1-Control arm) will be used for the analyses. The TVC-1-Control arm will include all vaccinated subjects (i.e. who received at

least one vaccine dose), who were randomized to the control arm of HPV-008 study, for whom data are available for analysis of endpoints and who have a normal or low-grade cytology (i.e. negative or ASC-US or LSIL) at Month 0.

In previous analyses using the TVC-1-Control arm, subjects also had to be negative for HPV DNA (by PCR) at Month 0 for the corresponding HPV type in the analysis (i.e. HPV type associated with the endpoint). In the current statistical analysis plan, some of the analyses performed on subjects from the TVC-1-Control arm will take into account subjects who tested positive for HPV DNA at Month 0 or for whom PCR result is not available at Month 0.

A flowchart of the study cohorts can be found in Section 8.1.

4.3 Case definitions associated with endpoints

Incident, transient, persistent and other cervical HPV infections as well as histopathological endpoints will be defined according to the rules described in both the HPV-008 study protocol and the RAP for efficacy analyses (Amendment 2 dated 8 August 2008).

4.3.1 Cervical HPV infections

- **Incident cervical HPV infection**: an incident cervical infection with a given HPV type is defined as the new detection of this HPV type (by PCR) at any time point during the follow-up period (i.e. after enrolment in the control arm of study HPV-008).

- **Transient cervical HPV infection**: a transient cervical infection with a given HPV type is defined as the detection of this HPV type (by PCR) at any single time point during the follow-up period followed by a negative sample for that HPV type at the evaluation performed 6 months after the evaluation when the cervical HPV infection was first detected.

- **6-month persistent cervical HPV infection**: a persistent cervical HPV infection (6-month definition) is defined as the detection of the same HPV type (by PCR) in cervical samples at two consecutive evaluations over approximately a 6-month interval. Thus, the algorithmic definition of a 6-month persistent infection is that there exists a sequence of positive samples with the same HPV type, not interrupted by negative samples, such that the total range is more than 5 months (> 150 days). The start of the persistent cervical HPV infection is defined as the date of the first positive sample in the sequence.

- **12-month persistent cervical HPV infection**: a persistent cervical HPV infection (12-month definition) is defined as the detection of the same HPV type (by PCR) at all available time points over approximately a 12-month interval. Thus, the algorithmic definition of a 12-month persistent cervical infection is that there exists a sequence of positive samples with the same HPV type, not interrupted by negative samples, such that the total range is more than 10 months (> 300 days). The start of the persistent cervical HPV infection is defined as the date of the first positive sample in the sequence.

- **Other cervical HPV infection**: other cervical HPV infections than those described above will be considered in the analysis. These infections are cervical HPV infections with duration less than 150 days and cervical HPV infections detected at the last visit and for which it is not possible to determine if they are transient or persistent.

- **Clearance of cervical HPV infection**: two definitions will be used:
  - *Definition 1*: the clearance of an cervical HPV infection, whether incident, transient or persistent, with a given HPV type is defined as the occurrence of at least **a negative sample** for HPV DNA (by PCR) of that HPV type at the evaluation performed 6 months after the evaluation when the incident or transient cervical HPV infection was detected or when the last positive sample of the 6-month or 12-month persistent cervical HPV infection was detected.
  - *Definition 2*: the clearance of an cervical HPV infection, whether incident, transient or persistent, with a given HPV type is defined as the occurrence of at least **two negative samples** for HPV DNA (by PCR) of that HPV type at the evaluation performed 6 month after the evaluation when the incident or transient cervical HPV infection was detected or when the last positive sample of the 6-month or 12-month persistent cervical HPV infection was detected.

The flowchart in Section 8.2 provides an example of the determination of cervical HPV infection endpoints for a given HPV type.

4.3.2 Histopathological CIN associated with HPV type

The section 4.4.2.1 of the RAP for the HPV-008 efficacy study (dated 8 August 2009) provides an overview of the histopathological endpoint determination process and the section 4.4.4.2 of the RAP details the case definition of histopathological endpoints.

A **histopathologically confirmed CIN** can be detected in the biopsy sample after colposcopy or in the excision specimen after treatment.

Histopathologically confirmed CIN include:

- **CIN1+**: Cervical intraepithelial neoplasia as CIN1, CIN2, CIN3, adenocarcinoma in-situ and invasive cervical cancer,

- **CIN2+**: Cervical intraepithelial neoplasia as CIN2, CIN3 adenocarcinoma in-situ and invasive cervical cancer.

- **CIN3+**: Cervical intraepithelial neoplasia as CIN3 adenocarcinoma in-situ and invasive cervical cancer.

The **histopathologically confirmed CIN associated with a given HPV type infection** will be defined at a subject level. A subject might have multiple lesions associated with different HPV types at the same time point or at different time points.

As for the HPV-008 efficacy study, the current study will include only histopathological endpoints which were confirmed by the study Endpoint Committee.

4.4 Determinant definitions

4.4.1 Reference cervical HPV infections used for definitions

The definitions of some determinants as well as their potential inclusion in the different statistical analyses will be based (when relevant) on the cervical HPV infections referenced in the analyzed endpoint:

- For a **histopathologically-confirmed CIN2+ (or CIN1+, or CIN3+) associated with the same HPV type as a previous confirmed 6-month persistent cervical HPV infection**, the reference cervical HPV infection will be the 6-month persistent infection,
- For a **histopathologically-confirmed CIN2+ (or CIN1+, or CIN3+) associated with the same HPV type as a previous cervical HPV infection (any cervical HPV infection)** the reference cervical HPV infection will be the cervical HPV infection,
- For the **clearance of a cervical HPV infection**, the reference cervical HPV infection will be the cervical HPV infection for which the clearance is observed,
- For a **first detected incident cervical HPV infection (or a first confirmed 6-month persistent cervical HPV infection)** following the start of sexual activity, the reference cervical HPV infection will be the first cervical HPV infection (or first confirmed 6-month persistent cervical HPV infection).

4.4.2 Clinical determinants

For each reference cervical HPV infection with a given HPV type over the 48-month follow-period, the following determinants will be defined:

- HPV type: 16; 18; 31; 33; 45; Other high risk HPV type; Other low risk HPV type.

  The above categorization of HPV types will be used in the univariate and multivariable analyses. All HPV types will be considered individually in the tables presenting descriptive statistics by HPV type as well as high risk and low risk categories. The high risk (oncogenic) category is obtained by grouping together HPV types 16, 18, 31, 33, 35, 39, 45, 51, 52, 56, 58, 59, 66, and 68. a The low risk (non-oncogenic) category is obtained by grouping together HPV types 6, 11, 34, 40, 42, 43, 44, 53, 54, 70 and 74. In some cases, the category "No cervical HPV infection" will be added in the categorization for univariate and multivariable analyses.

- HPV infection category: 6-month persistent, less than 6-month persistent, transient, last visit detected.

- Previous cervical HPV infection: No; Yes, with at least one high risk HPV type; Yes, with only low risk HPV type.

- Histopathologically-confirmed CIN2+ (or CIN1+, or CIN3+) following the reference cervical HPV infection, associated with a different HPV type, and preceding the CIN2+ (or CIN1+, or CIN3+) associated with the same HPV type as the reference infection (i.e. date of CIN2+ (or CIN1+, or CIN3+) following the reference infection strictly inferior to date of CIN2+ (or CIN1+, or CIN3+) associated with the same HPV type as the reference infection): No; Yes, with at least one high risk HPV type; Yes, with only low risk HPV type.

- Histopathologically-confirmed CIN2+ (or CIN1+, or CIN3+) preceding the reference cervical HPV infection, associated with a different HPV type : No; Yes, with at least one high risk HPV type; Yes, with only low risk HPV type.

- Co-infection with other HPV types (concomitant cervical HPV infections): No; Yes, with at least one high risk HPV type; Yes, with only low risk HPV type.

  Co-infection with other HPV types is defined as the detection of these HPV types at time of the onset of the reference cervical HPV infection or, if this infection is persistent, during the duration of the reference cervical HPV infection until its end or until the occurrence of the event, whichever comes first.

  Consequently any cervical HPV infection which onset is prior to the onset of the reference cervical HPV infection will be considered as a previous cervical HPV infection and any cervical infection which onset if after the end of the reference cervical HPV infection and before the occurrence of the event will not be considered as a co-infection.

  However, when analyzed as time-varying covariables, co-infections will be considered until the occurrence of the event (e.g., lesion).

The following determinants will be defined for the purpose of analyzing immunosubset but they won't be included as covariates in the multivariable models:

- Serology status (based on ELISA) at study entry (Month 0) for HPV-16: Negative; Positive.

- Serology status (based on ELISA) at study entry (Month 0) for HPV-18: Negative; Positive.

4.4.3 Behavioral determinants

The following behavioral determinants will be defined at subject level using data from the last yearly questionnaire preceding the date of the detection of the reference cervical HPV infection:

- Cigarette Smoking: number of packs per year (one pack will be considered as equivalent to 20 cigarettes):
  - [0;0.5[pack year (women who never smoked i.e. never tried a cigarette or smoked <1 pack per day for less than 6 months) ;
  - ≥0.5 pack year (women who smoked ≥1 pack per day for less than 6 months or smoked <1 pack per day for 6 months or more or smoked ≥1 pack per day for 6 months or more)

- Age at first intercourse (years): Never had sexual intercourse; <15; 15-17; 18-25.

- Number of sexual partners: 0; 1; 2-3; ≥4.

- Marital status: Living or lived with a partner; Single.

- At least one previous pregnancy: No; Yes. Parity will also be explored.

The following determinants will be assessed at subject level using data from the CRF of the most recent visit preceding the reference cervical HPV infection:

- Hormones for contraception or another indication: No; Yes.

- Intra-uterine device: No; Yes.
- Sterilized: No; Yes.
- *Chlamydia trachomatis*: No; Yes.

  The presence of *Chlamydia trachomatis* at visits following baseline will be derived from the AEs collected in HPV-008 study using the following MedDRA codes for *Chlamydia*: '10008538', '10018185', '10053028', '10061041', '10062780', '10062784', '10064525', '10067186', '10067198'.

  In addition, results from *Chlamydia trachomatis* DNA yearly screening will also be used.

4.4.4 Other determinants

The following analysis factors will be defined at subject level using data from the CRF:

- Age groups (years) at the start date of time to event for each endpoint (cf. Section 8.5 for more details). This determinant will be analyzed primarily as a continuous variable. For exploratory purposes, it could also be categorized as followed: 15-17; 18-25.
- Geographical regions: Europe (Belgium, Finland, Germany, Italy, Spain, UK); Asia Pacific (Australia, Philippines, Taiwan, Thailand); Latin America (Brazil, Mexico); North America (Canada, United States).

4.5 Handling of missing data and inconsistencies

4.5.1 Missing HPV type at visits

The following rules will be applied for the determination of cervical HPV infections in case HPV type is missing at visits:

Visit v	Visit v+1	Visit v+2	Rule for the determination of cervical HPV infections and clearance
HPV-X	Missing HPV type	HPV-X	If the duration between detection date of HPV-x at visit v and detection of HPV-x at visit v+2 is > 150 days → 6-month persistent cervical HPV infection starting at Visit v
HPV-X	Missing HPV type	No HPV-X	Transient cervical HPV infection at Visit v<br>Clearance* (according to definition 1) of cervical HPV infection at visit v+2
HPV-X	Missing HPV type (last visit)	-	No clearance of HPV-X at visit v+1
No HPV-X	HPV-X	Missing HPV type (last visit)	Other cervical HPV infection at Visit v+1<br>No clearance of HPV-X at visit v+2

*Clearance (according to definition 2) if there is no HPV of type x at visit v+3.

4.5.2 Other missing data

Missing and inconsistent data will not be replaced. Thus, subjects from the TVC-1- Control arm cohorts with a missing endpoint measure will not be included in the analyses related to this endpoint. Likewise, subjects with a missing or inconsistent data for a determinant (from behavioral questionnaire or CRF) will not be included in the analyses using this determinant.

In case too many observations have to be excluded due to missing data, data imputation may be performed on a case by case basis for some of the determinants used in the univariate and multivariable analyses.

The choice of the most appropriate imputation method (mean substitution, use of multiple regression etc.) will be done at the time of statistical analysis. It will depend on the percentages and patterns of missing data (missing at random or not). The chosen method will be described and justified in the statistical report.

All analyses performed based on imputed missing data will be considered as sensitivity analyses aimed at confirming or complementing the main results obtained without replacement of missing data.

5 CHANGES IN THE CONDUCT OF THE STUDY OR PLANNED ANALYSES

Not applicable.

6 STATISTICAL ANALYSES

6.1 Statistical considerations

6.1.1 General considerations

All analyses/summaries will be performed using either SAS version 9.1 or higher. They will be done on all 14 countries of the study pooled together.

Categorical variables will be summarized by the frequency and the percentage of each category. The following statistics will be presented for continuous variables: number of non-missing observations, mean, standard deviation, median and range (minimum and maximum).

Unless specified otherwise, two-sided 95% confidence intervals for percentages will be computed using an exact method based on the binomial distribution (Clopper-Pearson method [Newcombe, 1998]).

6.1.2 Univariate and multivariable analyses

Except for the analysis of time to first detected incident cervical HPV infection (or first confirmed persistent 6-month cervical HPV infection), the statistical unit of univariate and multivariable analyses will be the cervical HPV infection related to the analyzed event. In order for the determinants defined at subject level to be used in the analyses, the values of

these determinants will be attributed, for each subject, to all the cervical HPV infections of the subject.

The inclusion of determinants in the models will depend on the meaning of these determinants with regards to the analyzed endpoint. For instance, when analyzing the time to first cervical HPV infection, all determinants linked to cervical HPV infections occurring after the first cervical HPV infection will not be taken into account as determinants in the analysis (cf. Section 8.5 for the lists of determinants used in the analyses).

Changes in the definitions of determinants (i.e. modifications of categorizations) may be performed at the time of the statistical analysis in case:

- one category contains too few subjects as compared to the other modalities of a same factor,
- two highly correlated factors are statistically significant in the multivariable analyses. In such case, the two factors may be combined in one single categorisation or one factor will not be used in the analyses.
- a different expression of a determinant is more meaningful for the interpretation of the results, more particularly for determinants that show an association with the outcome (e.g. the effect of cigarette smoking could be assessed using the cumulative lifetime exposure to smoking rather than using the number of packs per year),
- specific analyses are performed (e.g. analyses restricted to HPV types 16 and/or 18).

Such changes will be documented and justified in the statistical report.

Although described in the frequency distribution tables, the modality "never had sexual intercourse" for some of the behavioral determinants may not be taken into account in the univariate and multivariable analyses.

Results of multivariable analyses will be interpreted based on both statistical considerations (e.g., p-values or changes in point estimates of the determinants of interest by a given percentage when they are associated with covariates) and clinical and/or virological aspects (i.e. clinical meaning of the determinants included in the analyses with regards to the analyzed endpoint/outcome).

6.2 Statistical methods

6.2.1 Description of study cohorts

The number and percentage of subjects included in the study cohorts will be tabulated. The flowchart of the study cohorts will be presented as shown in Section 8.1.

6.2.2 Subject characteristics

Subject characteristics (age, country and geographical region) will be described at study entry for the TVC-1-Control arm cohort.

6.2.3 Description of endpoints and determinants

6.2.3.1 Endpoints and clinical determinants

Endpoint and clinical determinants defined in Section 4.3 and in Section 4.4.2 will be summarized over the 48-month follow-up period for the TVC-1-Control arm cohort.

When relevant, endpoint density estimates will be calculated by dividing the number of incident events (i.e. progression to lesion, first cervical HPV infection) by the length of follow-up in person months of subjects at risk.

The description of endpoints will additionally be presented stratified according to clinical or behavioral determinants of interest, such as HPV type.

6.2.3.2 Behavioral determinants

Behavioral determinants defined in Section 4.4.3 will be described at subject level by visit for the TVC-1-Control arm cohort by means of frequency distributions (numbers and percentages of subjects).

6.2.4 Analysis of time between persistent cervical HPV infections and lesions

6.2.4.1 Population

The population for the analysis of time between confirmed persistent cervical HPV infections (6-month definition) and histopathologically-confirmed CIN2+ (or CIN1+, or CIN3+) associated with the same HPV type will be restricted to subjects from the TVC-1-Control arm cohort for whom at least one 6-month persistent cervical HPV infection was detected so that a starting date is available for the computation of the time between persistent infections and lesions.

Subjects with positive or missing HPV DNA at Month 0 for the HPV type associated with the endpoint will be taken into account in the analysis as well as subjects for whom a histopathologically-confirmed CIN2+ (or CIN1+, or CIN3+) associated with a given HPV type is detected prior to the detection of the first 6-month persistent cervical infection of that HPV type (see Sections 6.2.4.3 and 6.2.4.4 for handling this particular case).

The cervical HPV infections of subjects positive for HPV DNA at Month 0 and negative for HPV DNA at Month 6 (for the HPV type associated with the endpoint) will be considered as transient cervical HPV infections. As a matter of fact, such cervical HPV infections may be either transient or persistent but the start date of infections which started before study entry is unknown. Consequently, these infections will not be taken into account in the analyses.

6.2.4.2 Analysis unit

The statistical unit for this analysis will be the 6-month persistent cervical HPV infection for which it is assessed that a following histopathologically-confirmed CIN2+ (or CIN1+, or CIN3+) associated with the same HPV type is detected.

6.2.4.3 Event variable

The analyzed event - histopathologically-confirmed CIN2+ (or CIN1+, or CIN3+) associated with the same HPV type as the 6-month persistent cervical HPV infection - will be analyzed by means of a binary variable with the following categories:

- 1 = Detection of a histopathologically-confirmed CIN2+ (or CIN1+, or CIN3+) associated with the same HPV type as the 6-month persistent cervical HPV infection
- 0 = No detection of a histopathologically-confirmed CIN2+ (or CIN1+, or CIN3+) associated with the same HPV type as the 6-month persistent cervical HPV infection or no detection of a histopathologically-confirmed CIN2+ (or CIN1+, or CIN3+)

In case more than one histopathologically-confirmed CIN2+ (or CIN1+, or CIN3+) associated with a confirmed 6-month persistent cervical HPV infection are detected, only the first CIN2+ (or CIN1+, or CIN3+) in chronological order will be taken into account, whether the histopathologically-confirmed CIN2+ (or CIN1+, or CIN3+) is detected prior, i.e. within the 12 months preceding the confirmed 6-month persistent cervical HPV infection or after this infection.

The event will be determined as showed with the following examples (See section 8.4.1 for more details):

**Sub-ject**	**Detected cervical HPV infections* and/or lesions over the follow-up period**	**Subject included in analysis population**	**Event (0 = No / 1 = Yes)**
A	-	No	
B	Transient infection associated with HPV type X<br>CIN associated with HPV type X	No	
C	12-month persistent infection associated with HPV type X<br>CIN associated with HPV type X	Yes	1
D	Transient infection associated with HPV type X<br>Transient infection associated with HPV type Y<br>6-month persistent infection associated with HPV type Z<br>CIN associated with HPV type X<br>CIN associated with HPV type Z	Yes	 <br>1
E	6-month persistent infection associated with HPV type X	Yes	0
F	6-month persistent infection associated with HPV type X<br>6-month persistent infection associated with HPV type Y<br>CIN associated with HPV type X	Yes	1<br>0
G	6-month persistent infection associated with HPV type X**<br>6-month persistent infection associated with HPV type X<br>CIN associated with HPV type X	Yes	0<br>1

* Cervical HPV infections of same HPV type are separated by a clearance.

** In case two distinct 6-month persistent infections (i.e. separated by a clearance), associated with the same HPV type, are detected for a same subject, the following rules will apply:

- the event will be associated with the first detected infection (in chronological order) if a CIN2+ (or CIN1+, or CIN3+) associated with this HPV type is detected after the first detected infection but before the second detected infection of same type,

- the event will be associated with the second detected infection (in chronological order) if a CIN2+ (or CIN1+, or CIN3+) associated with this HPV type is detected after the second detected infection.

(See Section 4.3.1 and flowchart in Section 8.2 for the definition and determination of incident, transient, persistent and other cervical HPV infections ; See Sections 4.3.2 to 4.3.4 for the definition of histopathologically-confirmed CIN2+ (or CIN1+, or CIN3+) associated with a given HPV type ; See Section 8.3.1 for the flowchart of data associated with this objective).

6.2.4.4 Time computation

The time between the onset of a confirmed persistent cervical HPV infection (6-month definition) and a histopathologically-confirmed CIN2+ (or CIN1+, or CIN3+) associated with the same HPV type will be calculated in days as: Date of event or Date of censoring - Date of detection of the 6-month HPV persistent infection.

The date of event will be:

- the date of the detection of the histopathologically-confirmed CIN2+ (or CIN1+, or CIN3+) associated with the same HPV type as the HPV type of the 6-month persistent cervical HPV infection,

The date of censoring will be:

- the date of Month 48 visit for subjects who attended all visits during the 48-month follow-up period and who had a histopathologically-confirmed CIN2+ (or CIN1+, or CIN3+) not associated with the same HPV type as the 6-month persistent cervical HPV infection or who did not have a histopathologically-confirmed CIN2+ (or CIN1+, or CIN3+) or during this period,
- the date of the last PCR reported at the latest visit for which data are available or the date of the last cytology or the date of the last biopsy (if the date is after the date of the last PCR) for subjects who did not attend all visits during the 48-month follow-up period and who had a histopathologically-confirmed CIN2+ (or CIN1+, or CIN3+) not associated with the same HPV type as the 6-month persistent cervical HPV infection or who did not have a histopathologically-confirmed CIN2+ (or CIN1+, or CIN3+) throughout their follow-up period (from first to last visit).
- Special cases:
  - In case two distinct 6-month persistent cervical HPV infections (i.e. separated by a clearance) associated with the same HPV type are detected for a same subject and a histopathologically-confirmed CIN2+ (or CIN1+, or CIN3+) associated with this HPV type is detected after the second infection, the date of censoring of the first infection will be the date of onset of the second infection since the event (i.e. detection of a histopathologically-confirmed CIN2+ (or CIN1+, or CIN3+)) is associated with the second detected infection (in chronological order).

- In case a histopathologically-confirmed CIN2+ (or CIN1+, or CIN3+) associated with a given HPV type is detected before the detection of the first 6-month persistent cervical infection of that HPV type, the time between the 6-month persistent cervical infection and the histopathologically-confirmed CIN2+ (or CIN1+, or CIN3+) will be set to 0 (i.e. the date of the 6-month persistent infection will be that of the histopathologically-confirmed CIN2+ (or CIN1+, or CIN3+)) if the delay between the histopathologically-confirmed CIN2+ (or CIN1+, or CIN3+) and the 6-month persistent cervical infection is less than 12 months (if the delay is more than 12 months, the event is censored).

  These cases are illustrated in the following table. The associations between the histopathologically-confirmed CIN2+ (or CIN1+, or CIN3+) and confirmed 6-month persistent cervical infections are showed in bold.

Visit	Visit	Visit	Visit
No CINx	HPVx persistent 6 month	No CINx	No CINx
No CINx	**HPVx persistent 6 month**	**CINx**	/
No CINx	**HPVx persistent 6 month**	**CINx**	HPVx persistent 6 month
No CINx	HPVx persistent 6 month	**HPVx persistent 6 month**	**CINx**
CINx > 12 months before persistent HPVx	HPVx persistent 6 month	/	/
**CINx <= 12 months before persistent HPVx**	**HPVx persistent 6 month**	/	/

6.2.4.5 Statistical methods

The time between persistent cervical HPV infections and lesions will be analyzed using Kaplan-Meier method and Cox regression models.

Kaplan-Meier curves presenting the cumulative probability of lesions following 6-month persistent cervical HPV infections will be displayed and compared between groups for determinants of interest using the Log-rank test (univariate analyses). Cox regression models will be also be performed for the univariate analyses in order to obtain unadjusted hazards ratios of the determinants of interest.

In addition, a multivariable Cox regression model will be performed in order to estimate the relative contribution of each determinant adjusting for the simultaneous effects of the other covariates. The model will include: time-independent covariates (e.g., age and region at study entry), time-independent covariates re-assessed at follow-up visits (e.g., behavioral determinants updated over the follow-up period using the yearly behavioral questionnaires) and time-dependent covariates (e.g., concomitant cervical HPV infections, i.e. co-infection). Also, some of the determinants will be distinguished depending on whether they started

before or after the 6-month persistent cervical HPV infections leading to the lesions in order to differentiate their effects in the pathway leading from cervical HPV infection to lesion (cf. Sections 4.4.2 and 4.4.3 for the definition of behavioral and clinical determinants and Section 8.5.1 for the list of determinants that will be taken into account in the analysis).

As the same subject may contribute to more than one observation (e.g., the same women may have several persistent cervical HPV infections of same or different HPV types leading or not to lesions associated with these HPV types), the analysis will be performed using an appropriate approach (counting process, clustering etc.) carried out either by a standard or a stratified Cox model depending on whether i) the recurrent events may be considered as identical or different and ii) the assumption that the estimated proportional hazards are constant over time is fulfilled or not [Andersen et al., 1993; Prentice et al, 1981; Kelly and Lim, 2000; Kleinbaum and Klein, 2005].

As the different events from a same subject are treated as independent observations in the Cox model, robust variance estimates adjusted for the correlation within subjects will be obtained using the robust estimation method derived as an extension of the information sandwich estimator [Lin and Wei, 1989; Zeger and Liang, 1986].

The choice of the best fitted model will be based on the Akaike's Information Criterion (AIC) and the Schwarz Bayesian Criterion (SBC) but also on the consistency of the effects of the covariates observed in the different multivariable models.

Results from the multivariable time survival analyses (Cox model) will include mean and median (if estimable) time (with 95% CI) and hazards ratios (with 95% CI). The survival curves predicted by the model will be displayed.

6.2.5 Analysis of time between any cervical HPV infections and lesions

6.2.5.1 Population

The population for the analysis of time between any cervical HPV infections and histopathologically-confirmed CIN2+ (or CIN1+, or CIN3+) associated with the same HPV type will be restricted to subjects from the TVC-1-Control arm cohort for whom at least one cervical HPV infection (any cervical HPV infection) was detected so that a starting date is available for the computation of the time between infections and lesions.

Subjects with positive or missing HPV DNA at Month 0 for the HPV type associated with the endpoint will be taken into account in the analysis as well as subjects for whom a histopathologically-confirmed CIN2+ (or CIN1+, or CIN3+) associated with a given HPV type is detected prior to any cervical infection of that HPV type.

The cervical HPV infections of subjects positive for HPV DNA at Month 0 and negative for HPV DNA at Month 6 (for the HPV type associated with the endpoint) will be considered as transient cervical HPV infections. This is done so that these infections can be taken into account in the analyses. As a matter of fact, such cervical HPV infections may be either transient or persistent but the start date of infections which started before study entry is unknown.

6.2.5.2 Analysis unit

The statistical unit for this analysis will be the cervical HPV infection for which it is assessed that a following histopathologically-confirmed CIN2+ (or CIN1+, or CIN3+) associated with the same HPV type is detected.

6.2.5.3 Event variable

The analyzed event - histopathologically-confirmed CIN2+ (or CIN1+, or CIN3+) associated with the same HPV type as the cervical HPV infection - will be analyzed by means of a binary variable with the following categories:

- 1 = Detection of a histopathologically-confirmed CIN2+ (or CIN1+, or CIN3+) associated with the same HPV type as the cervical HPV infection
- 0 = No detection of a histopathologically-confirmed CIN2+ (or CIN1+, or CIN3+) associated with the same HPV type as the cervical HPV infection or no detection of a histopathologically-confirmed CIN2+ (or CIN1+, or CIN3+)

In case more than one histopathologically-confirmed CIN2+ (or CIN1+, or CIN3+) associated with a cervical HPV infection are detected, only the first CIN2+ (or CIN1+, or CIN3+) in chronological order will be taken into account, whether the histopathologically-confirmed CIN2+ (or CIN1+, or CIN3+) is detected prior, i.e. within the 12 months preceding the cervical HPV infection or after this infection.

The event will be determined as showed with the following examples (See section 8.4.2 for more details):

**Sub-ject**	**Detected cervical HPV infections* and/or lesions over the follow-up period**	**Subject included in analysis population**	**Event (0 = No / 1 = Yes)**
A	-	No	
B	Transient infection associated with HPV type X<br>CIN associated with HPV type X	Yes	1
C	12-month persistent infection associated with HPV type X<br>CIN associated with HPV type X	Yes	1
D	Transient infection associated with HPV type X<br>Transient infection associated with HPV type Y<br>6-month persistent infection associated with HPV type Z<br>CIN associated with HPV type X<br>CIN associated with HPV type Z	Yes	1<br><br>1
E	6-month persistent infection associated with HPV type X	Yes	0
F	6-month persistent infection associated with HPV type X<br>6-month persistent infection associated with HPV type Y<br>CIN associated with HPV type X	Yes	1<br>0

Sub-ject	Detected cervical HPV infections* and/or lesions over the follow-up period	Subject included in analysis population	Event (0 = No / 1 = Yes)
G	6-month persistent infection associated with HPV type X**<br>6-month persistent infection associated with HPV type X<br>CIN associated with HPV type X	Yes	0<br>1

* Cervical HPV infections of same HPV type are separated by a clearance.

** In case several cervical HPV infections (separated by a clearance), associated with the same HPV type, are detected for a same subject, the following rules will apply:

- the event will be associated primarily with persistent 6-month cervical infections, then with persistent cervical infections which durations are less than 150 days, then with transient infections and then with other infections.

- if several cervical HPV infections of the same kind are detected for the association with the event, the event will be associated with the immediate preceding (in chronological order) detected infection if a CIN2+ (or CIN1+, or CIN3+) associated with this HPV type is detected after this infection but before the following detected infections of same type.

(See Section 4.3.1 and flowchart in Section 8.2 for the definition and determination of incident, transient, persistent and other cervical HPV infections ; See Sections 4.3.2 to 4.3.4 for the definition of histopathologically-confirmed CIN2+ (or CIN1+, or CIN3+) associated with a given HPV type ; See Section 8.3.2 for the flowchart of data associated with this objective).

6.2.5.4 Time computation

The time between the onset of a cervical HPV infection (any HPV infection) and a histopathologically-confirmed CIN2+ (or CIN1+, or CIN3+) associated with the same HPV type will be calculated in days as: Date of event or Date of censoring - Date of detection of the cervical HPV infection.

The date of event will be:

- the date of the detection of the histopathologically-confirmed CIN2+ (or CIN1+, or CIN3+) associated with the same HPV type as the HPV type of the cervical HPV infection,

The date of censoring will be:

- the date of Month 48 visit for subjects who attended all visits during the 48-month follow-up period and who had a histopathologically-confirmed CIN2+ (or CIN1+, or CIN3+) not associated with the same HPV type as the cervical HPV infection or who did not have a histopathologically-confirmed CIN2+ (or CIN1+, or CIN3+) or during this period,
- the date of the last PCR reported at the latest visit for which data are available or the date of the last cytology or the date of the last biopsy (if the date is after the date of the last PCR) for subjects who did not attend all visits during the 48-month follow-up period and who had a histopathologically-confirmed CIN2+ (or CIN1+, or CIN3+) not associated with the same HPV type as the cervical HPV infection or who did not have a

histopathologically-confirmed CIN2+ (or CIN1+, or CIN3+) throughout their follow-up period (from first to last visit).

- Special cases:
  - In case two distinct cervical HPV infections (i.e. separated by a clearance) associated with the same HPV type are detected for a same subject and a histopathologically-confirmed CIN2+ (or CIN1+, or CIN3+) associated with this HPV type is detected after the second infection, the date of censoring of the first infection will be the date of onset of the second infection since the event (i.e. detection of a histopathologically-confirmed CIN2+ (or CIN1+, or CIN3+)) is associated with the second detected infection (in chronological order).
  - In case a histopathologically-confirmed CIN2+ (or CIN1+, or CIN3+) associated with a given HPV type is detected before the detection of the first cervical infection of that HPV type, the time between the cervical infection and the histopathologically-confirmed CIN2+ (or CIN1+, or CIN3+) will be set to 0 (i.e. the date of the infection will be that of the histopathologically-confirmed CIN2+ (or CIN1+, or CIN3+)) if the delay between the histopathologically-confirmed CIN2+ (or CIN1+, or CIN3+) and the cervical HPV infection is less than 12 months (if the delay is more than 12 months, the event is censored).

  These cases are illustrated in the following tables. The associations between the histopathologically-confirmed CIN2+ (or CIN1+, or CIN3+) and confirmed 6-month persistent cervical infections are showed in bold.

Whatever the order of the cervical HPV infections	**a**	**b**	**c**	**d**	**e**
-	No CINx	HPVx persistent 6 month	HPVx persistent < 6 month	HPVx transient	HPVx other
b-a <= 12 mo<br>c-a <= 12 mo or c-a > 12 mo or no c<br>d-a <= 12 mo or d-a > 12 mo or no d<br>e-a <= 12 mo or e-a > 12 mo or no e	**CINx**	**HPVx persistent 6 month**	HPVx persistent < 6 month	HPVx transient	HPVx other
b-a > 12 mo or no b<br>c-a <= 12 mo<br>d-a <= 12 mo or d-a > 12 mo or no d<br>e-a <= 12 mo or e-a > 12 mo or no e	**CINx**	HPVx persistent 6 month	**HPVx persistent < 6 month**	HPVx transient	HPVx other
b-a > 12 mo or no b<br>c-a > 12 mo or no c<br>d-a <= 12 mo<br>e-a <= 12 mo or e-a > 12 mo or no e	**CINx**	HPVx persistent 6 month	HPVx persistent < 6 month	**HPVx transient**	HPVx other
b-a > 12 mo or no b<br>c-a > 12 mo or no c<br>d-a > 12 mo or no d<br>e-a <= 12 mo	**CINx**	HPVx persistent 6 month	HPVx persistent < 6 month	HPVx transient	**HPVx other**
b-a > 12 mo or no b<br>c-a > 12 mo or no c<br>d-a > 12 mo or no d<br>e-a > 12 mo or no e	CINx	HPVx persistent 6 month	HPVx persistent < 6 month	HPVx transient	HPVx other

Whatever the order of the cervical HPV infections	**b**	**c**	**d**	**e**	**a**
	HPVx persistent 6 month	HPVx persistent < 6 month	HPVx transient	HPVx other	No CINx
b<br>c or no c<br>d or no d<br>e or no e	**HPVx persistent 6 month**	HPVx persistent < 6 month	HPVx transient	HPVx other	**CINx**
no b<br>c<br>d or no d<br>e or no e	HPVx persistent 6 month	**HPVx persistent < 6 month**	HPVx transient	HPVx other	**CINx**
no b<br>no c<br>d<br>e or no e	HPVx persistent 6 month	HPVx persistent < 6 month	**HPVx transient**	HPVx other	**CINx**
no b<br>no c<br>no d<br>e	HPVx persistent 6 month	HPVx persistent < 6 month	HPVx transient	**HPVx other**	**CINx**

6.2.5.5 Statistical methods

The time between any cervical HPV infections and lesions will be analyzed using the same models and methodology as for the analysis of the time between persistent cervical HPV infections and lesions (cf. Section 6.2.4.5).

6.2.6 Analysis of clearance of cervical HPV infections

6.2.6.1 Population

The population for the analysis of the clearance of a cervical HPV infection will be restricted to subjects from the TVC-1-Control arm cohort for whom at least one cervical HPV infection was detected over the follow-up period so that a clearance of absence of clearance can be assessed.

6.2.6.2 Analysis unit

The statistical unit for this analysis will be the cervical HPV infection for which the clearance is assessed.

6.2.6.3 Dependent variable

For both definitions of the clearance of cervical HPV infection presented in Section 4.3.1, the clearance of an incident cervical HPV infection will be analyzed by means of a binary variable with the following categories:

- 0 = No clearance of the cervical HPV infection
- 1 = Clearance of the cervical HPV infection

(See Section 4.3.1 and flowchart in Section 8.2 for case definitions and determination of incident cervical HPV infections and their clearance; See Section 8.3.3 for the flowchart of data associated with this objective).

6.2.6.4 Statistical methods

The clearance of cervical HPV infections (two definitions) will be analyzed using marginal binary-response models such as generalized estimating equation (GEE) regression models for clustered binary responses [Zeger et al., 1988].

These multivariable analyses will allow taking into account that women may contribute to several observations related to different HPV types, resulting in multiple event times, in addition to estimating the simultaneous effects of determinants. The GEE approach will adjust for the dependency caused by the repeated observations on the same subject by estimating robust standard errors for parameter estimates. The appropriate correlation matrix structure accounting for within-subject correlation will be determined at the time of the statistical analysis based on the patterns observed.

Interactions between determinants of interest and the type of cervical HPV infection will be explored by introducing interaction terms between these determinants and a variable categorizing the type of cervical HPV infections as transient or other, 6-month persistent and 12-month persistent (See Sections 4.4.2 and 4.4.3 for the definition of behavioral and clinical determinants and Section 8.5.2 for the list of determinants that will be taken into account in the analysis). In the presence of strong interactions, the analyses will be stratified according to the type of cervical HPV infection.

Results from the multivariable regression models will include odds ratios (95% CI).

Depending on the results, other models taking into account the time between cervical HPV infections and their clearance (i.e. Cox regression models) may be used also as described in Section 6.4.2.5. In such case, cervical HPV infection detected at the last visit will be excluded of the analysis because they will all be censored at day 0.

6.2.7 Analysis of time to first detected cervical HPV infections

6.2.7.1 Population

The population for the analysis of time between the start of sexual activity and a first detected incident cervical HPV infection (or a first confirmed 6-month cervical HPV infection) will be restricted to subjects from the TVC-1-Control arm cohort for whom the date of start of sexual activity is known (i.e. either collected or computed, so that a starting date is available for the computation of the time between the start of sexual activity and a first detected cervical HPV infection) and for whom the date of start of sexual activity is either less than 6 months prior to their inclusion in the study or during the follow-up period.

Subjects with cervical HPV infections preceding the start of sexual activity will not be taken into account in the analyses.

6.2.7.2 Analysis unit

The statistical unit for this analysis will be the subject.

6.2.7.3 Event variable

The analyzed event - first incident cervical HPV infection (or a first confirmed 6-month cervical HPV infection) - will be analyzed by means of a binary variable with the following categories:

- 1 = First detected cervical infection following the start of sexual activity
- 0 = No detection of cervical HPV infection following the start of sexual activity

The event will be determined as showed with the following examples (See section 8.4.3 for more details):

Sub-ject*	Detected cervical HPV infections** over the follow-up period (in chronological order)	Subject included in analysis population	Event (first infection) (0 = No / 1 = Yes)	Event (first persistent) (0 = No / 1 = Yes)
A	-	Yes	0	0
B	Transient infection associated with HPV type X	Yes	1	0
	Transient infection associated with HPV type Y			
C	Transient infection associated with HPV type X	Yes	1	
	Transient infection associated with HPV type Y			
	12-month persistent infection associated with HPV type Y			1
D	Transient infection associated with HPV type X	Yes	1	
	Transient infection associated with HPV type Y			
	Transient infection associated with HPV type X			
	6-month persistent infection associated with HPV type X			1

* It is assumed that a date of start of sexual activity is available for all subjects.

** Cervical HPV infections of same HPV type are separated by a clearance.

(See Section 4.3.1 and flowchart in Section 8.2 for the definition and determination of incident, transient, persistent and other cervical HPV infections ; See Section 8.3.4 for the flowchart of data associated with this objective).

6.2.7.4 Time computation

The time to a first detected incident cervical HPV infection (or a first confirmed 6-month cervical HPV infection) following the start of sexual activity will be calculated in days as: Date of event or Date of censoring - Date of start of sexual activity.

The date of start of sexual activity will be determined as follows:

- For subjects who already had sexual intercourse prior to study entry: the date of start of sexual activity will correspond to the birthdate collected in the CRF plus the age at first sexual intercourse collected in the baseline behavioral questionnaire,.
- For subjects who did not have sexual intercourse prior to study entry: the date of start of sexual activity will correspond to the maximum between i) the date of the yearly questionnaire in which subjects reported having first sexual intercourse (or the date of the visit corresponding to the yearly questionnaire if the date of the questionnaire is missing) and ii) the date of the preceding detected cervical HPV infection, minus the date of the preceding questionnaire (or visit), divided by 2. In case there is no preceding questionnaire (i.e. baseline questionnaire is missing and first sexual intercourse is reported in the 1-year questionnaire), the date of start of sexual activity will be equal to the date of the 1-year questionnaire (or visit) minus 6 months.

The date of event will be:

- the date of the detection of the first incident cervical HPV infection (or the date of onset or the first confirmed 6-month cervical HPV infection) during the 48-month follow-up period,

The date of censoring will be:

- the date of Month 48 visit for cervical HPV infections of subjects who attended all visits during the 48-month follow-up period and did not have any cervical HPV infection during this period,
- the date of the last PCR reported at the latest visit for which data are available or the date of the last cytology (if the date is after the date of the last PCR) for cervical HPV infections of subjects who did not attend all visits during the 48-month follow-up period and who did not have any cervical HPV infection throughout their follow-up period (from first to last visit).

6.2.7.5 Statistical methods

The time to first detected incident cervical HPV infection (or first confirmed 6-month persistent cervical HPV infection) will be analyzed using the same models and methodology as for the analysis of the time between persistent cervical HPV infections and lesions (cf. Section 6.2.4.5). The analyses will only take into account the determinants starting before the first detected cervical HPV infection (cf. Sections 4.4.2 and 4.4.3 for the definition of behavioral and clinical determinants, Section 8.5.3 for the list of determinants that will be taken into account in the analysis of time to first detected incident cervical HPV infection and Section 8.5.4 for the list of determinants that will be taken into account in the analysis of time to first confirmed 6-month persistent cervical HPV infection).

6.2.8 Complementary analyses

Depending on the results of the planned analyses, complementary analyses may be conducted for the purpose of further exploring patterns or sub-groups (e.g. analyses by country). These analyses will be documented and justified in the statistical report

7 SEQUENCE OF ANALYSIS

Statistical analyses will be performed according to the following sequence:

1. Analysis of primary endpoint was delivered in August 2011 for preparation of a poster communication at the 27th International Papillomavirus Conference.
2. The full results of the endpoints described in the current analysis plan except the time to first infection (section 6.2.7) will be delivered by end 2011 to prepare a full manuscript.
3. Analysis of time to first infection (section 6.2.7) will be delivered during the first quarter 2012
4. Complementary analyses (if any) will be delivered as separate report(s).

8 REFERENCES

Paavonen J, Naud P, Salmerón J, Wheeler CM, Chow SN, Apter D, Kitchener H, Castellsague X, Teixeira JC, Skinner SR, Hedrick J, Jaisamrarn U, Limson G, Garland S, Szarewski A, Romanowski B, Aoki FY, Schwarz TF, Poppe WA, Bosch FX, Jenkins D, Hardt K, Zahaf T, Descamps D, Struyf F, Lehtinen M, Dubin G; HPV PATRICIA Study Group, Greenacre M. Efficacy of human papillomavirus (HPV)-16/18 AS04-adjuvanted vaccine against cervical infection and precancer caused by oncogenic HPV types (PATRICIA): final analysis of a double-blind, randomised study in young women. Lancet. 2009 Jul 25;374(9686):301-14. Epub 2009 Jul 6. PubMed PMID: 19586656.

Paavonen J, Jenkins D, Bosch FX, Naud P, Salmerón J, Wheeler CM, Chow SN, Apter DL, Kitchener HC, Castellsague X, de Carvalho NS, Skinner SR, Harper DM, Hedrick JA, Jaisamrarn U, Limson GA, Dionne M, Quint W, Spiessens B, Peeters P, Struyf F, Wieting SL, Lehtinen MO, Dubin G; HPV PATRICIA study group. Efficacy of a prophylactic adjuvanted bivalent L1 virus-like-particle vaccine against infection with human papillomavirus types 16 and 18 in young women: an interim analysis of a phase III double-blind, randomised controlled trial. Lancet. 2007 Jun 30;369(9580):2161-70. Erratum in: Lancet. 2007 Oct 20;370(9596):1414. PubMed PMID: 17602732.

B. Spiessens, T. Zahaf, G. Dubin, D. Descamps, F. Struyf, M. Fourneau, A phase III, double-blind, randomized, controlled, multi-center study to evaluate the efficacy of GlaxoSmithKline Biologicals' HPV-16/18 VLP/AS04 vaccine compared to hepatitis A vaccine as control in prevention of persistent HPV-16 or HPV-18 cervical infection and cervical neoplasia, administered intramuscularly according to a 0, 1, 6 month schedule in healthy females 15-25 years of age, Report Analysis Plan for the HPV-008 efficacy study, 2008.

S. Wieting, I. Martin, B. Colau, G. Dubin, D. Jenkins, P. Peeters, A. Schuind, L. Verlinden, T. Zahaf, A phase III, double-blind, randomized, controlled, multi-center study to evaluate the efficacy of GlaxoSmithKline Biologicals' HPV-16/18 VLP/AS04 vaccine compared to hepatitis A vaccine as control in prevention of persistent HPV-16 or HPV-18 cervical infection and cervical neoplasia, administered intramuscularly according to a 0, 1, 6 month schedule in healthy females 15-25 years of age, Protocol amendment 2 for the HPV-008 study, 2005.

GlaxoSmithKline Biologicals Clinical Study Report for final analysis, amendment 1 580299/008 (HPV-008). A phase III, double-blind, randomized, controlled, multi-centre study to evaluate the efficacy of GlaxoSmithKline Biologicals' HPV-16/18 VLP AS04 vaccine compared to hepatitis A vaccine as control in prevention of persistent HPV-16 or HPV-18 cervical infection and cervical neoplasia, administered intramuscularly according to a 0, 1, 6 month schedule in healthy females 15-25 years of age. Report Date May 2010.

GlaxoSmithKline Biologicals Clinical Study Report for end-of-study analysis 580299/008 (HPV-008). A phase III, double-blind, randomized, controlled, multi-centre study to evaluate the efficacy of GlaxoSmithKline Biologicals' HPV-16/18 VLP AS04 vaccine compared to hepatitis A vaccine as control in prevention of persistent HPV-16 or HPV-18 cervical infection and cervical neoplasia, administered intramuscularly according to a 0, 1, 6 month schedule in healthy females 15-25 years of age. Report Date October 2010.

Newcombe R.G., Two–sided confidence intervals for the single proportion: comparison of seven methods. Statistics in Medicine. (1998) 17: 857–872.

Andersen, P.K., Borgan, O., Gill, R.D., Keiding, N. Statistical Models Based on Counting Processes. Springer-Verlag, New York. (1993).

Prentice R. L., Williams B. J., Peterson A. V. On the regression analysis of multivariate failure time data. Biometrika. (1981) 68(2):373-379

Kelly KJ, Lim LYL. Survival analysis for recurrent event data: an application to childhood infectious diseases. Statistics in Medicine. (2000) 19:13-33.

Kleinbaum DG, Klein M. Survival Analysis: A Self-Learning Text, Second Edition. Statistics for Biology and Health. Springer-Verlag. New York (2005).

Lin, D.Y., Wei, L.J. The robust inference for the Cox proportional hazards model. J. Amer. Statist. Assoc. (1989) 84:1074-1078.

Zeger S.L., Liang K.Y. Longitudinal Data Analysis for Discrete and Continuous Outcomes. Biometrics. (1986) 42: 121-130.

Zeger S.L., Liang K.Y., Albert PS. Models for longitudinal data: a generalized estimating equation approach. Biometrics. (1988) 44:1049-60

Agresti A. Analysis of Ordinal Categorical Data, 2nd Edition. Wiley. (2010).

9 ANNEXES

9.1 Flowchart of study cohorts

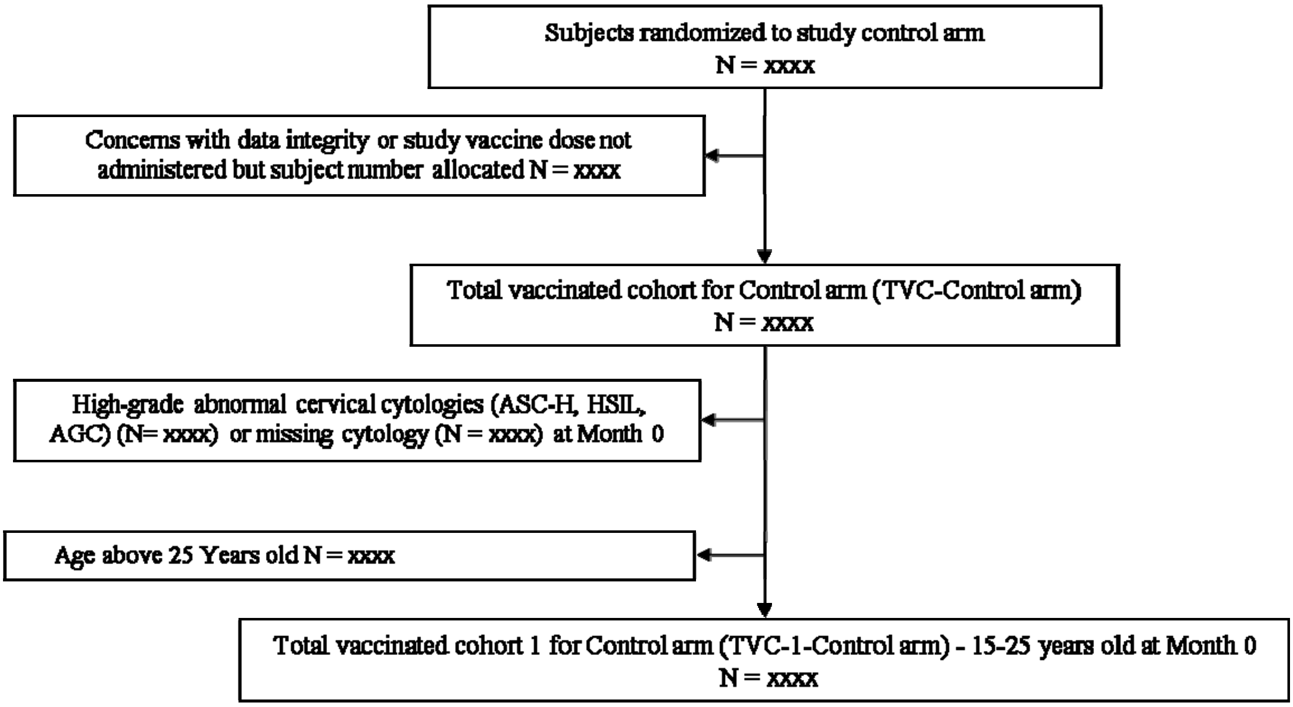

9.2 Flowchart of cervical HPV infection endpoint determination

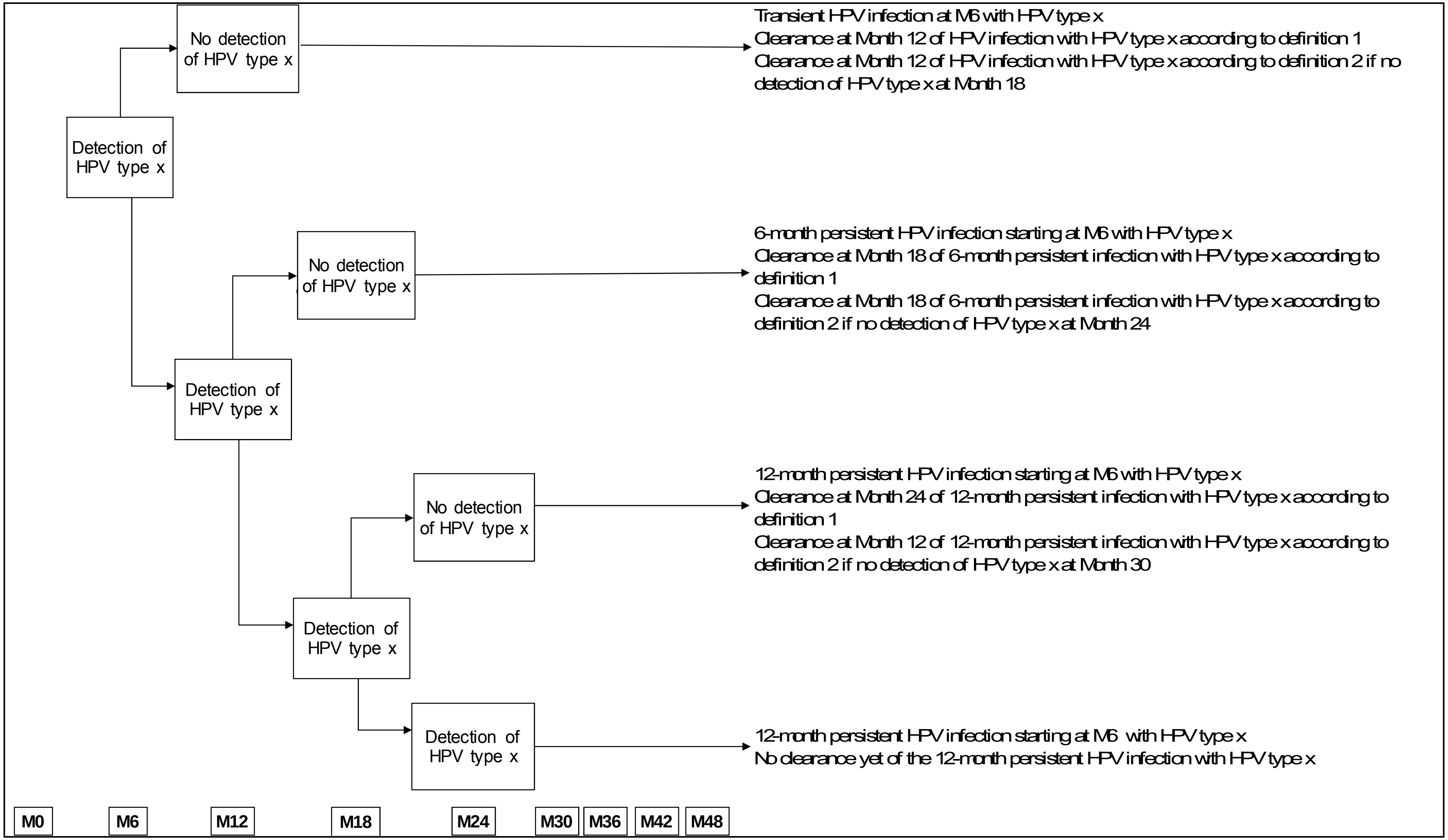

The following rules will be applied for the determination of cervical HPV infections in case a HPV type is detected at the last known visit of a patient and was not detected at the previous visits:

**Visit v**	**Visit v+1**	**Visit v+2**	**Rule for the determination of cervical HPV infections and clearance**
No HPV-X	No HPV-X	HPV-X (last visit)	Other cervical HPV infection at Visit v+2<br>No clearance of HPV-X at visit v+2

9.3 Flowcharts of data according to the objectives

Depending on the objectives, the following flowcharts will be presented in the statistical report.

9.3.1 Analysis of time between persistent cervical HPV infections and lesions

One flowchart will be presented for each endpoint (either with CIN2+ or CIN1+ or CIN3+).

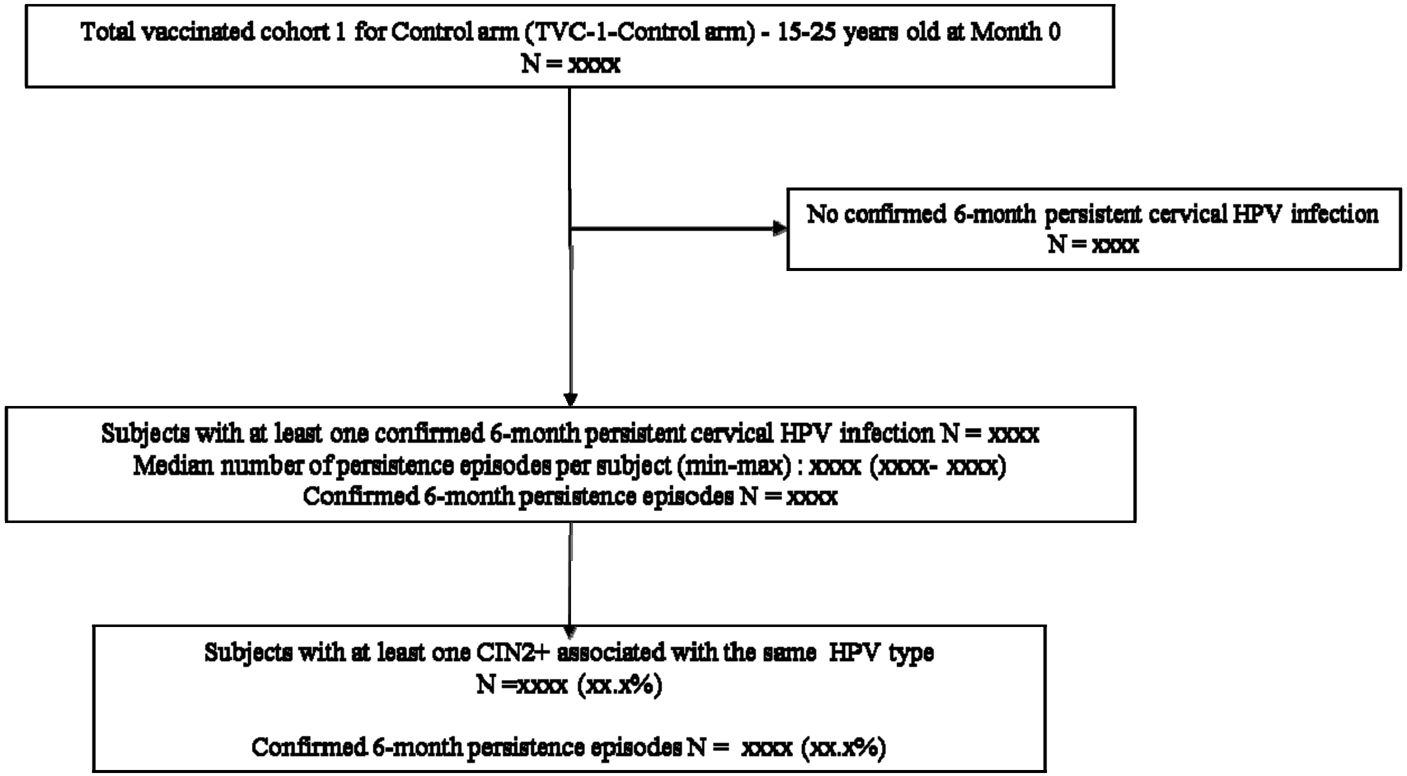

9.3.2 Analysis of time between any cervical HPV infections and lesions

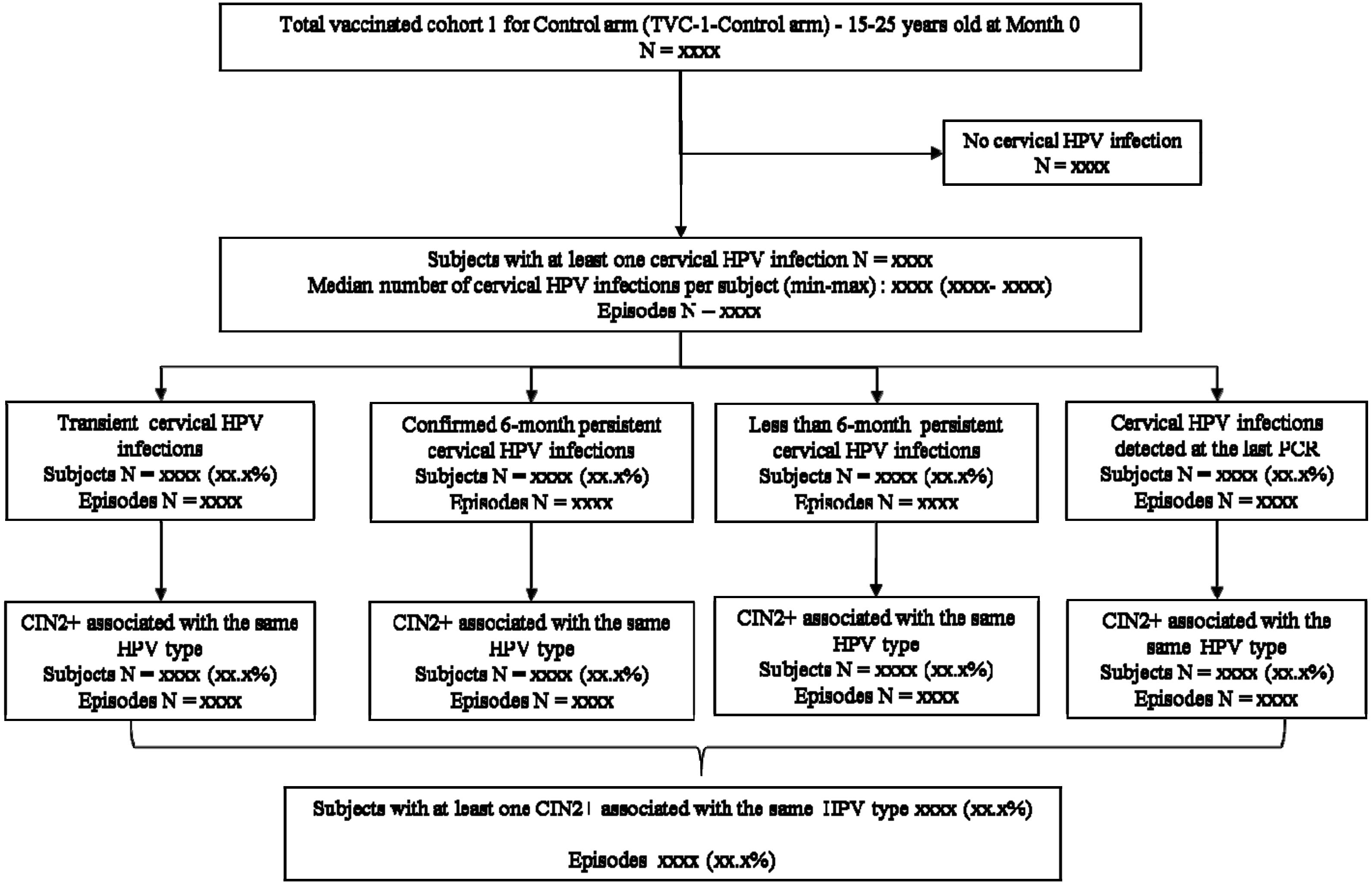

9.3.3 Analysis of clearance of cervical HPV infections

One flowchart will be presented for each the two definitions of clearance of HPV cervical infections.

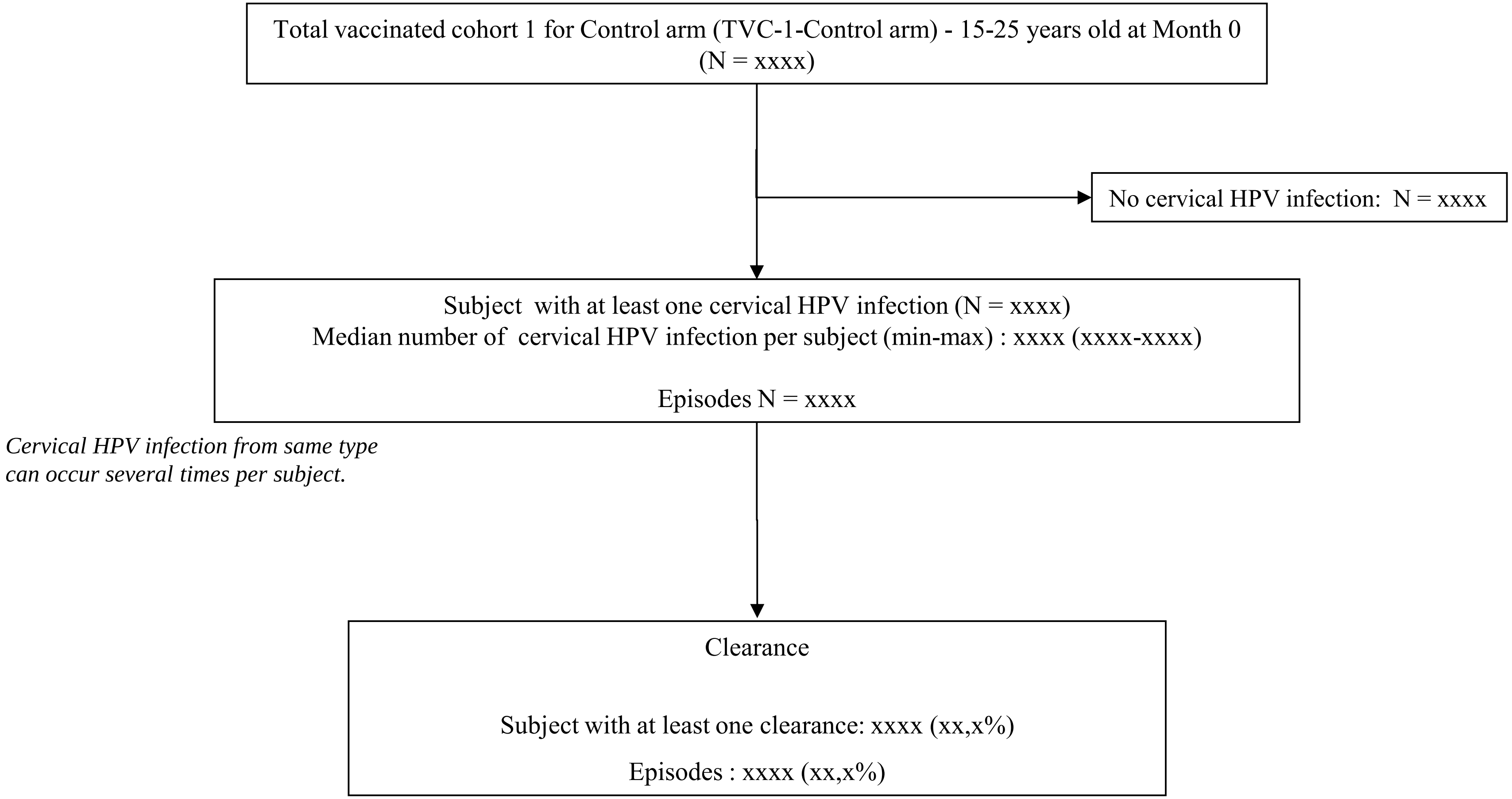

All infections are taken into account.

9.3.4 Analysis of time to first detected cervical HPV infections

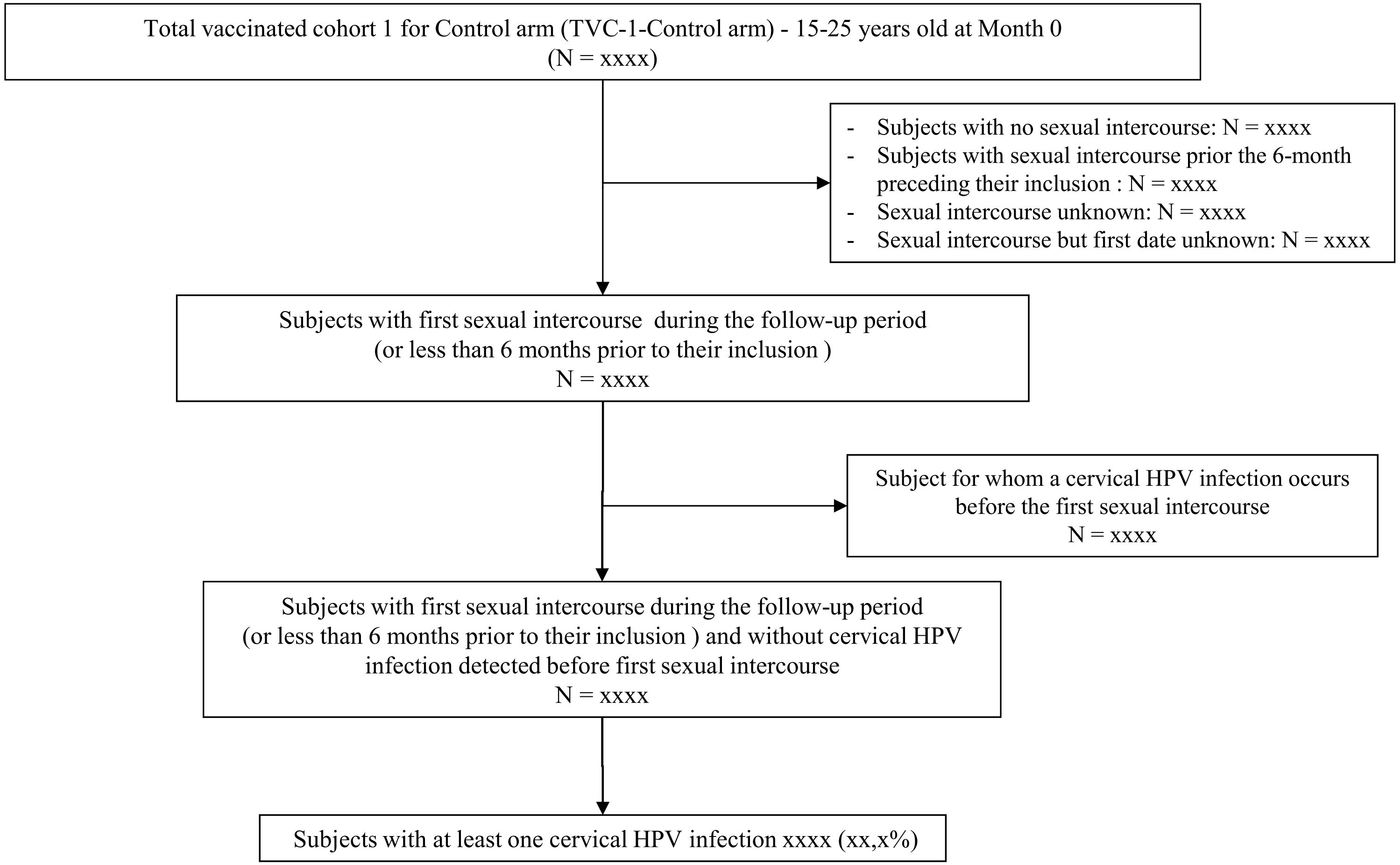

9.4 Determination of events and time to event

9.4.1 Analysis of time between persistent cervical HPV infections and lesions

	Month 0	Month 6	Month 12	Month 24	Month 30	Month 36	Month 42	Month 48	Included	Event	Time to event	Description
ID									(y/n)	(y/n)	(months)	
#1		HPV 16	HPV 16		CIN3 (HPV16)				y	y	24	• Subject #1 had a 6-month persistent HV16 cervical infection detected at month 6 and a CIN3 associated with the same HPV type at month 30.<br>• One persistent cervical HPV infection is included in the analysis with a time-to-event value of 24 months and HPV type = 16.
#2		HPV16							n	NA		• Subject #2 had a transient HV16 cervical infection detected at month 6 but no 6-month persistent cervical HPV infection throughout the 48-month follow-up period.<br>• No persistent cervical HPV infection is included in the analysis (subject not included in the analysis).
#3			HPV 16	HVP16					y	n	36 (censored)	• Subject #3 had a 6-month persistent HV16 cervical infection detected at month 12 and no CIN detected throughout the 48-month follow-up period.<br>• One persistent cervical HPV infection is included in the analysis with a censored time-to-event value of 36 months and HPV type = 16.
#4				HPV18	HPV18		CIN3 (HPV33)		y	n	24 (censored)	• Subject #4 had a persistent HVP18 cervical infection detected at month 24 and a CIN3 associated with HPV33 at month 42.<br>• One persistent cervical HPV infection is included in the analysis with a censored time-to-event value of 24 months and HPV type = 18 (note: time-to-event value is censored because CIN3 is not associated with the same HPV type as the persistent cervical HPV infection).

9.4.2 Analysis of time between any cervical HPV infections and lesions

	Month 0	Month 6	Month 12	Month 24	Month 30	Month 36	Month 42	Month 48	Included	Event	Time to event	Description
ID									(y/n)	(y/n)	(months)	
#1		HPV 16	HPV 16		CIN3 (HPV16)				y	y	24	• Subject #1 had a 6-month persistent HV16 cervical infection detected at month 6 and a CIN3 associated with the same HPV type at month 30.<br>• One persistent cervical HPV infection is included in the analysis with a time-to-event value of 24 months and HPV type = 16.
#2		HPV16							y	n	42 (censored)	• Subject #2 had a transient HV16 cervical infection detected at month 6 and no CIN detected throughout the 48-month follow-up period.<br>• One transient cervical HPV infection is included in the analysis with a censored time-to-event value of 42 months and HPV type = 16
#3			HPV 16	HVP16					y	n	36 (censored)	• Subject #3 had a 6-month persistent HV16 cervical infection detected at month 12 and no CIN detected throughout the 48-month follow-up period.<br>• One persistent cervical HPV infection is included in the analysis with a censored time-to-event value of 36 months and HPV type = 16.
#4				HPV18	HPV18		CIN3 (HPV33)		y	n	24 (censored)	• Subject #4 had a persistent HVP18 cervical infection detected at month 24 and a CIN3 associated with HPV33 at month 42.<br>• One persistent cervical HPV infection is included in the analysis with a censored time-to-event value of 24 months and HPV type = 18 (note: time-to-event value is censored because CIN3 is not associated with the same HPV type as the persistent cervical HPV infection).

9.4.3 Analysis of time to first detected cervical HPV infections

	Month 0	Month 6	Month 12	Month 24	Month 30	Month 36	Month 42	Month 48	Included	Event	Time to event	Description
ID									(y/n)	(y/n)	(months)	
#1	◊ (12 months prior month 0)	HPV 16	HPV 16						n	NA		• Subject #1 reported beginning of sexual activity one year before month 0. She had a first 6-month persistent HV16 cervical infection detected at month 6.<br>• She is not included in the analysis.
#2	◊ (6 months prior month 0)								y	n	54 (censored)	• Subject #2 reported beginning of sexual activity 6 months before month 0. She did not have any cervical HPV infection throughout the 48-month follow-up period.<br>• She is included in the analysis with a censored time-to-event value of 54 months and HPV type = "No cervical HPV infection".
#3		◊		HVP16					y	n	18	• Subject #3 reported beginning of sexual activity at month 6. She had a first transient HV16 cervical infection detected at month 24.<br>• She is included in the analysis of time to first incident cervical HPV infection (but not in the analysis of time to first 6-month persistent cervical HPV infection) with a time to event value of 18 months and HPV type = 16.
#4				HPV18					n	NA	NA	• Subject #4 did not report beginning of sexual activity at any visit.<br>• She not included in the analysis.
#5		◊		HPV16			HPV18		y	y	18	• Subject #3 reported beginning of sexual activity at month 6. She had a first transient HV16 cervical infection detected at month 24 and a first transient HV18 cervical infection detected at month 42.<br>• She is included in the analysis She is included in the analysis of time to first incident cervical HPV infection (but not in the analysis of time to first 6-month persistent cervical HPV infection) one with a time to event value of 18 months and HPV type = 16.

◊: beginning of sexual activity reported in behavioral questionnaire.

9.5 Determinants taken into account in the analyses

9.5.1 Analysis of time between persistent (or any) cervical HPV infections and lesions

	Covariates	Type	Categories	Time-dependent	Comment
Main covariate	HPV type	Class	HPV 16<br>HVP 18<br>HPV 31<br>HVP 33<br>HPV 45<br>Other high risk HPV type (35, 39, 51, 52, 56, 58, 59, 66, 68)<br>Other low risk HPV type (6,11, 34, 40, 42, 43, 44, 53, 54, 70, 74)	No	Main variable of interest
Demographic characteristics<br>*(defined at subject level)*	Region	Class	Europe<br>Asia Pacific<br>Latin America<br>North America	No	
Behavioral Covariates<br>*(defined at subject level)*	Smoking	Ordinal	[0-0.5[pack/year<br>≥0.5 pack/year	No	Re-assessed at yearly visits
	Age at beginning of sexual activity (years)	Ordinal	Never had sexual intercourse<br><15<br>15-17<br>18-25	No	
	Number sexual partners during the previous year	Ordinal	0<br>1<br>2-3<br>≥4	No	Re-assessed at yearly visits
	Marital status	Binary	Living or lived with a partner<br>Single	No	Re-assessed at yearly visits

	Covariates	Type	Categories	Time-dependent	Comment
	At least one previous pregnancy	Binary	No<br>Yes	No	Re-assessed at each visit
	Hormones for contraception or another indication	Binary	No<br>Yes	No	Re-assessed at each visit
	Intra-uterine device	Binary	No<br>Yes	No	Re-assessed at each visit
	Sterilized	Binary	No<br>Yes	No	Re-assessed at each visit
	Chlamydia trachomatis	Binary	No<br>Yes	No	Re-assessed at each visit
Clinical covariates	HPV infection category §	Class	6-month persistent<br>Less than 6-month persistent<br>Transient<br>Last visit detected	No	
	**Previous** cervical HPV infection	Class	No<br>Yes, with at least one high risk HPV type<br>Yes, with only low risk HPV type	No	Re-assessed at each visit
	Histopathologically-confirmed CIN1+ **following** the reference cervical HPV infection, associated with a different HPV type, and **preceding** the CIN2+ (or CIN1+, or CIN3+) associated with the same HPV type as the reference infection	Class	No<br>Yes, with at least one high risk HPV type<br>Yes, with only low risk HPV type	Yes	Re-assessed at each visit
	Histopathologically-confirmed CIN1+ **preceding** the reference cervical HPV infection, associated with a different HPV type	Class	No<br>Yes, with at least one high risk HPV type<br>Yes, with only low risk HPV type	No	Re-assessed at each visit
	**Co-infection*** with other HPV types (concomitant cervical HPV infection)	Class	No<br>Yes, with at least one high risk HPV type<br>Yes, with only low risk HPV type	Yes	Re-assessed at each visit

$ Only for the analysis of time between any cervical HPV infections and lesions.

* Co-infection with other HPV types is defined as the detection of these HPV types at time of the onset of the reference cervical HPV infection or, if this infection is persistent, during the duration of the reference cervical HPV infection until its end or until the occurrence of the event, whichever comes first.

Consequently any cervical HPV infection which onset is prior to the onset of the reference cervical HPV infection will be considered as a previous cervical HPV infection and any cervical infection which onset if after the end of the reference cervical HPV infection and before the occurrence of the event will not be considered as a co-infection.

However, when analyzed as time-varying covariables, co-infections will be considered until the occurrence of the event (e.g., lesion).

9.5.2 Analysis of clearance of cervical HPV infections

	**Covariates**	**Type**	**Categories**	**Time-dependent**	**Comment**
Main covariate	HPV type	Class	HPV 16<br>HVP 18<br>HPV 31<br>HVP 33<br>HPV 45<br>Other high risk HPV type (35, 39, 51, 52, 56, 58, 59, 66, 68)<br>Other low risk HPV type (6,11, 34, 40, 42, 43, 44, 53, 54, 70, 74)	No	Main variable of interest
Demographic characteristics<br>*(defined at subject level)*	Region	Class	Europe<br>Asia Pacific<br>Latin America<br>North America	No	
Behavioral Covariates<br>*(defined at subject level)*	Smoking	Ordinal	[0-0.5[pack/year<br>≥0.5 pack/year	No	Re-assessed at yearly visits
	Age at beginning of sexual activity (years)	Ordinal	Never had sexual intercourse<br><15<br>15-17<br>18-25	No	

	Covariates	Type	Categories	Time-dependent	Comment
	Number sexual partner during the previous year	Ordinal	0<br>1<br>2-3<br>≥4	No	Re-assessed at yearly visits
	Marital status	Binary	Living or lived with a partner<br>Single	No	Re-assessed at yearly visits
	At least one previous pregnancy	Binary	No<br>Yes	No	Re-assessed at each visit
	Hormones for contraception or another indication	Binary	No<br>Yes	No	Re-assessed at each visit
	Intra-uterine device	Binary	No<br>Yes	No	Re-assessed at each visit
	Sterilized	Binary	No<br>Yes	No	Re-assessed at each visit
	Chlamydia trachomatis	Binary	No<br>Yes	No	Re-assessed at each visit
Clinical covariates	**Previous** cervical HPV infection	Class	No<br>Yes, with at least one high risk HPV type<br>Yes, with only low risk HPV type	No	Re-assessed at each visit
	**Co-infection*** with other HPV types (concomitant cervical HPV infection)	Class	No<br>Yes, with at least one high risk HPV type<br>Yes, with only low risk HPV type	Yes	Re-assessed at each visit

* Co-infection with other HPV types is defined as the detection of these HPV types at time of the onset of the reference cervical HPV infection or, if this infection is persistent, during the duration of the reference cervical HPV infection until its end or until the occurrence of the event, whichever comes first.

Consequently any cervical HPV infection which onset is prior to the onset of the reference cervical HPV infection will be considered as a previous cervical HPV infection and any cervical infection which onset if after the end of the reference cervical HPV infection and before the occurrence of the event will not be considered as a co-infection.

However, when analyzed as time-varying covariables, co-infections will be considered until the occurrence of the event (e.g., clearance). Co-infections will be analyzed as time-dependent variable is the Cox regression models (not in the GEE models)

9.5.3 Analysis of time to first detected incident cervical HPV infections

	**Covariates**	**Type**	**Categories**	**Time-dependent**	**Comment**
Demographic characteristics *(defined at subject level)*	Region	Class	Europe<br>Asia Pacific<br>Latin America<br>North America	No	
Behavioral Covariates *(defined at subject level)*	Smoking	Ordinal	[0-0.5[pack/year<br>≥0.5 pack/year	No	
	Age at beginning of sexual activity (years)	Ordinal	Never had sexual intercourse<br><15<br>15-17<br>18-25	No	
	Number sexual partner during the previous year	Ordinal	0<br>1<br>2-3<br>≥4	No	
	Marital status	Binary	Living or lived with a partner<br>Single	No	Re-assessed at yearly visits
	At least one previous pregnancy	Binary	No<br>Yes	No	Re-assessed at each visit
	Hormones for contraception or another indication	Binary	No<br>Yes	No	Re-assessed at each visit
	Intra-uterine device	Binary	No<br>Yes	No	Re-assessed at each visit
	Sterilized	Binary	No<br>Yes	No	Re-assessed at each visit
	Chlamydia trachomatis	Binary	No<br>Yes	No	Re-assessed at each visit

9.5.4 Analysis of time to first confirmed 6-month persistent cervical HPV infections

	Covariates	Type	Categories	Time-dependent	Comment
Demographic characteristics *(defined at subject level)*	Region	Class	Europe<br>Asia Pacific<br>Latin America<br>North America	No	
Behavioral Covariates *(defined at subject level)*	Smoking	Ordinal	[0-0.5[pack/year<br>≥0.5 pack/year	No	
	Age at beginning of sexual activity (years)	Ordinal	Never had sexual intercourse<br><15<br>15-17<br>18-25	No	
	Number sexual partner during the previous year	Ordinal	0<br>1<br>2-3<br>≥4	No	
	Marital status	Binary	Living or lived with a partner<br>Single	No	Re-assessed at yearly visits
	At least one previous pregnancy	Binary	No<br>Yes	No	Re-assessed at each visit
	Hormones for contraception or another indication	Binary	No<br>Yes	No	Re-assessed at each visit
	Intra-uterine device	Binary	No<br>Yes	No	Re-assessed at each visit
	Sterilized	Binary	No<br>Yes	No	Re-assessed at each visit
	Chlamydia trachomatis	Binary	No<br>Yes	No	Re-assessed at each visit

	Covariates	Type	Categories	Time-dependent	Comment
Clinical covariates	**Previous** cervical HPV infection	Class	No<br>Yes, with at least one high risk HPV type<br>Yes, with only low risk HPV type	No	Re-assessed at each visit
